# A Pan-Cancer Ex Vivo Drug Screen Atlas for Functional Precision Oncology

**DOI:** 10.64898/2026.02.14.705918

**Published:** 2026-02-17

**Authors:** Karl Pichotta, Jessica B. White, Jeffrey F. Quinn, Anneliese Markus, Christopher Tosh, Antoine De Mathelin, Erin Coyne, Feiyang Huang, Wesley Tansey

**Affiliations:** 1 Computational Oncology, Memorial Sloan Kettering Cancer Center, New York, NY 10065, United States.; 2 Tri-Institutional PhD Program in Computational Biology and Medicine, Weill Cornell Graduate School of Medical Sciences, New York, New York, NY 10021, United States.; 3 Jacobs School of Medicine and Biomedical Sciences, SUNY University at Buffalo, Buffalo, NY 14203, United States.; 4 Department of Applied and Computational Mathematics and Statistics, University of Notre Dame, Notre Dame, IN 46556, United States.

## Abstract

Compared to immortalized cell lines, patient-derived organoids and other ex vivo models have been shown to better recapitulate patient responses to therapy. High cost and technical complexity have prevented the creation of pan-cancer ex vivo datasets, limiting comprehensive analyses and predictive modeling for ex vivo drug response. We present the Pan-PreClinical (PPC) project: a drug screen atlas of 2.1M experiments across 1,982 ex vivo samples and 3,100 drugs spanning 134 cancer indications tested across 26 studies. We develop a contrastive Bayesian model to harmonize across studies, identifying 303 tissue-specific drug sensitivities and demonstrating drug sensitivities are predictive of clinically-relevant molecular profiles. Integrating established cell line databases reveals systematic biases across 55 cancer subtypes, with cell line screens favoring drugs targeting highly proliferative cells and undervaluing cell-cell communication targets. We leverage PPC to establish an ex vivo foundation model and computational platform for scalable ex vivo cancer biology and predictive oncology.

Cancer patients with rare or treatment-resistant tumors are often left without any effective standard of care. The need to identify new treatments for these patients has led to a rapid acceleration of ex vivo model development and high throughput drug screening to identify functional vulnerabilities in these tumors arising from mechanisms not detectable through classical sequencing^1,2^. Compared to immortalized cell lines, ex vivo models like patient-derived cells (PDCs), organoids (PDOs), xenografts (PDXs), and xenograft-derived cells (PDXCs) maintain a higher fidelity match to their original human tissues^3–6^. Thus, positive drug screen hits in the ex vivo setting may have a higher likelihood of translating to successful clinical therapies.

Ex vivo screens in triple negative breast cancer^7^, fibrolamellar carcinoma^8^, glioblastoma^9,10^, pancreatic adenocarcinoma^1,4,11^, acute myeloid leukemia^12,13^, and epithelial ovarian cancers^14–16^ have revealed novel vulnerabilities and new potential personalized therapies for patients. The importance and feasibility of this discovery opportunity is increasing rapidly as large scale efforts to generate ex vivo models for drug screens accelerate within expert PI labs, in institutional organoid biobank programs^17^, and across multi-institutional consortia (e.g. the Pediatric Preclinical In Vivo Testing (PIVOT) consortium^18^). Ideally, these efforts could be streamlined to generate pan-cancer atlases analogous to the Cancer Dependency Map^19^ for immortalized cell lines. Unfortunately, ex vivo models tend to require bespoke protocols for each tissue or disease, limiting streamlined pan-cancer screen databases to date.

The preclinical success of ex vivo screens has also begun to translate into clinical trial success. The short distance between an ex vivo model and its patient of origin enables drug screens on ex vivo models to provide a personalized, functional characterization of a patient’s tumor in terms of vulnerabilities to candidate therapies. The INFORM^20^, TARGET/ZERO^21^, and NCH-FPM trials^22^, all on high-risk pediatric cancers, integrated ex vivo drug screens into the clinical decision making process and demonstrated that screen-guided treatments can improve patient outcomes. These successes have led to the rise of the new field of functional precision oncology (FPO), which posits that ex vivo screens of patient tumors can be used to guide treatment in the clinic^23^. While these studies have yielded substantial evidence for the FPO thesis, systematic analyses have been lacking due to the focused nature of each ex vivo study.

We reasoned that large-scale data integration of ex vivo drug screens would enable predictive modeling and quantitative evaluation of ex vivo drug responses. To this end, we established the Pan-PreClinical (PPC) project, a large open-resource effort to compile drug-response data from ex vivo studies. The PPC dataset currently comprises data from 26 different ex vivo studies, along with genomic, transcriptomic, chemoinformatic, and clinical data where available. All PPC data were integrated into a unified data model with a common cancer subtyping nomenclature, primary/metastasis label, and site-of-sample annotation. We developed a contrastive machine learning algorithm and statistical postprocessing pipeline to harmonize across studies and experimental conditions, enabling pan-cancer analyses. Using the harmonized results, we conducted statistical analyses that reveal tissue-specific differential drug responses, as well as site-specific variation in drug sensitivities across metastatic samples. Integrating 12 large pan-cancer cell line datasets, we found systematic differences in drug sensitivities between ex vivo models and disease-matched cell lines. We leveraged the integrated dataset to train a transformer-based foundation model capable of guiding future studies and showed that drug screen embeddings reliably predict genomic and transcriptomic properties of ex vivo samples. The PPC dataset, model results, and analyses can be explored on an interactive web portal: https://www.panpreclinical.org/

We conducted a literature review of publications involving ex vivo drug screens. Study inclusion criteria were that at least five patient-derived ex vivo samples were screened with at least ten unique compounds. We excluded studies involving only immortalized cell lines or animal models of disease. We identified and obtained data from 26 studies across three categories of patient-derived ex vivo biological modalities, namely assays of patient-derived cells and spheroids (PDCs)^9,10,14–16,20,24–29^, patient-derived organoids^1,4,11,30–36^, patient-derived xenograft-derived cells (PDXCs)^37^, and studies with data from multiple modalities^7,38–41^. For 9 of the studies, raw fluorescence or viability measurements were publicly deposited with the original publication. For the remaining 18 studies, we communicated with the original authors to retrieve and verify the raw data. All data were provided by study authors with consent for sharing previously published data. In one case, additional pre-publication data were provided without the ability to disentangle published from non-published samples. The public version of the PPC dataset omits this study, but we include it here in our analyses.

Through further communication with study authors, we retrieved clinical diagnoses, site-of-disease annotations, and, where available, patient history and tumor molecular information. Each ex vivo sample was matched to a standardized OncoTree cancer type^42^ to give fine-grained disease typing information, with distinctions made between primary and metastatic sample status. We labeled metastatic samples with the same 21-site conventions as the MSK-MET dataset^43^. For metastatic samples, we annotated both the original primary tissue site and the location of the sampled metastasis. Each ex vivo sample was annotated with available genomic, transcriptomic, and clinical information such as sex, treatment history, and overall survival. Studies varied in whether they gathered each additional modality. Regulatory restrictions further prevented us from obtaining and integrating gathered molecular information in some studies. All genomic and transcriptomic data were reprocessed using a standardized pipeline with a common reference genome (see Methods).

## The PPC project unifies ex vivo studies into a single pan-cancer atlas

**Figure 1:**
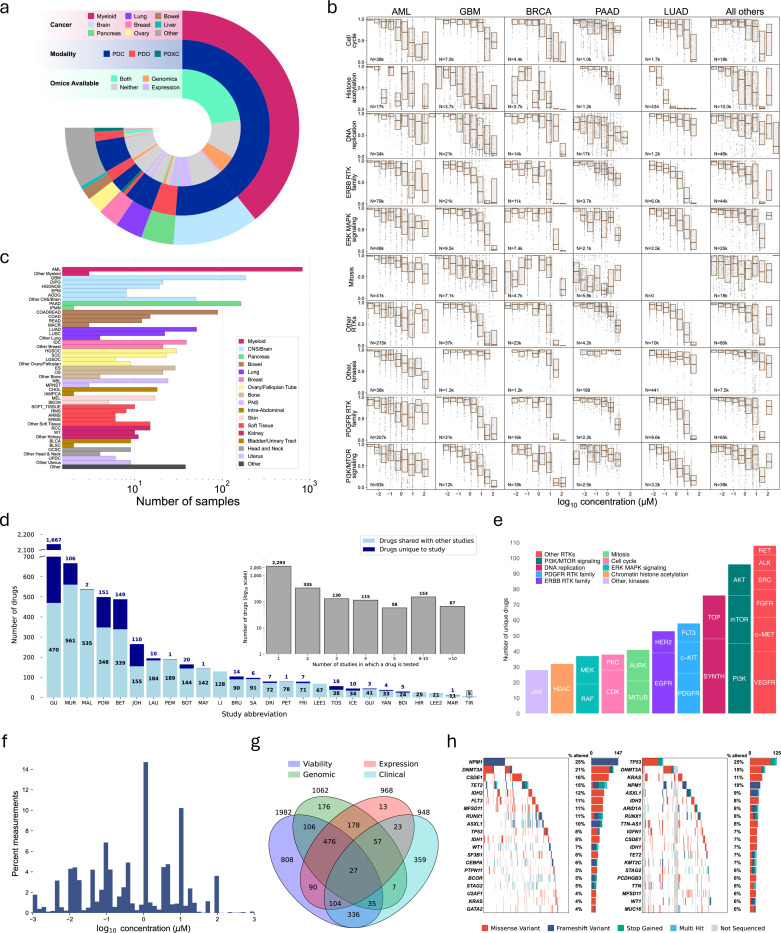
The PPC dataset represents a pan-cancer atlas of ex vivo experiments across diseases, omics, and drugs. (a) Characteristics of samples in the PPC dataset. Width of wedges represents the number of samples. The outer ring represents primary cancer site, the middle ring represents the ex vivo cell culture modality used on the sample, and the innermost ring shows whether transcriptomics and/or genomics results were available for the sample. (b) Viability measurements by OncoTree code (table S2) for top drug targets. (c) Number of samples broken down by primary diagnosis. Diagnoses are abbreviated using their OncoTree codes (tableS2). (d) Number of drugs per component study. Studies are abbreviated as specified in table S1. (e) Description of the drugs in the PPC dataset tested in >1% of all samples with the first level grouping by high-level drug category and the second level grouping by specific target. SYNTH=DNA/RNA Synthesis, TOP=Topoisomerase, MITUB=Microtubule Associated, AURK=Aurora Kinase, HEDGE=Hedgehog/Smoothened, all other target abbreviations correspond to Selleckchem mechanism of action labels (Methods). (f) Distribution of drug concentrations across experiments in the PPC dataset. (g) Number of samples annotated with viability, genomic, expression, and clinical data. Clinical data includes patient sex, approximate age at diagnosis, or overall survival. (h) OncoPrint describing mutations for the liquid (left) and solid (right) tumor samples. Excludes genes for which variants were only detected in whole genome or exome sequencing.

In total, we collected data on 3,072 ex vivo models, of which 1,982 have drug viability measurements. These represent 130 distinct cancer types (110 with viability measurements) (Fig. 1a,c), from 24 different anatomical sites (23 with viabilities), along with 53 healthy samples (40 with viabilities). Among the non-healthy samples, 2,324 are primary samples (1,834 with viabilities) and 155 are metastatic samples (108 with viabilities). The most frequent ex vivo construct type is PDCs (Fig. 1a), with 2,355 samples (1,574 with viability measurements). We also compiled 595 PDO samples (368 with viabilities) and 104 PDXC samples (37 with viabilities). Drug responses varied across cancer types and drug classes (Fig. 1b).

Using a custom iterative graph algorithm (see Methods), we harmonized drug names across studies and compiled measurements for 3,151 unique drugs and 2,133,246 individual viability measurements representing 327,335 unique dose-response curves. The median number of doses in each single-drug curve was five, though some studies employed an experimental design involving large libraries of compounds screened at a single dose; 54,351 dose-response curves comprise data at a single drug concentration (fig. S3). Each compound was annotated with FDA approval status, along with annotations for mechanisms of action (fig. S4). Drug concentrations tested in viability measurements spanned from nanomolar to millimolar ranges with a plurality taken at micromolar concentrations (Fig. 1f). Out of 26 ex vivo studies, 12 screened 100 or more compounds (Fig. 1d). Overall, 2,293 drugs (72.7%) were screened in a single study and 858 (27.2%) were screened in two or more studies (Fig. 1d); a majority of compounds tested in each study were also screened in other studies. Using a custom algorithm for drug target annotation (see Methods), we leveraged publicly available resources^44–46^ to annotate 2,219 of the 3,100 drugs with at least one target. The harmonized drug set includes a wide variety of small molecule drugs targeting many common cancer-associated proteins and biological processes and tested in >1% of samples in the PPC dataset (Fig. 1e).

We compiled and harmonized genomics data for 1,062 samples and transcriptomics data for 968 samples; 476 samples had genomics, transcriptomics, and viabilities (Fig. 1g). Five studies used whole genome or exome assays, while six studies used targeted gene panels. Four of the six studies that use gene panels assess alterations in 50 or fewer genes. Demographic and clinical annotations were comparatively limited: 948 samples had patient age, sex, overall survival, or prior treatment history; 336 had clinical annotations and viabilities (Fig. 1g). Constituent studies vary both in coverage across disease type (fig. S1) and drugs (fig. S2).

Both solid and hematological samples are available with genomic alteration data. All hematological samples with annotated genomic alterations (Fig. 1h, left) are derived from AML patients and were assessed using a 5K gene panel. The proportion of these AML samples with the most highly recurrent mutations are consistent with the levels at which they are observed in this cancer type in general, including *NPM1* (25%) and *DNMT3* (21%)^47,48^. For the remaining ten ex vivo studies with genomic alteration data for solid tumor samples, we account for differences in the genes evaluated for mutations in the underlying genomic assays (Fig. 1h, right). Most samples are evaluated for alterations in the most frequently mutated genes, including *TP*53, *DNMT*3*A*, and *KRAS*.

## Bayesian nonparametric modeling integrates PPC drug screens and generalizes to held out experiments

We sought to integrate across all the PPC component studies to produce a harmonized dataset for comparative analyses. We focused only on integrating drugs used in at least 3 studies, leaving 504 compounds. Heterogeneity in experimental designs across studies makes computational modeling and statistical analyses of the PPC dataset challenging. Overlap in drug libraries between studies varied from 0% to 94%, with an average overlap of 17%, leading to missing measurements across studies. Studies that tested drugs at only a single concentration are not compatible with a simple Hill model curve fitting approach, making full dose-response estimation, as well as summary IC_50_ or area under the curve (AUC) calculation, impossible without statistical modeling. However, while many machine learning models have been developed to predict drug responses from omics^49–52^, the majority of PPC samples do not have omics available. Further, the studies in PPC used different incubation times, culture media, and other experimental factors that lead to batch effects which existing predictive models are not designed to handle. To enable dataset-wide analyses, we developed a custom probabilistic dose-response model.

**Figure 2:**
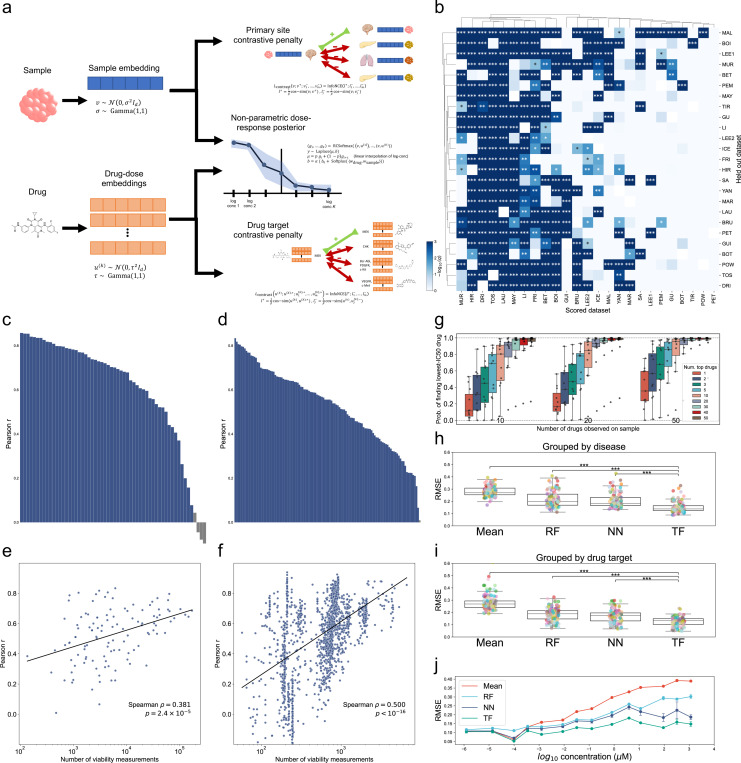
The PPC dose-response model integrates across multiple studies and generalizes to held out experiments. (a) The PPC model performs a constrained tensor factorization to predict curves and impute missing measurements. Primary site and drug mechanism annotations are used as contrastive learning labels to regularize the model. (b) Assessment of which component studies improve generalization on other component studies. Whole-drug-holdout cross-validation on each column dataset was compared with and without having each row dataset in the training set; improvement significance calculated via one-sided binomial test and BH correction. ∗∗∗: *q <* 0.001; ∗∗: *q <* 0.01; ∗: *q <* 0.1 (study abbreviations: table S1). (c,d) Cross-validation performance (Pearson correlation) stratified by (c) disease subtype and (d) drug mechanism; gray: *q >* 0.05 after BH correction (two-sided Pearson correlation t-test); abbreviations: table S2. (e,f) Cross-validation prediction by (Pearson correlation) vs. number viability measurements stratified by (e) drug mechanism and (f) sample; (*p*: two-sided Spearman rank-correlation test). (g) Power analysis benchmark evaluating the number of drugs needed in both a pilot round and a hit prediction round to achieve a target level of power to find the most efficacious drug. (h–j) Benchmarks against baseline machine learning methods, with error stratified by (h) disease, (i) drug target, and (j) log-concentration (two-sided Mann-Whitney U test with BH correction; ***: *q <* 1 × 10^−9^; Mean: bucketed mean; RF: random forest; NN: neural network; TF: PPC Bayesian tensor factorization method (Methods)); (bars: 1 standard deviation).

The PPC dose-response model performs a contrastive tensor factorization on the samples × drugs × concentrations tensor (Fig. 2a). Each ex vivo sample i is modeled as a real-valued embedding vector, $v_{i}\in {\mathbb{R}}^{d}$. For each drug j, the concentration space is divided into K discrete points and the drug is modeled as a collection $\left(u_{j}^{\left(1\right)},u_{j}^{\left(2\right)},\ldots ,u_{j}^{\left(K\right)}\right)$ of embeddings also in ${\mathbb{R}}^{d}$; concentrations falling between grid points are interpolated. The inner product of a specific drug-dose embedding and a sample embedding then represents the log-odds of increasing in viability from the previous grid point; this enforces monotonicity in the inferred curve. To reduce batch effects and inject biological knowledge into the model, the training objective includes contrastive penalties^53^ on the embeddings to encourage samples from the same tissue site to have similar embeddings and drugs with the same mechanism of action to have similar embeddings (see Methods). Once trained, the model was used to impute full monotone-down dose-response curves across the entire samples × drugs space, including sample-drug combinations unobserved during training.

Given the heterogeneity of the PPC studies, with different experimental designs and labs conducting each experiment, we first asked if data integration was even beneficial. To test this, we conducted a series of ablation studies where we held out a study and evaluated whether the removal of that study harmed or improved cross-validation performance on every other study (see Methods). We found that all datasets provide statistically significant (one-sided binomial test) empirical improvement for at least 50% of other datasets (Fig. 2b). Only one study, PET^20^, saw no statistically significant benefit from any other study though we note that four studies did contribute small improvements that were not significant after multiple testing correction (fig. S6). Studies that were found to be most helpful tended to have larger drug libraries (MAL^26^, MUR^16^) and more diverse samples (LEE1^9^, MAY^29^). The most frequently helpful studies were often the majority of the studies which benefited the least from other studies, suggesting that data integration improvements diminish as studies become broader and more expansive. However, we were only able to evaluate studies on drugs that were measured in those studies. Predicting drug responses for compounds not in the study panel still requires data integration even for the more expansive studies in the PPC dataset.

We next evaluated the model on a series of performance tasks on held-out data to test its robustness. In each task, we performed five-fold cross-validation, holding out entire dose-response curves in the test fold (see Methods). Stratifying by disease subtype, we found variable model performance, ranging from Pearson’s r=−0.10 to r=0.86, with median performance of r=0.70 (Fig. 2c, fig. S9). Across all indications, 84% (49 out of 58) had r>0.4, with none having statistically significantly negative correlations (Pearson r ). Among the diseases on which the model performed best (fig. S7) are endometrial carcinoma (UCEC), uterine sarcoma (USARC), ovarian carcinoma (OVT), and breast carcinoma (BRCA); this reflects differences in data variation, dose-concentration coverage within experiments (fig. S3), and drug coverage within constituent datasets (fig. S2).

Stratifying by mechanism of action (Fig. 2d, fig. S7), we found model performance ranged from Pearson’s r=0.01 to r=0.83, with median r=0.56. Of 116 targets measured, 87 (75%) had r>0.4. Drug classes with fewer measurements had significantly lower predictive accuracy as measured by Pearson r ( $p=2.4\times 10^{-5}$, two-sided Spearman rank-correlation, Fig. 2e, fig. S10–fig. S13). Further, we found that regions of tighter uncertainty bounds corresponded to regions of higher predictive accuracy (fig. S8), suggesting predictive uncertainty in the model is well-calibrated. For rare disease subtypes and uncommon drugs, we therefore recommend evaluating the PPC model uncertainty estimates to assess the likely fidelity of imputations. Stratifying by sample revealed that samples with more viability measurements had significantly better model accuracy as measured by Pearson r (Fig. 2f, $p<10^{-16}$, two-sided Spearman rank-correlation).

To assess the number of measurements needed to obtain a fixed performance threshold, we designed a power analysis benchmark (see Methods). Briefly, samples were held out from the PPC model during initial training, then the model was given a random subset of 10, 20, or 50 drugs as a virtual initial screen. The model was then given a budget of between 1 and 50 more drugs to predict a top hit, defined as the lowest-IC_50_ drug in the panel. We found no clear patterns emerged to suggest an optimal way to balance the initial drug budget versus the top hit prediction budget (Fig. 2g). The model obtained 80% power using two rounds of 10 drugs, 95% power using two rounds of 20 drugs, and *>* 99% power using two rounds of 50 drugs.

Finally, we sought to assess if the PPC model was exceeding the performance of traditional machine learning methods. We benchmarked the PPC model against both a naive baseline mean estimator and two machine learning methods: random forests and neural networks (see Methods). Evaluating across disease subtype and drug target, we found that the PPC model consistently outperformed all three baselines (Fig. 2h,i). This result was robust when evaluated at different concentration points on the dose-response curve (Fig. 2j), with the PPC model outperforming the baselines at every concentration level.

## Latent embedding spaces capture meaningful relationships between drug mechanisms and tumor sub-types

**Figure 3:**
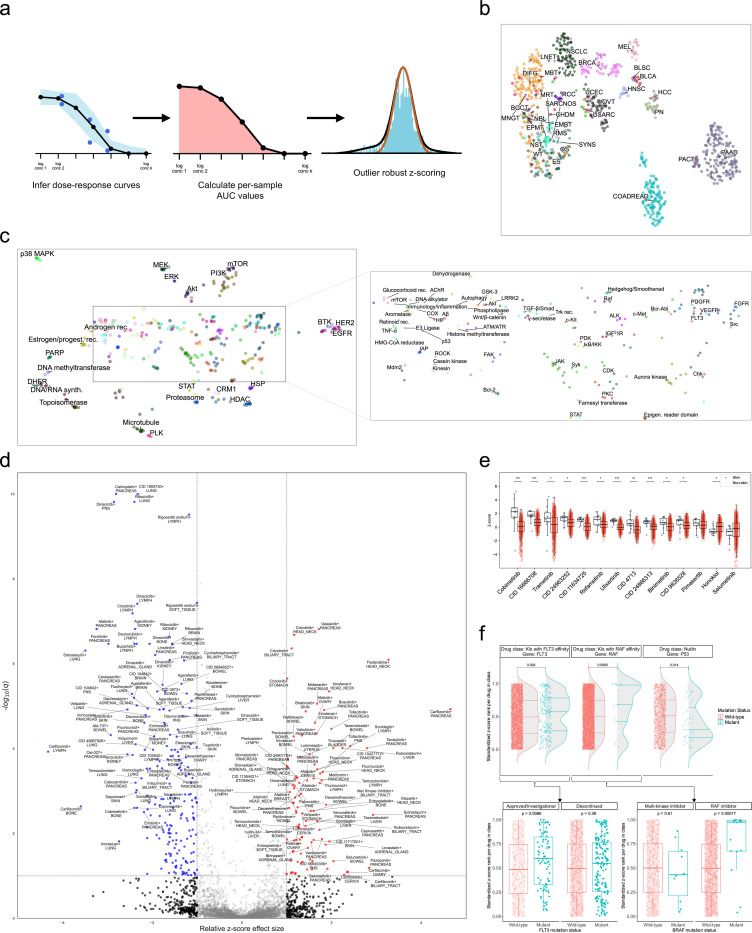
The PPC model learns biologically rational latent spaces and predicts clinically rational drug responses. (a) Batch effect-corrected z-scores are calculated on area under the dose-response curve (AUC) values using a robust null distribution procedure (see Methods). (b) UMAP of solid tumor sample embeddings colored by disease subtype. (c) UMAP of concatenated drug embeddings colored by most-common drug target. (d) Volcano plot showing top hits for primary site-grouped z-scores on each drug (x-axis: relative z-score difference, y-axis: negative *𝑙𝑜𝑔*_10_ q-value from BH corrected t-test p-values). (e) Comparison of z-scores on drugs labeled as MEK/ERK inhibitors for both skin (left) and non-skin (right) primary tumor samples. (BH-corrected p-values from two-sided Mann-Whitney U tests, *: *p <* 0.1; **: *p <* 0.01; ***: *p <* 0.001. Boxes: first and third quartiles; line: median; whiskers: 1.5 × IQR). (f) Fully-imputed and standardized per drug z-score ranks for all drugs with given mechanisms of action in the Beat AML cohort. Top: Kinase inhibitors (KIs) with FLT3 and RAF affinity and Nutlin-derived drugs in samples with and without deleterious *FLT3*, *RAF*, and *TP53* mutations, respectively. Bottom, left: FLT3 KIs by stage of clinical development. Bottom, right: RAF KIs by kinase selectivity. (*p*-value calculated via two-sided Mann-Whitney U test; Boxes: first and third quartiles; line: median; whiskers: 1.5 × IQR).

Using the trained PPC model, we fully harmonized and imputed the PPC dataset then assessed it for biological, chemical, and clinical coherence. Projecting drug and sample embeddings via UMAP^54^ indicated clear separation by both disease subtype (Fig. 3b) and drug mechanism (Fig. 3c). Related disease subtypes like BLSC and BLCA, HCC and IPN, and PAAD and PACT, all cluster together, capturing hierarchical biological structure in diseases. Similarly, drugs inhibiting components within the same pathway, including MEK/ERK and PI3K/Akt/mTOR, cluster together. Therapeutics targeting sex hormones, including androgens and estrogens, are in close proximity which are in turn near PARP inhibitors that are most often used to treat ovarian, breast, prostate, and pancreatic cancer sub-types^55^. Mitotic targets like microtubules and PLK are also clustered near metabolic targets suggesting that the model learns to differentiate agents most effective in rapidly dividing constructs.

## De-batched drug sensitivities show systematic differences across tissue sites

Assessing drug sensitivities across studies first required handling batch effects due to heterogeneity in experimental conditions between labs. The predicted dose-response curves, and corresponding AUCs, from the PPC model do not alleviate batch effects. The model seeks to minimize error for each sample in its specific study and thus recapitulates batch effects in the predicted response curves. In preliminary analyses, we identified that standard global z-scoring of AUCs fails to remove study-specific batch effects (fig. S15). To remove batch effects, we adapted an empirical Bayesian procedure^56,57^ to assign a de-batched z-score to each dose-response curve (Fig. 3a). For each sample, the procedure performs a robust estimation of the null distribution of the AUC histogram, ignoring outliers representing drugs for which the sample is truly resistant or sensitive which would skew the z-score distribution (see Methods). Grouping drugs by mechanism of action, we found global per-study batch effects are reduced using these robust z-scores compared to uncorrected AUCs (fig. S14). The empirical Bayes z-scoring procedure also produced z-scores that tended to be centered around zero, whereas global z-scoring leads to skewed median z-scores for each sample (fig. S17).

Stratifying z-scores by drug target revealed broad differences in drug efficacy across disease types (fig. S19). Proteasome and IAP inhibitors are more cytotoxic than average aggregating across all tissue types. On the other hand, many traditional chemotherapeutic agents (e.g. DNA/RNA synthesis inhibitors like gemcitabine, 5-fluorouracil, and temozolomide) are less efficacious on average, possibly due partially to treatment-induced resistance in some samples. Regardless of the mean rank of a drug class, we observed considerable variance in average efficacy among disease types. For instance, proteasome inhibitors have the highest mean but a range of average z-scores from −2 (SARCNOS) to 3.3 (CEAD), whereas the lowest mean drug z-score across all disease subtypes is DHFR with an average z-score of −1.75. The large variability suggests that, while there is some toxicity bias to drug classes, substantial room remains for sample-specific sensitivities to emerge.

We hypothesized that using the de-batched z-scores as a measure of drug sensitivity may reveal systematic differences across tissue sites. We conducted a differential sensitivity analysis across the PPC dataset by filtering to primary samples and grouping by primary tissue. For each tissue site, we compared the average response to each drug to the average responses on the same drug aggregated across the other 22 annotated tissue sites with viabilities (see Methods). Overall, we found 303 tissue-specific drug sensitivities and resistances (Fig. 3d).

Several of the statistically significant sensitive drug-tissue type associations concord with findings from either molecular assays or clinical studies. VEGFR inhibitor vatalinib demonstrated favorable progression-free survival compared to historical controls in a Phase 2 trial in post-gemcitabine pancreatic adenocarcinoma patients^58^. Cytotoxic purine analog fludarabine has long been studied in head and neck cancer both as systemic monotherapy and in combination, either with radiotherapy or with fludarabine administered intratumorally^59–61^. Recent cell line and organoid screens identified transcriptionally defined sub-populations of pancreatic adenocarcinoma samples susceptible to proteasome inhibitor carfilzomib^62^. Thymidylate synthase inhibitor raltitrexed demonstrates comparable efficacy to 5-fluorouracil-based regimens in colorectal or bowel cancer^63^. EZH2 inhibitors promote cell death via ferroptosis in hepatocellular carcinoma^64^, and we find liver samples are significantly sensitive to EZH2 inhibitor tazemetostat. In combination with immunotherapy, tazemetostat has also shown disease control clinically in colorectal cancer^65^. Purine analog pentostatin shows sensitivity in lymph tissue, has been studied extensively in lymphoid malignancies, and is approved for use in the B-lymphocyte malignancy hairy cell leukemia^66,67^.

In general, drugs targeting the MAPK pathway, which includes MEK or ERK inhibitors, are more efficacious on primary samples from skin tumors than on samples from other sites in the PPC dataset (Fig. 3e). Drugs approved by the FDA for metastatic or unresectable BRAF V600E/K mutant melanoma (Cobimetinib, Trametinib, Binimetinib) were all significantly more effective in skin compared to non-skin samples^68–70^. In contrast, MEK inhibitor selumetinib is only approved for use in a rare, genetically predisposed peripheral nerve sheath tumor and is not significantly more effective in skin compared to non-skin samples^71,72^. Across drugs labeled as targeting MEK or ERK, z-scores representing drugs with measurements are well-mixed with those from fully imputed curves (fig. S18).

## Model imputations predict clinically rational drug responses

To assess the biological and clinical rationale of the model predictions, we conducted an analysis of the BeatAML^13^ study. We focused on BeatAML as it is unique among the PPC datasets for having a large sample size, a broad drug screen panel, and comprehensive genomic profiling for nearly every sample. Using the fully-imputed responses for AML, we identified samples amenable to treatment with agents for biomarker-defined sub-types, despite not explicitly incorporating genomic information in the PPC model (Fig. 3f, top). AML samples with deleterious mutations in *FLT3* and *RAF* genes are associated with improved responses to kinase inhibitors (KIs) with affinity for FLT3 and RAF, respectively, compared to their wild-type counterparts. This is consistent with the fact that FLT3 and RAF inhibitor approved indications are generally restricted to mutant patient populations^73,74^. In contrast, AML samples with deleterious *TP53* mutations were predicted to be less responsive to Nutlin-derived drugs. This underscores the mechanism of action of such drugs which abrogate the interaction between p53 and MDM2 and thereby reduce proteasomal degradation of the former by the latter; this has been demonstrated in wild-type constructs but may be more complicated in mutant *TP53* samples^75^.

Though FLT3 and RAF KIs were generally predicted to be more efficacious in *FLT3*- and *RAF*-mutant samples, respectively, the distributions of predicted z-scores are heterogeneous (Fig. 3f, top). In evaluating which drugs contributed to this phenomenon, we observed that approved or investigational FLT3 drugs (quizartinib, pacritinib, dovitinib) showed greater efficacy than those whose clinical development was discontinued (amuvatinib, tandutinib, CID 16041424, CID 11427553) (Fig. 3f, bottom left)^76–81^. Similarly, RAF KIs adjudicated by Selleckchem to be solely RAF-targeting agents were significantly more effective in *RAF*-mutant samples than those annotated as more broad-spectrum KIs with multiple kinase targets, including RAF (Fig. 3f, bottom right).

## Metastatic samples exhibit systematic differences in absolute and relative drug sensitivities from primary samples

**Figure 4:**
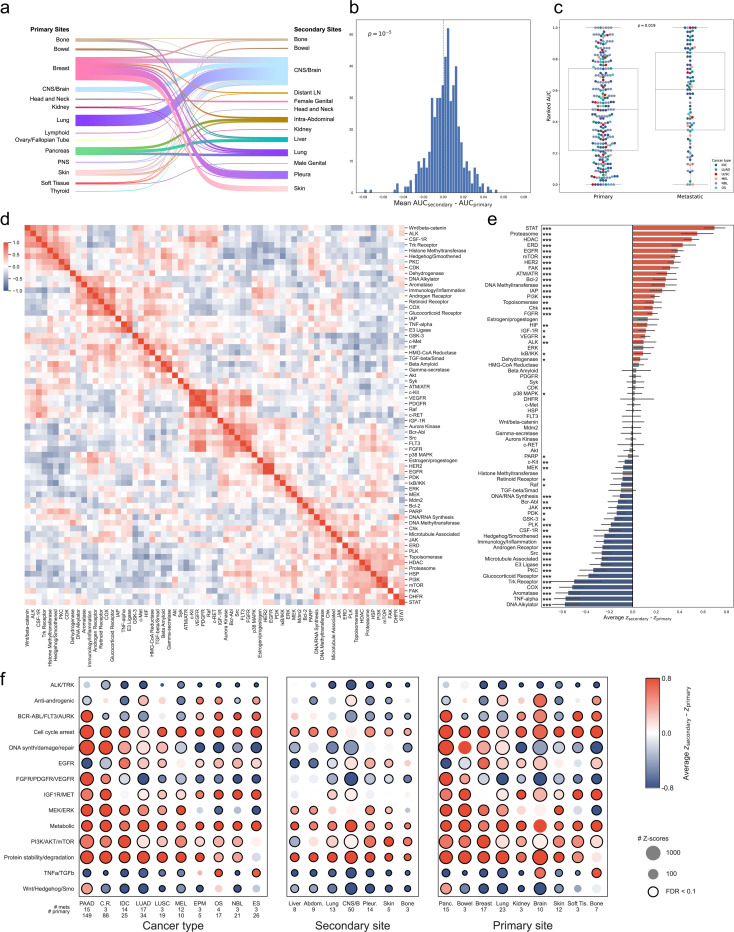
Metastatic samples possess differential drug sensitivities after correcting for intrinsic resistance. (a) Organotropism patterns reflected in the dataset. (b) Distribution of per-drug, per-disease differences in mean AUCs between primary and metastatic samples (left: metastasis more sensitive; right: metastasis more resistant; p-value: one-sided binomial test). (c) Restricting to per-disease standard-of-care drugs and ranking samples by AUC reveals that these drugs are on average less cytotoxic to metastatic ex vivo samples than to primary samples (Methods; *p*-value calculated via two-sided Mann-Whitney U test). (d) Correlations of differences in primary versus metastatic z-scores across disease types. (e) Average difference in z-score between primary and metastatic samples (left: metastasis more resistant; right: metastasis more sensitive; ∗∗∗: *q <* 0.001; ∗∗: *q <* 0.01; ∗: *q <* 0.1, Mann-Whitney U test on BH-corrected *q*-values; error bars: 90th percentile bootstrap intervals; red/blue: *q <* 0.1 and 95th/5th percentile has appropriate sign). (f) Grouped drug signature (vertical axis) z-score differences across top drugs for primary and metastatic samples, grouped by disease (left), primary site (center), and metastatic site (right). Color: mean z-score difference (red/higher: metastatic samples more sensitive; blue/lower: primary samples more sensitive; size: number of samples; solid outlines: significance at FDR=0.1; q-values: two-sided one-sample t-test against 0, with BH correction; C.R.: COADREAD; abbreviations: table S2).

The PPC dataset contains detailed site annotations for both primary and metastatic samples. The metastatic samples are annotated with both the primary site and the tissue site where the metastasis was sampled (Fig. 4a). None of the 26 studies in PPC performed longitudinal sampling of patients to allow for direct primary-to-metastasis comparisons within the same patient. However, the primary disease and site annotations enabled us to perform a population-level assessment of primary and metastatic samples that both originated from the same disease or site.

Across all ex vivo samples of the same disease, metastatic samples were on average more resistant to the same cytotoxic agent (Fig. 4b, *p* = 1 × 10^−5^, one-sided binomial test). Metastatic samples remained more resistant on average when analysis was restricted to drugs designated as disease standard-of-care in the NCCN Guidelines (Fig. 4c, *p* = 0.019, two-sided Mann-Whitney U test)^82^. Drug resistance in metastatic disease is a well-described property of metastatic cancer^83^.

We reasoned that using the PPC z-scores instead of raw AUCs would remove the intrinsic resistance bias in metastatic samples and reveal patterns in relative drug sensitivity. Samples were stratified by disease type and primary status (primary or metastasis). In total, there were 20 disease groups with at least 1 sample in each stratum. Within each disease group, z-scores were averaged and the difference between the metastatic and primary average was used as a measure of relative increase in sensitivity for the metastatic samples (see Methods).

Hierarchical clustering on the z-score differences correlation matrix revealed functional subgroups of drug targets that see similar difference-in-response, across diseases (Fig. 4d). Targets upstream and downstream of one another in the same pathway, including PI3K/mTOR and MEK/ERK, are shown to cluster together. Several tyrosine kinase targets cluster closely in the center of the heatmap, including c-Kit, VEGFR, PDGFR, IGF-1R, Bcr-Abl, Src, and FLT3. We attributed this to the relative promiscuity of kinase inhibitors that leads single agents to target multiple kinases. A number of cell division-related targets cluster on the bottom right of the heatmap with emergent sub-clusters, including DNA damage repair and synthesis (PARP, DNA/RNA synthesis) and mitosis-related targets (Chk, microtubule associated, PLK, and topoisomerase).

Across diseases with both primary and metastatic ex vivo samples, comparative efficacy of labeled drug targets varies (Fig. 4e). Specifically, drugs labeled as targeting STAT, ATM/ATR, HER2, EGFR, FAK, HIF, and HDAC are found to have higher relative cytotoxicity on average in metastatic samples compared to primary samples across diseases. Conversely, drugs labeled as targeting TNF-*α*, DNA alkylators, and COX are found to be less relatively cytotoxic on average in metastatic samples compared to primary samples. Drugs labeled as targeting STAT were found to differ from those targeting JAK in comparative primary-metastatic sensitivity. JAK-targeting drugs were more effective on primary samples, while STAT-targeting drugs were more effective on metastatic samples. Differential phosphorylation of STAT3 has been previously observed between matched primary and metastatic lung cancer samples^84^. Targeting STAT3 activation has been observed to inhibit both tumor growth and metastasis in vitro and in vivo^85^, while targeting JAK alone has been observed to be insufficient in blocking growth of some late-stage ovarian cancer models^86^.

## Drug sensitivity differs based on metastatic sample site for some drug classes

Tissue- and disease-specific patterns of organotropism have been shown to be statistically associated with potential metastatic driver mutations^43^. Corresponding patterns in functional responses to drug treatments have not been investigated at scale. We stratified samples by primary and metastatic tissue sites, as well as disease subtype. Diseases and sites with fewer than three metastatic samples were excluded. Drug response z-scores were aggregated into higher level mechanistic groups based on the correlation structure among mechanism of action annotations (Fig. 4d). Differences between average z-scores were calculated and tested for significance using a two-sided t-test (see Methods).

With some exceptions, average z-score differences between metastatic and primary samples are generally directionally consistent across cancer types, metastatic sites, and primary sites for the top drug classes (Fig. 4f). TNFa/TGFb targeting therapies are significantly more effective in metastatic compared to primary samples for OS and ES and for samples with primary sites in brain and bone, in line with preclinical reports regarding the importance of TGFb in local growth and metastatic progression in these tumor types^87–89^. Wnt/Hedgehog/Smo targeting therapies are significantly more effective in metastatic compared to primary samples for PAAD and samples whose primary site is the pancreas, possibly reflecting the importance of non-canonical Wnt signaling in pancreatic metastasis through effects of epithelial-to-mesenchymal (EMT) transition and cancer stemness^90^. Drugs targeting DNA synthesis, damage, and repair exhibited the greatest degree of variation across cancer types and sites. Such drugs exhibited significantly increased efficacy in metastatic compared to primary samples for PAAD, COADREAD, IDC, LUSC, and LUAD and significantly increased efficacy in primary compared to metastatic samples for MEL, EPM, OS, NBL, and ES. Overall, sample sizes for the subtype- and site-specific analyses were limited: only two site categories had more than 20 met samples and most cancer subtype categories had fewer than 15 mets. We therefore caution that these results in particular are preliminary and warrant expanded data collection to draw robust conclusions.

## Cell line drug responses are systematically different from ex vivo drug responses

The emergence of organoids and other ex vivo models has led to a shift in the drug screening field away from traditional immortalized cell lines and towards ex vivo models, in the expectation that results will better replicate patient responses to therapy^91^. Though one study in the PPC dataset^11^ performed limited comparisons to traditional 2D cell lines, no study to-date has had the breadth of samples and screen results to perform pan-cancer analyses comparing cell line and ex vivo drug responses. We sought to address this gap by integrating existing cell line drug screen atlases^24,92–101^ into the PPC dataset. We hypothesized that comparing drug responses between disease-matched model subpopulations would reveal distinct, recurrent differences between the two modalities.

Cell line drug responses were integrated into the PPC dataset using the same preprocessing pipeline previously described for ex vivo samples (see Methods). Primary diagnosis and metastasis status were obtained from Cellosaurus^102^ where available. In total, we integrated 2,790 cell lines, 21,005 drugs, and 50.3M viability measurements. We refit the PPC dose-response model jointly to all cell line and ex vivo samples. The same contrastive penalties were used in the joint model as in the model trained purely on ex vivo samples. Drug responses were normalized using the same robust z-scoring procedure used for ex vivo samples.

**Figure 5:**
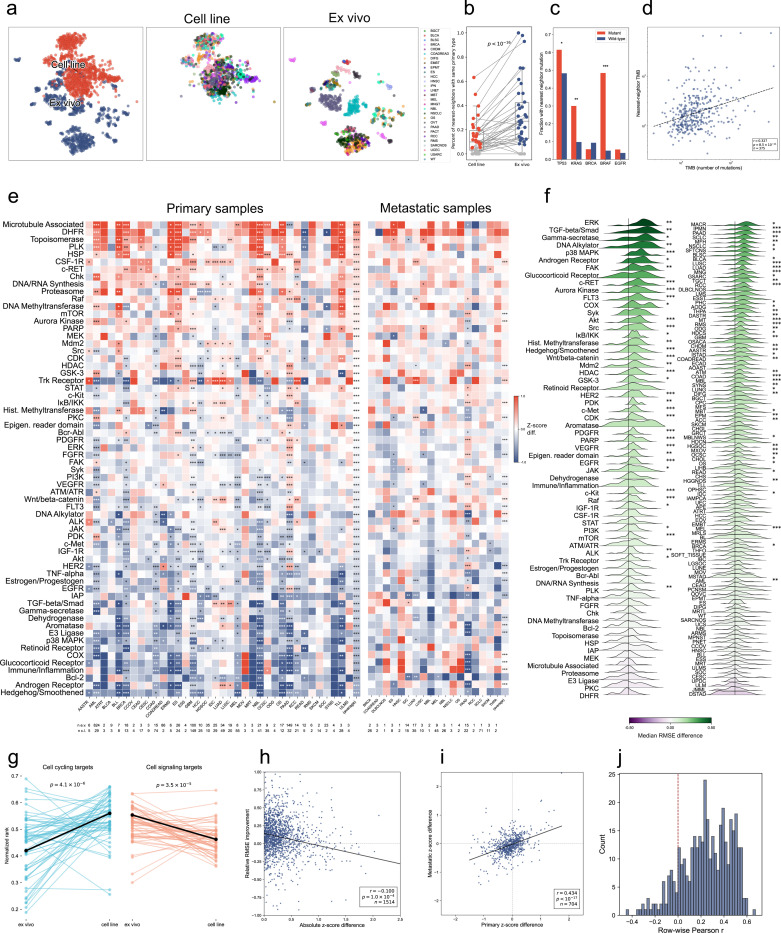
Cell line and ex vivo constructs differ systematically in drug response. (a) UMAP embeddings for cell line and ex vivo sample embeddings from the dose-response model trained jointly on all data (left). Ex vivo samples (right) cluster by primary cancer type, whereas cell lines (middle) do not. (b) Percentage of samples, grouped by disease, for which the aggregated 5 nearest-neighbors (in sample embedding space) is of the same disease. In general, ex vivo embeddings cluster more by disease than cell line embeddings; gray points: *q >* 0.1 after BH correction on a two-sided binomial test; global p-value via two-sided Fisher’s exact test (boxes: quartiles 1 and 3; line: median; whiskers: 1.5 IQR). (c) Proportion of cell line sample embeddings (with genomics) whose nearest-embedding neighbor has at least one mutation for that gene (*: *q <* 0.1; ***: *q <* 0.001; q-values: two-sided Fisher’s exact test with BH correction). (d) Tumor mutational burden (TMB) of samples nearest neighbor versus sample TMB, for cell line samples with genomics data ( r: Pearson correlation; p-value: Pearson correlation test). (e) (Left) Relative efficacy differences stratified by disease types (horizontal axis) and annotated drug targets (vertical axis) for primary samples (left) and metastatic samples (right); red/higher: cell lines are more sensitive, blue/lower: ex vivo samples are more sensitive; *q*-values derived from BH-corrected permutation test (Methods); disease-average p-values calculated via Fisher’s method (∗∗∗: *q <* 0.001; ∗∗: *q <* 0.01; ∗: *q <* 0.1; BH corrected). (f) Distribution of per-disease relative change in model cross-validation RMSE when cell line data is added to model training set (Methods), stratified by drug target (left) and disease (right) (∗∗∗: *p <* 0.001; ∗∗: *p <* 0.01; ∗: *p <* 0.05); purple/lower: adding cell lines samples decreases performance (higher RMSE); green/higher: the addition increases performance. (g) Per-disease comparison of normalized rankings of cell-cycling and cell-signaling targeting drugs across model construct type. Higher rankings represent greater comparative drug efficacy on samples (p-values: two-tailed paired t-test; target groupings: table S4). (h) Relative RMSE improvement (as aggregated in (f) above) vs absolute z-score difference (as aggregated in (e) above) per target/disease pair (*r*: Pearson correlation; *p*: Pearson correlation test). (i) Primary versus metastatic z-score difference scores for matched diseases at least two samples of each group shows moderate concordance between primary and metastatic samples (*r*: Pearson correlation; *p*: Pearson correlation test). (j) Histogram of Pearson correlations per distinct pair of drugs across mean efficaciousness z-scores per-disease (fig. S25).

We first inspected the ability of the PPC dose-response model to learn integrated representations of the cell line samples. We found that cell line samples did not integrate with ex vivo samples when projected using UMAP (Fig. 5a). Classifying samples by disease type, we found that the sample embeddings on the jointly trained dose-response model qualitatively exhibit substantially more clustering by disease type than the cell line models (Fig. 5a). Comparing sample embeddings by nearest neighbors in Euclidean space confirmed that ex vivo models are significantly more likely than cell line samples to cluster with samples from the same disease type (Fig. 5b; Methods). We reasoned that immortalized cell lines may have lost many of the properties of their cell of origin and instead become driven by their molecular alterations due to selection for growth in culture. We found that cell line embeddings with TP53, KRAS, and BRAF mutations are significantly more like to cluster near other sample embeddings with the same mutation than wild type sample embeddings are (Fig. 5c), suggesting that mutation status of these genes is important in characterizing sample drug response. Similarly, cell line embeddings with high tumor mutational burden (TMB), as measured by the absolute number of mutations found, are significantly more likely to have neighbors with higher TMB (Fig. 5d). Using bootstrap data resampling, we observed that this trend was robust with an estimated 95% confidence interval of *r* = [0.225, 0.425] (fig. S22).

To assess whether cell lines have systematically different drug responses, we next compared z-scores between disease-matched model subpopulations. Cell line and ex vivo models were stratified by primary disease annotation and primary or metastatic sample status. Drug response z-scores were grouped by drug class and averaged to get the typical cell line and ex vivo relative efficacy scores for each class within a disease subgroup. The difference between the average cell line and ex vivo z-score were then calculated as a measure of deviation between modalities; results were tested for statistical significance via a nonparametric permutation test (see Methods).

Remarkably, we observed systematic deviations between cell line and ex vivo z-scores across disease types when grouping by drug class (Fig. 5e). Among 36 disease categories and 64 drug classes, we found 700 significant differences between disease-matched subpopulations (621 primary, 79 metastatic). Of these, 234 (33%) were more effective in cell lines and 466 (67%) were more effective in ex vivo samples. Across disease types, 63 of 64 drug classes were significantly different in primary samples and 24 classes were significantly different in metastatic samples, though only 5 exhibited differences greater than 0.5 z-score standard deviations (on primary samples, microtubule-associated, DHFR, and Hedgehog/Smoothened; on metastatic samples, DHFR and Bcl-2). Disease-matched differences were concordant between primary and metastatic indications (Fig. 5i,j), suggesting differences were robust to tumor stage.

Drugs targeting rapidly cycling cells (e.g., microtubule targeting drugs, DHFR inhibitors, topoisomerase inhibitors) were found to be significantly more effective on cell lines compared to ex vivo samples in 20/36 (56%) disease types. These include broad-spectrum chemotherapeutics like 5-FU, irinotecan, and oxaliplatin, which have previously been shown in limited experiments to be more resistant under 3D culture than in 2D monolayers^103^. Similarly, the chemotherapeutic Paclitaxel, which disrupts microtubule dynamics, has been found to be non-cytotoxic in some ex vivo models^104,105^ while inducing cell death in immortalized cell lines^106,107^. Aggregating across diseases, cell lines are significantly more sensitive to cell-cycling drugs compared to ex vivo samples and less comparatively sensitive to drugs targeting a broad class of intercellular signaling pathways (Fig. 5g). We observed a moderate to strong correlation (Pearson *r* = 0.434; *p <* 10^−17^) between the primary and metastatic sample scores (Fig. 5i), suggesting the differences are robust to the stage of the underlying tumor, though more detailed clinical annotations would be needed to assess this systematically.

Drug classes that were relatively more effective on ex vivo samples were enriched for autocrine and paracrine signaling-related pathways. These include receptor tyrosine kinases (e.g., EGFR, IGF1R, FGFR, VEGFR, PDGFR), migration and remodeling signaling (Wnt/beta-catenin, TGF*β*/Smad), and enzyme complexes (e.g., gamma-secretase, COX). Grouping by cell cycling or cell signaling related drugs showed significant trends for both polarized findings (fig. S20).

Among the less consistently polarized differences, TRK receptor drugs displayed outsized differences for either ex vivo or cell lines, depending on the specific disease subtype. Given the rarity of NTRK fusions, which occur at frequencies below 1% in common cancers like lung and colorectal cancer^108^, the relatively higher magnitude of differences across disease types for TRK receptor-targeting drugs observed in Fig. 5c is not explainable by on-target fusion activity. This may be due in part to the promiscuity of first-generation TRK inhibitors like entrectinib, which have been observed to inhibit multiple off-target kinases like ROS1 and ALK^109,110^.

We considered that differences in media conditions may have led to confounding that accounts for these results. To test this, we extracted all additives used in the media of the ex vivo and cell line studies (see Methods); this yielded 42 unique additives used in at least three studies. We one-hot encoded the presence or absence of each additive and whether the sample was a cell line or ex vivo sample, then ran two-covariate regressions for each drug target category and additive. Results showed that some additives do explain part of the variance in scores, but none affect the directionality or general systematic bias attributable to the differences between ex vivo and cell lines (fig. S23(a), fig. S24). We further tested this by running *ℓ*_1_-regularized multiple regressions controlling for all media additives, study ID, and ex vivo status. The high dimensional, collinear nature of the features led to high variation in the predicted coefficients, but we nonetheless observed the same trend as in the marginal and two-covariate analyses (fig. S23(b)).

## Integrating cell line drug screens improves ex vivo drug prediction accuracy

Despite the systematic differences between cell line and ex vivo drug responses, we found integration of cell lines still benefited predictive performance on ex vivo samples. Training the PPC model on cell line data combined with ex vivo data, compared with training on the ex vivo dataset alone, significantly improved empirical held-out prediction performance on ex vivo data for 38/64 (59%) drug classes and did not significantly reduce performance for any class (Fig. 5f, left). Similarly, model predictive performance improved significantly across 48/109 (44%) disease subtypes and did not significantly reduce performance for any class (Fig. 5f, right).

We observed a weak association between the size of cell line response discrepancy and integration improvement across drug classes (Fig. 5h). Leveraging bootstrap resampling, we observed that this trend was robust with an estimated 90% confidence interval of *r* = [−0.163, −0.079] (fig. S21). We further observed similar behavior across inter-drug correlation among both cell line and ex vivo samples (Fig. 5j). Drugs more often than not exhibit positively correlated behavior when comparing individual rows of the drug correlation matrices between ex vivo and cell line samples (fig. S25). These results suggest that, while cell lines are biased relative to ex vivo viabilities, the similar viability correlation structure appears to be sufficient to improve predictive ex vivo performance of the PPC model.

In aggregate, the results suggest that cell line drug responses should be interpreted and utilized with caution. Directly translating cell line responses to ex vivo responses is likely to be error prone and biased. However, when used as a data augmentation tool to better learn relational structure between drug responses, they consistently boosted the predictive performance of the PPC model. We therefore anticipate that future foundation models for ex vivo drug response would likely benefit from incorporating cell line drug responses.

## A Foundation Model for Functional Precision Oncology

Clinical application of functional precision oncology is often limited by low tissue availability and the need for biologically rational explanations of drug response^23^. Insufficient material for large scale ex vivo screens constrains the size and diversity of the drug library, reducing the range of possible therapies for patients. Molecular profiling also faces numerous clinical challenges^111^, making it difficult to generate orthogonal genomic and transcriptomic data needed to support molecular tumor board recommendations for drug screen hits. While notable successes exist, such as the INFORM trial^20^, it remains prohibitively challenging to scale simultaneous functional and molecular profiling across diverse cancer indications, patient cohorts, and disease stages.

We reasoned that a computational platform based on the PPC dataset could ameliorate these challenges. We sought to leverage the PPC database to build a foundation model for ex vivo drug screens that enables efficient and accurate “few-shot” prediction for new studies, using only a small number of drug response observations to predict a much larger range of doses and compounds. Unlike existing cell line dose-response models that assume comprehensive genomic and transcriptomic profiling^49,52,112–116^, we sought to build a model capable of predicting full ex vivo dose-response curves and molecular profiles entirely based on a small drug screen over a handful of concentrations. We also required that the model worked with novel patient samples, preventing the adaptation of cell line foundation models that rely on large language models to generate literature-informed embeddings about previously-studied samples^117^.

**Figure 6:**
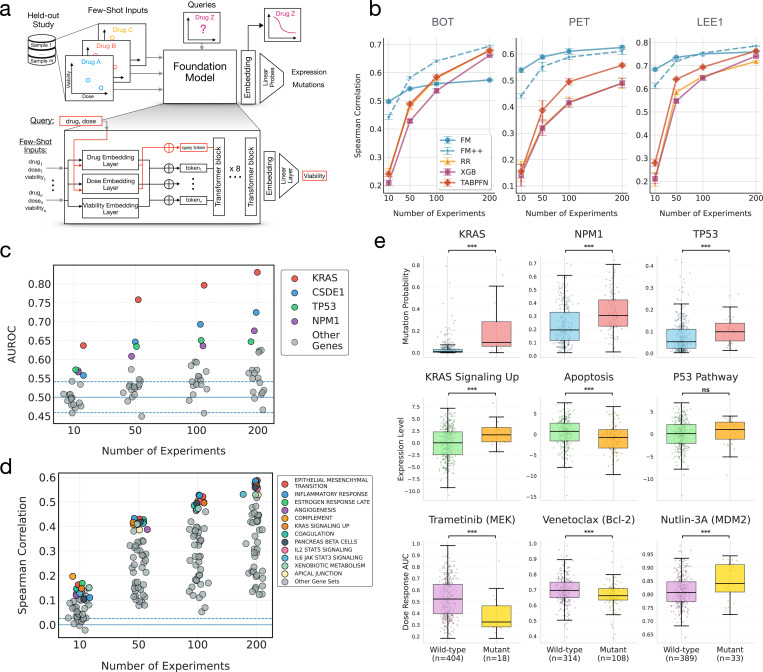
Foundation model evaluation and multi-omics analysis. **(a)** Overview of the experimental setup. The foundation model (FM) is trained on PPC with one study held out at a time, enabling few-shot dose–response inference for unseen samples. **(b)** Few-shot drug response performance. Spearman Correlation for dose response prediction as a function of the number of experiments, i.e. the number of (drug, dose, viability) observations $\left(n_{\text{few-shots}}\in \{10,50,100,200\}\right)$. The three held-out studies are BOT^13,28^, PET^20^ and LEE1^9^. Error bars show two standard deviations over five random repetitions. The Foundation Model (FM) and its finetuned version (FM++) is compared against TabPFN, XGBoost (XGB), and Ridge regression (RR) baselines. **(c–e)** Multi-Omics analysis conducted on the Beat AML cohort (BOT). **(c)** Prediction of gene mutation status from sample embeddings. Linear probes are trained to predict binary mutation labels using embeddings derived from the FM. Shown are mean AUROCs across five cross-validation folds and five random few-shot selections, highlighting the 4 most accurately predicted genes. **(d)** Prediction of pathway-level gene expression. Ridge regression models predict aggregated Hallmark pathway activity scores from FM embeddings. Results are averaged over five cross-validation folds and five random few-shot selections; the 12 best-predicted pathways are displayed. **(e)** Multi-omics consistency analysis across mutation, pathway activity, and drug response. Rows show mutation logits, pathway scores, and predicted drug AUCs for $n_{\text{few-shots}}=100$. Boxplots compare wild-type and mutant groups. One-sided Mann–Whitney U tests were repeated 5 times; p-values were combined using Fisher’s method. Significance: * p < 0.05, ** p < 0.01, *** p < 0.001.

The PPC foundation model uses a transformer-based architecture inspired by BERT^118^ and TabPFN^119^ to perform few-shot multimodal prediction from a small number of drug screen results on a sample (Fig. 6a). The model is made of 8 transformer blocks with hidden layer size of 384, intermediate hidden size of 512 and 8 attention heads in each transformer. The foundation model directly takes as input a small labeled input set and an unlabeled query set, and outputs predictions for the query samples. The input set consists of a small number of labeled triplets (drug, dose, viability) measured on a new biological sample, while the query set contains unlabeled pairs (drug′_,_ dose′) for which the model predicts viability. This setup allows the model to infer viability responses for unseen drug–dose combinations based solely on a few measured examples from the same biological sample. Both input and query sets are encoded using sequence of tokens for the drug, dose and viability. The drug tokens are given by an embedding layer, which is a dictionary retuning a vector of size 128 for each drug. For the dose and viability embedding, we also use vectors of size 128. To encode the continuity of these values, the embedding is given by a linear combination of Fourier features with learnable parameters. Finally, the three embeddings corresponding to the drug, dose, and viability are concatenated to form a 384-dimensional embedded token for each (drug, dose, viability) triplet in the input sequence. For the query sequence, a placeholder token is used for the unknown viability value, resulting in query tokens with the same 384-dimensional representation. The input and query sequences are then processed by eight Transformer blocks, which perform cross-attention between the two sequences. The resulting query token representations are passed through a linear prediction head to estimate viability.

## The PPC foundation model reduces the number of experiments needed to accurately predict drug responses

To assess few-shot performance, we evaluated how accurately the model can recover the dose–response for a new sample in a held-out study when only a few observations are available. We conducted independent benchmark experiments using the three PPC studies (BOT, PET, LEE1). The three studies chosen represent the largest drug libraries, broadest range of concentrations, and the largest ex vivo sample sizes, enabling us to test a range of potential down-scaled experimental designs. Each benchmark experiment used a leave-one-study-out design, holding out one study at a time while training on the rest of the PPC dataset, including cell lines. The held-out study was then treated as a new domain, where only a few-shot subset of labeled observations is available. These few-shot examples were used as input to the foundation model to produce dose–response curve predictions and sample embeddings.

We evaluated the viability predictions and embeddings to assess the capacity of the model to generalize to unseen studies and efficiently adapt to new drug screen experiments. Performance on viability predictions was compared against linear (ridge regression), machine learning (XGBoost^120^), and generalist foundation models (TabPFN^119^). For the PPC foundation model, we considered two use cases: (i) a static model (FM) that makes predictions purely using attention over the inputs, and (ii) a fine-tuned model (FM++) that updates the model weights to the new data before making predictions.

The PPC foundation model consistently outperformed all baseline models across the three held-out studies (Fig. 6b). Baseline models approached FM performance when the number of experiments was large (≥ 200). When data was more limited, as might the case with low tissue availability, the PPC foundation model correlation was 100–400% higher than the baselines. Fine-tuning (FM++) slightly underperformed FM when only 10 experiments are available but achieved similar results after 50 experiments for two studies. For one of the three studies, the fine-tuned model provided a clear improvement, suggesting that this study is either more challenging or less similar to the other PPC studies. We therefore generally recommend fine-tuning when possible to ensure the most robust performance. When access to GPUs are unavailable, the static foundation model still represents a high performing model available for rapid prediction.

## Foundation model probes provide rational genomic and transcriptomic inter-pretability for drug predictions

A key benefit of foundation model architectures is their ability to generate embeddings that serve as general purpose covariates for predicting a wide range of related tasks for which the model was not originally trained^121^. We reasoned that the PPC foundation model embeddings may enable accurate imputation of genomic and transcriptomic profiles, particularly those driving phenotypic response to drugs. If accurate, imputing molecular profiles would enable a layer of biological interpretability to the foundation model predictions. Imputed profiles could also serve as biological rationale for molecular tumor boards in the setting where comprehensive multimodal profiling is infeasible.

To evaluate the embeddings, we used the Beat AML cohort (study BOT), which gathered both whole exome sequencing and bulk RNA sequencing on matched patient tissues, in addition to performing ex vivo drug screens. For each sample, the foundation model produced embeddings derived from dose–response predictions using $n_{\text{few-shots}}\in \{10,50,100,200\}$. We then trained simple linear probes on these embeddings to assess how well they captured underlying molecular features. Binary mutation status was predicted using logistic regression. Gene expression data was normalized and log-transformed, then aggregated at the pathway level using the Hallmark gene sets^122^; the first principal component across member genes was used as the pathway activity score. Performance on each modality was evaluated using 5-fold cross-validation.

Prediction accuracy varied across targets but was particularly high for targets directly related to drug response and resistance (Fig. 6c-d). Mutations in key oncogenic and resistance drivers, namely KRAS, TP53, CSDE1 and NPM1 were among the most accurately inferred (Fig. 6c) with AUROC 0.65–0.83 with 200 observations. Drugs in the Beat AML library, like idasanutlin and trametinib, target upstream or downstream targets of these genes. In the case of KRAS, this structure is further reflected in the embedding space (fig. S26), where mutant and wild-type samples form distinct clusters. Pathway prediction showed variability but consistently improved in accuracy as the number of observations increased (Fig. 6d). Pathway predictions were also most accurate when they related to disease severity (e.g. epithelial mesenchymal transition) or were connected to drug targets in the panel (e.g. KRAS signaling up).

We further analyzed the molecular imputations for cross-modality sensitivity. While each modality may be well predicted in isolation, it is possible that the two predictions were discordant when considered jointly, or in context of the predicted drug response. For each selected gene–drug pair, we jointly examine the mutation probability inferred from the foundation model embeddings, the corresponding pathway activity score, and the predicted drug sensitivity (AUC). The results show coherent molecular patterns across omics layers and drug response predictions (Fig. 6e). In KRAS-mutated samples, the model assigns higher mutation probabilities and elevated scores for the “KRAS signaling up” pathway, while predicting increased sensitivity to Trametinib–a MEK inhibitor acting downstream of KRAS. Similarly, NPM1-mutated samples show higher predicted mutation probabilities and reduced activity of the apoptosis pathway, with Venetoclax (a BCL-2 inhibitor) predicted as more effective in this subgroup. For TP53, the model correctly associates higher mutation probabilities with reduced sensitivity to Nutlin-3A, an MDM2 inhibitor whose efficacy depends on intact p53 signaling. Altogether, these results indicate that the foundation model embeddings capture biologically consistent associations between mutations, transcriptional programs, and pharmacological responses.

## Discussion

This work has established and analyzed a pan-cancer ex vivo drug screen atlas. In doing so, it addresses outstanding questions in translational cancer research, including (i) is multi-study drug response integration valuable in the presence of batch effects and differing experimental conditions, (ii) how do metastatic and primary samples differ in drug response, and (iii) in which ways do cell line drug responses differ from their ex vivo counterparts. The comparative analyses and modeling results provide guidance for future study designs with respect to data integration strategies and power calculations. This work also established the pan-preclinical (PPC) project and foundation model, computational resources for the cancer biology and functional precision oncology communities.

Several key limitations underlie the PPC dataset and represent important challenges in the development of next-generation methods for predicting ex vivo drug response. First, the degree of matched omics varied widely between studies, making the overall PPC dataset highly sparse in side information. This sparsity led us to design a flexible tensor factorization method rather than building on existing machine learning methods for drug response prediction, which typically rely on matched genomics and/or transcriptomics^49–52^. More consistently matched omics would enable a deeper dive into mechanistic drivers that could explain, for instance, many of the differences we found between cell lines and ex vivo drug response. Similarly, the PPC samples were taken as-is from studies with different selection criteria. Some studies chose to obtain only treatment naive samples, whereas others took heavily pre-treated patients. As we were unable to retrieve complete treatment history for each sample in the dataset, it is difficult in principle to disentangle the inter-related effects of highly progressed cancer, metastatic cancer, and prior anti-neoplastic treatment on drug responses observed across samples. There are also many careful considerations one must make when interpreting drug screen data based on cell viability;^123^ for example, bulk metabolic assays cannot readily distinguish between proliferative arrest and subpopulation-level cell death, among other concerns. Gathering additional time points to estimate growth rate inhibition^124^ or directly tracking single cells or organoids in real time^125^ would alleviate these confounding issues.

Ex vivo screening platforms are advancing at a rapid rate, with newer assays enabling more faithful modeling of the tumor microenvironment (TME). We anticipate that emerging ex vivo assays that incorporate TME characteristics (e.g. through co-culture^126^, microfluidics^127,128^, or explant culture^129,130^) represent the next generation of ex vivo platforms. Tumor cell interactions with other cells in the TME, such as cancer-associated fibroblasts^131,132^, cytotoxic T-cells^133^, regulatory T-cells^134^, and M1/M2-like macrophages^135^, are known to mediate tumor proliferation^136^ and response to some therapies^137^. These platforms could also enable in vitro testing of drugs targeting the TME and its constituent cell types such as PD-1/PD-L1/CTLA-4 blockade for exhausted T-cells and emerging therapies like anti-CSF1/CSF1R therapy for tumor associated macrophages^138^. The PPC project is built flexibly and with an eye towards incorporating a wide array of studies and measurements. As these technologies mature, we anticipate incorporating both TME assays and therapies into the PPC dataset to improve its translational value and reveal more insights into tumor drug resistance and response. The PPC project is therefore poised to continue to push the boundaries of translational cancer biology and predictive oncology.

## Supplementary Material

Supplement 1

Materials and Methods

Figs. S1 to S26

Tables S1 to S6

Algorithm S1

## Data Availability

Unless excepted below, the data that support the findings of this study will be made freely available. The data from Mayoh et al.^29^ is available upon request from the authors of that study. Restrictions may apply to the availability of these data, which were used with agreement for this study.
