# Supplementary material for: A Pan-Cancer Ex Vivo Drug Screen Atlas for Functional Precision Oncology
Source: bioRxiv. 2026 Feb 17:2026.02.14.705918. Preprint. [Version 1] doi: 10.64898/2026.02.14.705918 (PMC12934811; doi:10.64898/2026.02.14.705918)
Supplement: Supplement 1 [file NIHPP2026.02.14.705918v1-supplement-1.pdf]

bioRxiv preprint doi: https://doi.org/10.64898/2026.02.14.705918; this version posted February 17, 2026. The copyright holder for this preprint (which was not certified by peer review) is the author/funder, who has granted bioRxiv a license to display the preprint in perpetuity. It is made available under aCC-BY 4.0 International license.

Supplementary Materials for A Pan-Cancer Ex Vivo Drug Screen Atlas for Functional Precision Oncology

Karl Pichotta[1†], Jessica B. White[1,2†], Jeffrey F. Quinn[1†],
Anneliese Markus[3], Christopher Tosh[1], Antoine De Mathelin[1],
Erin Coyne[4], Feiyang Huang[1,2], Wesley Tansey[1*]
*Corresponding author. Email: tanseyw@mskcc.org
†These authors contributed equally to this work.

This PDF file includes:

Materials and Methods
Figures S1 to S26
Tables S1 to S6
Algorithm S1

bioRxiv preprint doi: https://doi.org/10.64898/2026.02.14.705918; this version posted February 17, 2026. The copyright holder for this preprint (which was not certified by peer review) is the author/funder, who has granted bioRxiv a license to display the preprint in perpetuity. It is made available under aCC-BY 4.0 International license.

Materials and Methods

Dose-response harmonization

We defined a dose-response observation as a single experiment where a given cancer model was exposed to one or more drugs at defined concentrations. A single dose-response observation must be labeled with a harmonized sample identifier, and one or more harmonized drug identifiers. Drug concentration across all studies was converted to micromolar units (μM). All dose-response observations must have a negative control where the same model was exposed to no drug, and optionally can have a positive control where the model was exposed to a compound at a known lethal concentration. We calculate viability as

$$\frac{y - c_p}{c_n - c_p}$$

where y is the raw readout of the experiment (e.g. fluorescence value, culture area, etc) and c_n and c_p are the raw readouts of the negative and positive controls, respectively. In the case where the positive control is not included in the study it takes the value 0 in this formula. In cases where y was observed multiple times within the same technical replicate (e.g. if the same model is exposed to the same concentration of drug in multiple wells on the same plate) the mean of those observations is used. The raw readout units vary between studies, but because the viability metric is always normalized by control values measured in the same units it is comparable across studies. Viability is a positive real number, with a value greater than 1.0 indicating the model grew more under drug treatment than under the negative control.

In some source studies raw experiment readouts were not reported and viability metrics were reported directly; in these cases we confirmed that the reported viability metric will be comparable to metrics we calculated directly using the above formula.

Cancer primary annotation

Cancer diagnoses were harmonized to the OncoTree nomenclature[42] by matching sample diagnoses to NCIT records' synonym/abbreviation fields[139] in addition to Cellosaurus records' disease field[102]. The combined list of NCIT synonyms in addition to Cellosaurus diseases were then iteratively queried on the OncoTree API[42], until the most specific OncoTree code that was a direct match was identified. For example, a sample from Lee et al.[9] diagnosed as "Myxoid liposarcoma, FNCLCC grade 1/3" simplified to "Myxoid liposarcoma" returns no direct OncoTree code matches[42]. However, the combined results from querying NCI ThesaurusSioutos et al.[139] and CellosaurusBairoch[102] yield ["MLPS", "Myxoid Liposarcoma", "Myxoid/Round Cell Liposarcoma", "Myxoliposarcoma"], of which the third entry, "Myxoid/Round Cell Liposarcoma", maps directly to the OncoTree code of "MRLS". Difficulties were posed by the free text nature of most cancer diagnoses data collection and the lack of standardized acronyms. As a result, when no direct matches could be found by this method, manual adjudication was required to select the most accurate OncoTree code. In a few cases of extremely rare disease with no appropriate match, the OncoTree code was marked as null or the primary tissue type.

bioRxiv preprint doi: https://doi.org/10.64898/2026.02.14.705918; this version posted February 17, 2026. The copyright holder for this preprint (which was not certified by peer review) is the author/funder, who has granted bioRxiv a license to display the preprint in perpetuity. It is made available under aCC-BY 4.0 International license.

Cancer metastasis annotation

Cancer metastasis sites were harmonized under the MSK-MET classification system[43]. Only distant metastases, that is, neoplasms sufficient to categorize as "M" within the TNM guidelines set by the AJCC[140] for each cancer type, were considered. For rare cancers and central nervous system cancers (e.g. meningiomas and medulloblastomas) for which TNM staging was nonexistent, the original study's judgment as to metastasis status was used. Due to the non-standardized nature of metastasis description, manual adjudication was utilized when there was not a direct match to MSK-MET classes in order to select the most accurate metastasis site label.

Media additive annotation

For all but three studies, detailed media additives were described or referenced in the source publications [15,28,98]. We noted the presence or absence of specific media additives, including growth factors, small molecules, enzyme degraders, amino acids, antibiotics, antimycotics, antioxidants, proteins, vitamins, serums, and fatty acids. For studies that referenced protocols from previous work rather than detailing their own methods, additives were assumed to be identical to the cited publications. We did not include basal media, which were generally Dulbecco's Modified Eagle's Medium/Ham's F-12 (DMEM/F12) or Roswell Park Memorial Institute (RPMI) media. Additives used in each study were assumed to be applied to all samples, though sample-specific differences were noted in some studies. A total of 50 media additives were documented, 42 of which were used in three or more studies and included in our analyses. Full names for additive abbreviations and acronyms are available in table S6.

Genomics variant harmonization and annotation

Genomic variants were reported several ways in the source publications; standards such as HGVS Variant Nomenclature and VCF files as well as a variety of *ad hoc* nomenclatures and tabular formats were observed [141].

We developed a data model to encapsulate the heterogeneous variant information reported by our source publications and created a software framework to extract and transform all variants into this model. Once extracted, variants were harmonized and enriched using GenomeNexus[142]. GenomeNexus is able to harmonize variants from different reference sequence modalities; for example a variant reported using genomic coordinates like 7:g.55249071C>T can be matched to variants reported using amino acid changes like EGFRp.T790M. We further used GenomeNexus to enrich our annotated variants with information such as transcript consequences, SIFT and Polyphen scores, and clinical information from OncoKB [143–145]. Frameshift and stop-gain mutations along with missense mutations with deleterious SIFT predictions were annotated as deleterious. Once annotated, all variants were lifted over to the GRCh38 reference genome to facilitate cross-publication analysis [146].

Expression harmonization

Upon review of the relevant publications, we noted that 8 studies performed RNA-seq experiments as part of their protocols and made the resulting raw or normalized count matrices readily publicly accessible[1,4,9,11,30,31,37,41]. Upon request, two authors provided raw FASTQ files from their RNA-seq

bioRxiv preprint doi: https://doi.org/10.64898/2026.02.14.705918; this version posted February 17, 2026. The copyright holder for this preprint (which was not certified by peer review) is the author/funder, who has granted bioRxiv a license to display the preprint in perpetuity. It is made available under aCC-BY 4.0 International license.

experiments for analysis[7,34]. One study performed concurrent microarray profiling and made the corresponding quantile-normalized results readily publicly accessible[38].

For the datasets that required FASTQ file processing, we created a Nextflow pipeline available in a standalone repository[1] to enable reproducible quality control, adapter trimming, sequence alignment, read quantification, and initial gene identifier harmonization[147]. The FASTQ files were first processed for quality control using FastQC (v0.12.1), and any identified adapter or poly-G or poly-A tail contamination was resolved using fastp (v0.23.4)[148,149]. The resulting sequences were aligned using STAR (v2.7.11b) to the original 2013 hg38 "soft-masked" reference genome indexed with the GENCODE knownGene transcript model (last updated 2023-06-28), both of which were downloaded from the UCSC Genome Browser[150–152]. The resulting BAM files were indexed using SAMtools[153].

Once the internally processed or author-provided count matrices were ingested, the resulting gene identifiers were either HUGO Gene Nomenclature Committee (HGNC) gene names or ENSEMBL gene IDs. We used `pyensembl`[154] and Ensemble release 108 to map all identifiers to HGNC gene names and combined any resulting multi-mapping transcripts. We also converted sample identifiers to harmonized PPC identifiers to allow for integration with viability and clinical data. Finally, we normalized the resulting count matrices to counts per million to account for differences in library size.

Drug harmonization

All drugs were matched to the PubChem database utilizing the PubChem Power User Gateway (PUG-REST)[155], a restful API. We queried the PubChem database based on the primary name of the drug used in the study. PubChem has two separate databases: the Substance database stores depositor-contributed information, while the Compound database contains on entry per unique standardized chemical structure[156]. Because of this we preferentially annotate our drugs with entries from the Compound database, but will use the Substance database if no match to the Compound database is found

We used two methods to match study drug names to PubChem; exact name match and search index match. Exact name match only returns an entry if the search term exactly matches that entry's name. The search index match is equivalent to typing a keyword into the PubChem web application search bar and selecting the best result.

When available in the database entry, we retrieved the SMILES string[157] for each drug. We also retrieved the parent compound for the entry when available, which is the primary organic component of the compound[158]. If two drugs had the same parent compound, or one drug is the parent of another, we merged them as one drug in our data model. For example, tetracycline hydrochloride (CID 54704426) and tetracycline metaphosphate (CID 54729668) were merged, as they share the same parent compound, tetracycline. Our algorithm for merging two drug entities is given in algorithm S1.

Drug target annotation

We assessed the feasibility of obtaining drug target annotations from several open-source resources, including DrugBank, OpenTargets, PubChem, and chemical supplier Selleck Chem[158–160]. To select

[1] https://github.com/tansey-lab/nf-rnaseq

bioRxiv preprint doi: https://doi.org/10.64898/2026.02.14.705918; this version posted February 17, 2026. The copyright holder for this preprint (which was not certified by peer review) is the author/funder, who has granted bioRxiv a license to display the preprint in perpetuity. It is made available under aCC-BY 4.0 International license.

a single resource to use, we manually compared annotated targets of kinase inhibitors, which often inhibit a broad spectrum of such proteins due to their structural and sequence conservation[161].

Table S3 compares the drug target annotations for these four resources to published kinase dissociation constants for multi-kinase inhibitor agerafenib. Only the targets identified by Selleck Chem are either the stated target the compound was designed to inhibit, RAF, or kinases on which agerafenib demonstrated single-digit nanomolar potency using a standard, pan-kinome in vitro screening assay[162]. This finding is consistent with our broader review and led us to use target annotations curated from Selleck Chem.

We attempted to match all drugs in our dataset to the World Health Organization's International Non-Proprietary Name (INN) list by using a partial match of the INN to the PubChem primary name and any synonymous names available under the PubChem substance or compound entry followed by a manual review [163,164]. We obtained drug mechanisms of action from the chemical supplier Selleck Chemicals, which provides drug target annotations for many of the compounds in its libraries. We included Selleck's L1100 inhibitor, L1300 FDA approved drug, and L1700 bioactive libraries, which contain 4,945, 3,067, and 9,125 compounds, respectively.

We then the queried the INN, PubChem name, and PubChem synonyms against the Selleck database on partial matches of the Selleck product name to the primary name or INN, or an exact match to any synonyms. During each query step, matches were reviewed manually to remove duplicates, disambiguate multi-mapping matches, and harmonize drug targets that differed slightly between the various libraries. For example, the non-inhibitor libraries included non-molecular targets that capture secondary effects of the drugs, such as "apoptosis related" or "autophagy," and were removed from all but drugs that directly target these pathways as their primary mechanism of action (e.g., elesclomol/apoptosis, chloroquine/autophagy). Once one associated name was annotated with a mechanism, no further identifiers were queried for that entry. For matches adjudicated as accurate, we retained all Selleck-provided targets.

Harmonizing immortalized cancer cell lines

In order to ensure the same immortalized cancer cell lines used in different studies were consistently identified, we queried all immortalized cell line identifiers against the Cellosaurus database using their REST API[102]. Cell lines which were matched against the database were annotated with their Research Resource Identifier (RRID)[165]. Two cell lines in different studies annotated with the same RRID in this way were considered to be same for the purposes of our downstream analysis. We used version 8.4 of GDSC1 and GDSC2.

Annotating immortalized cancer cell lines

We leveraged metadata in the Cellosaurus database to assign cancer diagnoses and metastatic status and sites to immortalized cell lines. Cellosaurus entries for cancer cell lines are often annotated with an National Cancer Institute Thesaurus (NCIt) ontology code[139]. OncoTree entries are annotated with synonymous NCIt codes, allowing us to assign OncoTree codes to these cell lines. Cellosaurus entries are also annotated with an indicator as to whether the cell line was established from metastatic tissue, and an UBERON ontology code indicating the anatomical site where the tissue was taken from[166]. We constructed a mapping of UBERON ontology terms to the MSK-MET classifications enabling us to annotate the cell lines with MSK-MET categories.

bioRxiv preprint doi: https://doi.org/10.64898/2026.02.14.705918; this version posted February 17, 2026. The copyright holder for this preprint (which was not certified by peer review) is the author/funder, who has granted bioRxiv a license to display the preprint in perpetuity. It is made available under aCC-BY 4.0 International license.

Non-parametric Bayesian tensor factorization model

Our model utilizes discrete grids over log-concentrations $c_1 < c_2 < \cdots < c_n$ as well as viabilities $0 = y_1 < y_2 < \cdots < y_n = 1$, where the assumption is that all observed concentrations fall within $[c_1, c_n]$ and all viabilities fall within $[0, 1]$. An observed viability y of a sample i treated with drug j at concentration c is modeled as

$$\begin{aligned}
y &\sim \text{Laplace}\left(\mu, \sigma\right) \\
\mu &= \pi\mu_{ij}^{(t)} + (1-\pi)\mu_{ij}^{(t+1)} \\
\sigma &= (\alpha\lambda_s + (1-\alpha)\lambda_{s+1})\sigma_{ij} \\
\mu_{ij}^{(t)} &= \left(\text{ReverseCumulSum}\left(\text{Softmax}\left(\beta_{ij}^{(1)}, \beta_{ij}^{(2)}, \ldots, \beta_{ij}^{(n-1)}\right)\right) \oplus 0\right)_t \\
\beta_{ij}^{(k)} &= \langle u_i, v_j^{(k)} \rangle \text{ for } k = 1, \ldots, n-1 \\
\sigma_{ij} &= \sigma_0 + \text{SoftPlus}\left(\langle x_i, z_j \rangle\right) \\
\sigma_0 &\sim \text{Gamma}(1, 1),
\end{aligned}$$

where $\pi \in [0, 1]$ and $t \in \{1, \ldots, n\}$ are the unique values satisfying $c = \pi c_t + (1-\pi)c_{t+1}$, and similarly, $\alpha \in [0, 1]$ and $s \in \{1, \ldots, n\}$ are the unique values satisfying $\mu = \alpha y_s + (1-\alpha)y_{s+1}$. $\langle u, v \rangle$ denotes the inner product between vectors u and v. The softmax function is the vector-valued function

$$\text{Softmax}(x_1, \ldots, x_m)_k = \frac{e^{x_k}}{\sum_{k'=1}^{m} e^{x_{k'}}}.$$

The reverse cumulative sum function is the vector-valued function

$$\text{ReverseCumulSum}(x_1, \ldots, x_n)_k = \sum_{k'=k}^{m} x_k.$$

The softplus function is the real-valued function satisfying

$$\text{SoftPlus}(x) = \log\left(1 + e^x\right).$$

We have further used the notation $v \oplus x$ to denote concatenation of vectors and scalars. u_i and $v_j^{(k)}$ are p_1-dimensional embeddings generated as

$$\begin{aligned}
u_{it} &\sim \mathcal{N}(0, \sigma_u^2) \text{ for } t = 1, \ldots, p_1 \\
v_{jt}^{(k)} &\sim \mathcal{N}(0, \sigma_v^2) \text{ for } t = 1, \ldots, p_1 \\
\sigma_u, \sigma_v &\sim \text{Gamma}(1, 1).
\end{aligned}$$

Similarly, x_i and z_j are p_2-dimensional embeddings generated according to

$$\begin{aligned}
x_{it} &\sim \mathcal{N}(0, \sigma_x^2) \text{ for } t = 1, \ldots, p_2 \\
z_{jt} &\sim \mathcal{N}(0, \sigma_z^2) \text{ for } t = 1, \ldots, p_2 \\
\sigma_x, \sigma_z &\sim \text{Gamma}(1, 1).
\end{aligned}$$

bioRxiv preprint doi: https://doi.org/10.64898/2026.02.14.705918; this version posted February 17, 2026. The copyright holder for this preprint (which was not certified by peer review) is the author/funder, who has granted bioRxiv a license to display the preprint in perpetuity. It is made available under aCC-BY 4.0 International license.

The vector $\lambda \in \mathbb{R}^n$, which non-parametrically shrinks the predicted variance as a function of the predicted mean satisfies

$$\lambda_s \sim \text{Gamma}(1, 1) \text{ for } s = 1, \ldots, n.$$

In addition to optimizing the ELBO loss on the observed viabilities, we optimize two contrastive losses over the mean sample embeddings, u_i, and mean drug-grid embeddings, $v_j^{(k)}$. Specifically, for the sample contrastive loss, we sample an index i, an index i^{pos} corresponding to the same primary site as i, and a set of indices $i_1^{\text{neg}}, \ldots, i_t^{\text{neg}}$ corresponding to primary sites that differ from i. The contrastive loss is given by

$$L_{\text{sample-contrast}}(i; i^{\text{pos}}; i_1^{\text{neg}}, \ldots, i_t^{\text{neg}}) = \log\left(\frac{\exp\left(\frac{1}{\tau}\text{cossim}\left(u_i, u_{i^{\text{pos}}}\right)\right)}{\exp(\frac{1}{\tau}\text{cossim}\left(u_i, u_{i^{\text{pos}}}\right)) + \sum_{s=1}^{t}\exp\left(\frac{1}{\tau}\text{cossim}\left(u_i, u_{i_s^{\text{neg}}}\right)\right)}\right),$$

where $\tau > 0$ is a temperature parameter, and $\text{cossim}(u, v) = \frac{\langle u,v\rangle}{\|u\|\|v\|}$ is cosine similarity.

Similarly, the drug contrastive loss at grid-point k is computed by sampling an index j, an index j^{pos} corresponding to a drug with a mechanism that is shared with j's mechanisms, and a set of indices $j_1^{\text{neg}}, \ldots, j_t^{\text{neg}}$ corresponding to drugs whose mechanisms do not overlap with those of j. Then the contrastive loss is given by

$$L_{\text{drug-contrast}}(j; j^{\text{pos}}; j_1^{\text{neg}}, \ldots, j_t^{\text{neg}}) = \log\left(\frac{\exp\left(\frac{1}{\tau}\text{cossim}\left(v_j^{(k)}, v_{j^{\text{pos}}}^{(k)}\right)\right)}{\exp(\frac{1}{\tau}\text{cossim}\left(v_j^{(k)}, v_{j^{\text{pos}}}^{(k)}\right)) + \sum_{s=1}^{t}\exp\left(\frac{1}{\tau}\text{cossim}\left(v_j^{(k)}, v_{j_s^{\text{neg}}}^{(k)}\right)\right)}\right).$$

Preprocessing and training

We restricted the corpus to experiments using drugs appearing in three or more studies, resulting in a vocabulary of 504 drugs. We clipped all viabilities between 0 and 1.

We applied the following preprocessing steps to filter out noisy experimental data. First, we filtered out any measurements for which there exist multiple experimental replicate points at the same dose which are ≥ 0.5 apart in measured viability space (clipped between 0 and 1). Second, given the downward monotonicity of the model, we restricted to curves that have a Spearman ρ correlation of ≤ 0.25; that is, we discarded dose-response measurements whose measurements trend strongly upwards as drug dose increases. Finally, we discarded experiments with contiguous measurements (in concentration space) which increase in magnitude at least 0.5 in measured viability space, using sudden upward jumps in viability as a proxy for high experimental noise. Due to low concordance with other datasets, we omitted Johansson et al. [10] from our model training and analysis. Dataset sizes throughout filtering steps is given in fig. S5.

All training runs were conducted with the following hyperparameters: $p_1 = 150$, $p_2 = 5$, $n = 25$, and $\tau = 0.1$. We set t, the number of negative sampled contrastive examples, to 10. We trained using stochastic variational inference (SVI)[167] for 500 epochs with a batch size of 50,000 using Adam[168] with a learning rate of 5×10^{-3}.

Calculating z-scores

For each (sample, drug) pair, our model produces a monotone piecewise linear curve estimating the mean dose-response at each concentration value. We first calculated the Area Under the Curve

bioRxiv preprint doi: https://doi.org/10.64898/2026.02.14.705918; this version posted February 17, 2026. The copyright holder for this preprint (which was not certified by peer review) is the author/funder, who has granted bioRxiv a license to display the preprint in perpetuity. It is made available under aCC-BY 4.0 International license.

(AUC) of each dose-response curve by numerically integrating the dose-response curve via the trapezoidal rule from $-9.2 \log_{10} \mu M$ to $4.0 \log_{10} \mu M$ in log-concentration space. Since each sample has its own intrinsic baseline sensitivity level, we normalize AUCs by calculating a robust z-score per sample. To do this, we fit a second order polynomial to the AUCs for a given sample across drugs. We then matched this polynomial to a Gaussian distribution using a second order Taylor expansion. This provides a mean and variance estimate for a Gaussian null distribution for drug effects on the sample. Individual drugs were then converted to z-scores using the inverse cumulative distribution function of the Gaussian. This approach was adapted from the literature on empirical Bayes hypothesis testing[56]. Unlike simple standardization (i.e. subtracting the mean and dividing by the standard deviation), this empirical null approach has the advantage of not allowing large effect sizes to skew the mean. In cases of samples' second-order polynomial being convex (comprising 2.1% of samples), preventing the match from polynomial to second-order Taylor approximation of a Gaussian, we fell back to simple standardization

Model evaluation

All data-holdout evaluation results are given for a whole-drug holdout five-fold cross-validation setup: each study's drugs were partitioned into five sets of equal cardinality (modulo differences from rounding), with a single fold's validation set comprising one such set for each constituent dataset in the corpus. Folds were generated such that no drug occurs in a validation set without being in the corresponding training set for at least some other study; that is, within a cross-validation fold, every drug in a validation set must have some training data from at least one study. This data-holdout setup probes the empirical performance of predicting full dose-response curves for (drug, sample) pairs on which the drug has not been observed on any samples from a given study, but both the sample and drug have some data in the training set.

The per-dataset-pair marginal utility calculations given in Fig. 2b were derived by comparing root mean-squared error (RMSE) on a scored evaluation dataset with and without a held-out dataset. That is, we first ran five-fold cross-validation with the full dataset, using the whole-drug holdout discipline as described above, evaluating the extent to which the model and dataset allow generalization to drugs not observed in a study. Then, for a held-out dataset h, we ran an additional five-fold cross-validation run omitting h from each of the five folds, but otherwise using the same folds so results are comparable. We then compared empirical performance on each probe dataset p both with and without held-out dataset h. In particular, we evaluated predictions on p using RMSE and calculated p-values with a one-sided binomial test, using the Benjamini–Hochberg procedure for false discovery rate adjustment.

The per-disease-type stratified Pearson r results in Fig. 2c and Fig. 2d were calculated in the same whole-drug-holdout five-fold cross-validation setup described above. In general a single drug may contribute to multiple groupings in Fig. 2d, as drugs in general have multiple mechanisms labeled. All Pearson r correlations were calculated at the level of individual viability point measurements in the whole-drug-holdout cross-validation setting.

Power analysis

Most drugs in the PPC dataset were tested on a small subset of samples; conversely, most samples only test a small subset of drugs. By accurately modeling drug response, we can prioritize top *in*

bioRxiv preprint doi: https://doi.org/10.64898/2026.02.14.705918; this version posted February 17, 2026. The copyright holder for this preprint (which was not certified by peer review) is the author/funder, who has granted bioRxiv a license to display the preprint in perpetuity. It is made available under aCC-BY 4.0 International license.

silico drugs for downstream validation, potentially enabling drug re-purposing and personalized treatment. Therefore, we investigated the model's power through the lens of nominating the most efficacious drug for a given sample via imputation.

We subsetted the PPC atlas to create a dataset with a high evidence level, which we call (PPC-strict). We select studies in which 1) at least 10 samples were tested, 2) at least 60 drugs were tested, and 3) $\geq 90\%$ of (sample, drug) pairs were tested on at least 3 drug concentrations. PPC-strict contains 1331 unique samples and 495 drugs, which are sourced from 11 studies: Driehuis et al.[4], Lee et al.[9], Sa et al.[14], Murumägi et al.[16], Peterziel et al.[20], Malani et al.[26], Pemovska et al.[27], Bottomly et al.[28], Powell et al.[37], Bruna et al.[38], Lau et al.[41] There is no sample overlap between any of the studies.

We simulated a 2-stage drug selection campaign on a set of unseen samples, where the goal was to discover the most potent drug for each sample as defined by the lowest half-maximal inhibitory concentration (IC50) as given by the model. In the first stage, k drugs were selected and experimentally validated on the new samples to establish a baseline. In the second stage, there was only budget to experimentally validate n additional drugs. Instead of handpicking the n drugs, we used data from the first stage to rank all drugs in the PPC atlas *in silico* and experimentally validated the top-ranked drugs.

To simulate such a campaign, we pre-trained separate PPC models on 10 studies in a hold-one-out fashion and performed drug selection on the held out study. In the first stage, we randomly selected k drugs from the held out study for observation and retrained the model. We then evaluated whether the PPC model could identify the most efficacious drug (lowest IC50) within the n drugs it nominated. Results are given in Fig. 2e.

Baseline models and comparisons

Baseline model comparisons are given in Fig. 2fgh. The *Bucketed mean* baseline estimator is a global dose-level mean curve estimate: that is, we bucketed into 20 equally-spaced buckets (in log-concentration space), from the lowest tested concentration in the dataset to the highest; within each bucket, we calculated the global mean across all curves and inferred that mean for all points in the bucket. This baseline provides a comparison to a global notion of the dataset's average dose-response curve.

The random forest baseline represents an ensemble of 100 random decision trees trained on bootstrap-resampled instances of the dataset; within a decision tree, intermediate nodes are required to have at least $10,000$ measurements to be split (approximately 1% of a fold's training set). The random forest is trained to regress to viability by minimizing ℓ_2 loss.

The neural network baseline model represents a simple multilayer feed-forward network neural network. Each drug and sample are assigned embeddings e_d and e_s, respectively; concentrations are bucketed into 20 equally-spaced buckets (in log-concentration space), from the lowest tested concentration in the dataset to the highest, and a measurement at a concentration is given an embedding e_c for its concentration bucket. The model is then $\text{FFN}(e_d \oplus e_s \oplus e_c)$, with $\oplus$ representing concatenation, and FFN being a sequence of alternating fully-connected affine transformations (with intermediate hidden layers of dimension h) and rectified-linear units, followed by a final fully-connected affine transform into a single scalar viability clipped to $[0, 1]$. The neural net was optimized via Adam[168] minimizing ℓ_2 loss. Within a fold, hyperparameters were selected via a random search (evaluated on 20% of that fold's training set) and the setting with optimal RMSE

bioRxiv preprint doi: https://doi.org/10.64898/2026.02.14.705918; this version posted February 17, 2026. The copyright holder for this preprint (which was not certified by peer review) is the author/funder, who has granted bioRxiv a license to display the preprint in perpetuity. It is made available under aCC-BY 4.0 International license.

out of 10 settings was chosen. Batch size was selected from $\{8, 16, 32, 64, 128, 256, 512, 1024\}$; the embedding dimension of e_d, e_s, and e_c (with all taking the same dimension in a given network) was selected from $\{16, 32, 64, 128\}$; the hidden width h was selected from $\{32, 64, 128, 256, 512, 1024\}$; the number of hidden layers was selected from $\{1, 2, 3, 4\}$; the number of training epochs was chosen from $\{1, 3, 5, 10, 15, 20\}$; the Adam learning rate was selected from $\{1 \times 10^{-3}, 5 \times 10^{-4}, 1 \times 10^{-4}, 5 \times 10^{-5}, 1 \times 10^{-5}\}$. After a fold's hyperparameters are selected, an MLP is trained on the fold's full training set using that setting.

All comparisons are done in a curve-holdout five-fold cross-validation setting. That is, we partition the dataset into five roughly-equal-sized subsets such that each (drug,sample) pair's measurements is entirely contained within a single fold.

Low-dimensional projections of sample and drug embeddings

The UMAP plots in Fig. 3b and Fig. 3c were generated from the sample embeddings u_i and concatenated drug embeddings $\bigoplus v_j^{(k)}$. Drug embeddings in Fig. 3c were annotated with the Selleck-provided mechanism for the drug; drugs with multiple mechanisms are labeled with the most common mechanism across the dataset.

Quantifying drug-site effects

To generate Fig. 3d, primary (non-metastatic) samples were grouped by site as annotated in the OncoTree hierarchy. Drugs most heavily comprising pure imputations were filtered out by restricting to drugs with at least 1,000 individual measurements across the dataset. Primary sites with fewer than 5 distinct samples were filtered out. We applied the empirical Bayes methodology described above (Methods) to each resulting group of z-scores, grouped per drug and per site and used Benjamini-Hochberg correction to derive q-values. Relative z-scores are given per-target; that is, each quantity represents the difference between the experimental group's mean z-score compared to the full mean z-score for that target. We restrict to solid tumors and filter out pancreas samples due to the sparsity of measurements for the site in the dataset.

To compare skin vs. non-skin samples in Fig. 3e, we restricted to drugs annotated with any targets in groupings in table S5. Healthy and non-primary (metastatic) samples were filtered out. Comparisons were taken between all remaining skin and non-skin samples for each drug annotated as targeting MEK or ERK.

The Beat Acute Myelogenous Leukemia (AML) cohort provided the most robust genomic annotations with 576 of the 629 samples (91.6%) for which we predicted drug z-scores possessing documented alterations. To assess the extent to which our model, which did not explicitly include genomic information, identified samples with clinically actionable alterations among Beat AML samples, we standardized fully-imputed z-score ranks by drug for agents with biomarker-defined mechanisms of action (Fig. 3f, top).

Comparing metastatic and primary samples

Mean AUC differences between metastatic and primary samples given in Fig. 4b were calculated per-drug per-disease; that is, the distribution of effect sizes was calculated by iterating over the full OncoTree hierarchy and, at each node, if that disease has both metastatic and primary samples

bioRxiv preprint doi: https://doi.org/10.64898/2026.02.14.705918; this version posted February 17, 2026. The copyright holder for this preprint (which was not certified by peer review) is the author/funder, who has granted bioRxiv a license to display the preprint in perpetuity. It is made available under aCC-BY 4.0 International license.

represented, we calculated the difference in mean dose-response AUC for each drug. We averaged across all diseases for each drug and calculated per-drug mean AUC difference between primary and metastatic samples across diseases. The population-level p-value was calculated via a one-sided binomial test.

To compare metastatic vs primary sample resistance to per disease standard of care drugs in Fig. 4c, the 2024 NCCN Treatment by Cancer Type was referenced for each OncoTree code. Only diseases with at least three metastatic samples were included in this analysis. Rhabdomyosarcoma (RMS) and Ewing Sarcoma (ES) were excluded from comparisons as the disease subtypes with the lowest predictive performance in the subset of the dataset used for comparison (Fig. 2c). Treatments ranked as “Preferred” or “Other Recommended” for a disease were split into their individual drugs, and corresponding drugs (if present) in the PPC database were labeled as as standard of care. For each sample, the AUCs for each standard of care drug were extracted. Many NCCN treatment guidelines for a disease consist of multiple drugs, for example “Weekly Paclitaxel and Carboplatin.” In such cases, The AUCs corresponding to each sample/drug/dose triplet were summed. The processed AUCs for all standard of care treatment regimens for a given OncoTree code were concatenated and rank normalized. A two-sided permutation test was applied comparing the PIC50s corresponding to metastatic samples treated with standard of care regimens and all primary samples treated with standard of care regimens.

To calculate per-drug correlations in Fig. 4d, we restricted analysis to non-brain metastases, diseases with both primary and metastatic samples in the dataset, and drug target annotations with at least three drugs represented in the dataset. We aggregated at the level of disease-metastatic-site pair; that is, for each drug, for each primary disease type (as given by OncoTree annotation), and for each metastatic site (as described above), we calculated the difference in mean AUC-based z-score between the metastatic samples and primary samples of the same disease. Each mechanism was thus represented as a k-element vector, with k the number of distinct (disease, metastatic-site) pairs present in the filtered dataset. Finally, we scaled this collection of k-element vectors into z-scores following the same method the AUC-based z-score calculation described above, transforming into z-scores at the (disease, metastatic-site) pair level. Fig. 4d gives the Pearson product-moment correlation coefficients between all pairs of k-element z-score vectors representing target annotations.

Fig. 4e gives per-mechanism primary/metastatic z-score differences at the disease level. For each disease type (most-granular OncoTree annotation) with at least two metastatic samples in the dataset, the mean difference in AUC-based z-scores between primary and metastatic samples was calculated. Fig. 4e depicts 90% uncertainty bars as given by a bootstrap resampling estimate. Mechanism-level p-values were calculated via two-sided Mann-Whitney U test with Benjamini-Hochberg correction.

Fig. 4f gives detailed information on drug response on metastatic samples compared to primary samples. Drug target annotations were grouped into coarse classes following the covariance structure given in Fig. 4d and labeled based on shared mechanisms; full mechanism groupings are given in table S5. The leftmost panel of Fig. 4f gives differences in drug response between metastatic and primary samples grouped by primary OncoTree type. Differences in z-scores were calculated for diseases with at least 3 metastatic and at least 3 primary samples present; p-values were given by a two-sided permutation test at the sample level and a Benjamini-Hochberg correction was applied. For the middle and right panel of Fig. 4f, each metastatic sample was compared to the mean z-score of the corresponding primary samples of that disease type; that is, for each drug, for each metastatic sample of disease type t, we compared the AUC-based z-score of that drug on that sample to the mean z-score of that drug on all primary samples of type t. Fig. 4f gives the mean of

bioRxiv preprint doi: https://doi.org/10.64898/2026.02.14.705918; this version posted February 17, 2026. The copyright holder for this preprint (which was not certified by peer review) is the author/funder, who has granted bioRxiv a license to display the preprint in perpetuity. It is made available under aCC-BY 4.0 International license.

all such differences, with p-values calculated via two-sided permutation test at the sample level. A Benjamini-Hochberg correction was applied to derive q-values.

Comparing ex vivo and cell line clustering

Fig. 5a, which depicts the UMAP projection of cell line and ex vivo samples for the dose-response model trained jointly on both types of data, shows qualitatively that ex vivo sample embeddings cluster more cleanly by disease type than than cell line sample embeddings To quantify this observation (Fig. 5b), we calculated the percentage of sample embeddings' neighborhoods with the same disease type as the sample. All samples with an OncoTree label with 5 or fewer samples were relabeled to the disease type one level up in the ontology's hierarchy; this process was iterated until each disease type has at least six samples. We then discarded any samples from diseases not represented in both ex vivo and cell line samples. We calculated each sample's 5 nearest neighbors by Euclidean distance in the sample embedding space. Samples were aggregated by disease type and, for each disease, we calculated the percentage of the union of its samples' neighborhoods that have the same disease label. To calculate per-disease significance levels (represented as colors in Fig. 5b), we applied Benjamini-Hochberg correction to p-values calculated via two-sided binomial tests with null probability equal to the dataset-level disease prevalence.

Quantifying cell line sample-neighborhood genomic patterns

The populations used to calculate the fraction of nearest neighbor samples with different mutations in Fig. 5c were restricted to samples from studies with genomics. BRCA1 and BRCA2 were combined into a single category "BRCA." Tumor mutation burden (TMB) in Fig. 5d was calculated as the raw number of mutations called for a sample.

Comparing ex vivo and cell line cytotoxicity

Comparative curve-level efficacy across disease types and drug mechanisms is given in Fig. 5e. We calculated response difference for all drug target annotations that are assigned to at least 3 drugs across the model's inventory of drugs. Samples were partitioned by most granular OncoTree level, with metastatic samples grouped separately from primary samples. Each (disease, mechanism) pair comprises a set of measurements for ex vivo samples and one for cell-line samples. The comparative efficacy of a mechanism's drugs on a disease (comparing between ex vivo and cell line model types) is given by the mean AUC-based z-score across all drugs annotated for that mechanism on all relevant samples. To calculate p-values for each group, we applied a two-sided permutation test, permuting sample labels $10,000$ times and measuring the percentage of permutations with absolute effect size at least as extreme as the observed. To combine per-disease p-values into aggregate mechanism-level p-values, we used Fisher's method; aggregate mechanism-level effect sizes are given by macro-averaged disease-specific effect sizes.

Measuring the effect of cell line data on ex vivo model performance

We quantified the effect of adding cell line data to empirical predictions on held-out ex vivo data Fig. 5f. As above, five-fold cross-validation of ex vivo performance was performed at the drug level; that is, a given constituent study's drugs is partitioned into five roughly-equal-sized sets with

bioRxiv preprint doi: https://doi.org/10.64898/2026.02.14.705918; this version posted February 17, 2026. The copyright holder for this preprint (which was not certified by peer review) is the author/funder, who has granted bioRxiv a license to display the preprint in perpetuity. It is made available under aCC-BY 4.0 International license.

one set held out for each of the five folds' train runs (that is, a drug in a fold's evaluation set was not observed on the set's samples during train). Folds were constructed so that no evaluation drug is fully absent from the train set. We ran ten train/evaluation folds in total—on the one hand, the five-fold validation run only on the ex vivo data; on the other hand, the same five splits but with the full corpus of cell line measurements over the model's drugs added to the training set. In all cases, the evaluation sets comprised only ex vivo measurements.

Density-estimate curves in Fig. 5f are Gaussian kernel-smoothed estimations on a per-disease basis. For each disease (i.e. the most granular OncoTree annotation), we calculated ex vivo samples' held-out root-mean squared error (RMSE) across folds. Letting e_d denote disease d's RMSE across all measurements on all relevant samples in the ex vivo-only cross-validation setup, and e'_d denote the RMSE on the cross-validation run which includes cell line data in train, Fig. 5f gives the per-mechanism distributions of relative error differences $(e_d - e'_d)/e_d$ across diseases d. Per-group q-values were calculated via applying Benjamini-Hochberg correction to p-values calculated via two-sided binomial tests on per-disease binarized improvement indicators (i.e. binary variables taking value 1 if and only if empirical RMSE is lower with cell line data added to train), with null binomial $p = 0.5$.

Comparing cycling and signaling targeting drugs across cell lines and ex vivo samples

To compare comparative efficacy of drugs targeting cell cycling and cell signaling pathways broadly construed in Fig. 5g, we restrict to primary samples and we use the grouping of targets given in table S4. For each drug, samples are ranked by their z-score sensitivity and ranks are normalized to [0, 1], with higher values indicating higher efficaciousness/sensitivity. Samples are grouped by OncoTree, and only OncoTrees with at least 3 ex vivo and 3 cell line samples are included. The individual lines connect median normalized rank in the corresponding sample set, with the per-model-construct aggregate given by the median of such values.

Comparing ex vivo RMSE improvement and construct-type sensitivity

To compare relative RMSE improvement to mean z-score difference between ex vivo and cell line models in Fig. 5h, we calculate RMSE improvement for every (disease-indication, mechanism-of-action) pair with observed data, treating primary and metastatic cancers as different subclasses. We then compare to the mean z-score difference as given in Fig. 5e.

Comparing primary and metastatic z-score differences between ex vivo and cell line samples

To compare the difference in z-score between primary and samples as given in Fig. 5i, we restrict to OncoTree codes that have at least 2 primary samples a and 2 metastatic samples in both cell line and ex vivo datasets and directly compare average z-score discrepancies.

Comparing drug-drug similarity across ex vivo and cell lines

To quantify the extent to which drugs behave similarly across cell line and ex vivo samples as given in Fig. 5j, we first calculate drug-drug covariance across diseases (fig. S25), where each entry is the Pearson's product-moment correlation coefficient across per-OncoTree mean z-scores on that drug. We calculate one covariance matrix for cell line samples and one for ex vivo samples (using

bioRxiv preprint doi: https://doi.org/10.64898/2026.02.14.705918; this version posted February 17, 2026. The copyright holder for this preprint (which was not certified by peer review) is the author/funder, who has granted bioRxiv a license to display the preprint in perpetuity. It is made available under aCC-BY 4.0 International license.

the same dimensions so they are directly comparable). We then, for each row index i, calculate the Pearson r of the ith row of the two individual covariance matrices, giving us a quantification of how similarly the drug corresponding to that row behaves across the two model construct types.

Comparing additive effects across ex vivo and cell lines

To assess the potentially confounding effects of media differences, we annotated 50 media additives as previously described. For the 42 additives that occurred in three or more studies, we performed independent two-covariate linear regressions predicting the average drug target z-scores per sample using one-hot encoded variables indicating the presence or absence of a given additive and whether or not the sample was a cell line or ex vivo construct and including an intercept term (fig. S23(a), fig. S24). Hierarchical agglomerative clustering was performed on the media additives to identify groups with similar coefficient profiles across drug targets using Ward's minimum variance method and Euclidean distance. Given the highly collinear nature of additives, construct type, and study ID, we performed ℓ_1-regularized multiple regressions predicting the the average drug target z-scores per sample using one-hot encoding for these covariates and an intercept term (fig. S23(b)). We used five-fold cross-validation to determine an optimal regularization parameter and bootstrap resampling to determine a standard error of the mean of the ex vivo coefficient. To assess concordance between the ordering of the negative one-hot encoded ex vivo coefficient by drug target from this analysis and the ordering of deviations between cell line and ex vivo z-scores by drug target as shown in Fig. 5(e), we computed the Spearman correlation coefficient and its corresponding p-value between these two sets.

Foundation Model Design

Architecture. We use a BERT[118]-like architecture with 8 transformer blocks, hidden layer size of 384, intermediate hidden size of 512 and 8 attention heads in each transformer. The foundation model is designed for few-shot learning on structured data: rather than requiring gradient-based optimization at inference time, it directly takes as input a small labeled input set and an unlabeled query set, and outputs predictions for the query samples. This formulation enables efficient adaptation to new tasks with very few examples, without any model parameter updates or tuning of optimization hyperparameters.

Model Inputs. In our application, each model input corresponds to a specific biological sample (PDO, PDC, or PDCL) for which viability measurements are available under various drug and dose conditions. The input is structured as paired input and query sets. The input set consists of a small number of labeled triplets (drug, dose, viability), while the query set contains unlabeled pairs $(\text{drug}', \text{dose}')$ for which the model predicts viability. This setup allows the model to infer viability responses for unseen drug–dose combinations based solely on a few measured examples from the same biological sample.

Formally, denoting the model by f, the labeled input set by $\mathcal{S}_{\text{input}}$ of size $n \in \mathbb{N}$, and the unlabeled

bioRxiv preprint doi: https://doi.org/10.64898/2026.02.14.705918; this version posted February 17, 2026. The copyright holder for this preprint (which was not certified by peer review) is the author/funder, who has granted bioRxiv a license to display the preprint in perpetuity. It is made available under aCC-BY 4.0 International license.

query set by $\mathcal{S}_{\text{query}}$ of size $m \in \mathbb{N}$, the predicted viabilities for the query set satisfy:

$$\begin{aligned}
&\left\{\text{predicted-viability}'_1, \ldots, \text{predicted-viability}'_m\right\} = f\begin{pmatrix} \text{input} = \mathcal{S}_{\text{input}} \\ \text{query} = \mathcal{S}_{\text{query}} \end{pmatrix} \\
&\mathcal{S}_{\text{input}} = \left\{(\text{drug}_1, \text{dose}_1, \text{viability}_1), \ldots, (\text{drug}_n, \text{dose}_n, \text{viability}_n)\right\} \\
&\mathcal{S}_{\text{query}} = \left\{(\text{drug}'_1, \text{dose}'_1), \ldots, (\text{drug}'_m, \text{dose}'_m)\right\}.
\end{aligned} \tag{S1}$$

Both input and query sets are encoded using sequence of tokens for the drug, dose and viability. The drug tokens are given by an embedding layer, which is a dictionary retuning a vector of size 128 for each drug. For the dose and viability embedding, we also use vector of size 128. To encode the continuity of these values, the embedding is given by a linear combination of Fourier features with learnable parameters matrix $A \in \mathbb{R}^{128\times128}$ and vector $w \in \mathbb{R}^{128}$ and input the scalar "dose" normalized in $[0, 1]$ such that:

$$\text{dose-embedding} = \left(\sum_{k=1}^{128} A_{1k} \cos(w_k \cdot \text{dose}), \ldots, \sum_{k=1}^{128} A_{128k} \cos(w_k \cdot \text{dose})\right),$$

The same type of embedding is used for viability. Note that dose refers to the min-max scaled log-concentration. Finally the three embeddings are concatenated to form the embedded token of size 384 for each (drug, dose, viability) triplet in the input and query sequence.

Training on PPC. During training, cross-attention is performed between the tokens of the input and query sequences. No self-attention is performed between tokens of the same sequence. A linear head is used on top of the final embedding of each query token to predict the viability of the unlabeled (drug, dose) query pairs. To provide a sample embedding we average the final token embedding over the query sequence. ℓ_1 loss is used between predicted and observed viabilities.

The training dataset consists of approximately $n_{\text{sample}} \simeq 5000$ splits of the PPC dataset, each corresponding to the (drug, dose, viability) measurements of a single biological sample. One training epoch comprises 100,000 gradient update steps, each iterating over randomly selected samples. At each step, the measurements of the selected sample are randomly divided into two parts of random size: the labeled input set $\mathcal{S}_{\text{input}}$ and the unlabeled query set $\mathcal{S}_{\text{query}}$. The true viability values of the query set are held out and used to compute the loss between them and the predicted viabilities obtained according to Equation S1. The model is trained on 200 epochs with learning rates $1e{-}5$ using the Adam optimizer[168].

Inference on New Samples. At inference time, the model operates in the same few-shot fashion as during training. Given a new biological sample with a small set of observed (drug, dose, viability) measurements (input set) and a set of query conditions (query set), the model directly predicts the viabilities of the query conditions without any further gradient updates. This property makes inference both computationally efficient and convenient, as no optimization parameters (e.g., learning rate, batch size) need to be specified.

Foundation Model Results

Experimental Setup (Fig. 6b). We train three foundation models (FMs), each trained with a different study held out. For each held-out study, all samples from that study are removed from the

bioRxiv preprint doi: https://doi.org/10.64898/2026.02.14.705918; this version posted February 17, 2026. The copyright holder for this preprint (which was not certified by peer review) is the author/funder, who has granted bioRxiv a license to display the preprint in perpetuity. It is made available under aCC-BY 4.0 International license.

PPC dataset during training. This setup simulates a realistic use case scenario in which a practitioner aims to infer the dose–response curve of a set of drugs for a new biological sample from only a few experiments.

Few-Shot Dose–Response Inference. For each held-out study, we conduct a few-shot inference analysis to evaluate how efficiently the foundation model predict dose–responses from a small number of observations. For each sample in the study, we randomly select $n_{\text{few-shots}}$ (drug, dose, viability) measurements among those available in the dataset, forming the input set to the foundation model. The query set is defined as a different set of (drug, dose) measurements, not contained in the input set. Spearman correlation is computed on the query set between the predicted viability and the ground truth viability. We conduct the analysis for $n_{\text{few-shots}} \in \{10, 50, 100, 200\}$, with five random repetitions for each value of $n_{\text{few-shots}}$. Fig. 6b reports the evolution of Spearman correlation with respect to $n_{\text{few-shots}}$, with error bars representing two standard deviations across repetitions.

Baselines. We compare the foundation model (FM) and its finetuned version (FM++) against three baselines: TabPFN, XGBoost, and Ridge regression. For FM++, a first inference is performed using the few-shot data as both input and query sets. The linear head is then finetuned to match the predictions with the viability values of the few-shot data. TabPFN operates in the same few-shot fashion as the FM: for each sample, it takes as input the $n_{\text{few-shots}}$ input set and the corresponding query set, and directly outputs the predicted query viabilities. XGBoost and Ridge regression, in contrast, require explicit training. For each sample, these models are fitted on the input set of $n_{\text{few-shots}}$ observations and then used to predict the query viabilities. Hyperparameters are selected via grid search with cross-validation. For XGBoost the number of estimators is tuned from the set {100, 200, 300} and the learning rate from the set {0.01, 0.1, 0.2}, for Ridge the regularization trade-off parameter alpha is tuned from the set {0.001, 0.01, 0.1, 1.0, 10.0, 100.0}. To provide a drug embedding to these baselines, we use the Morgan fingerprint[169] over 512 bits. As we observe poor performances of TabPFN with such a large number of features, we extract the 50 first components of the PCA from the matrix of Morgan fingerprint features for every drugs in the PPC dataset.

Embedding-Based Prediction of Molecular Features (Fig. 6c–d). We focus on the Beat AML cohort, which provides paired mutation profiles and gene expression measurements for every sample. To obtain sample-level representations, we extract embeddings from the foundation model (FM) by averaging the final token embeddings over the sequence of queries.

Prediction of Gene Mutations. Using these fixed embeddings, we train linear probes to predict binary mutation status. To ensure sufficient prevalence, we retain genes with at least 10 mutated samples in Beat AML. We include only samples with available mutation calls and at least 50 profiled drugs (covering over 80% of the cohort), which yields more stable embeddings. For each sample, embeddings are recomputed for $n_{\text{few-shots}} \in \{10, 50, 100, 200\}$ as in the few-shot setup. Probes are evaluated with 5-fold cross-validation, and we report the mean AUC across folds. Each configuration is repeated 5 times with independent random selections of doses, and metrics are averaged across repetitions. Fig. 6c plots AUC as a function of $n_{\text{few-shots}}$ and highlights the 4 genes with the highest performance.

Prediction of Gene Expression. For continuous gene expression, we aggregate signals at the pathway level using Hallmark gene sets. Let $M \in \mathbb{R}^{n_{\text{samples}} \times n_{\text{genes}}}$ denote the expression matrix; we normalize each row to sum to 10^6 and then apply $\log(M + 1)$. For each pathway, we subset to its member genes and compute the first principal component, yielding a single pathway score per sample. We then fit Ridge regression models (regularization parameter $\alpha = 1$) to predict pathway scores from the 768-dimensional embeddings. Evaluation mirrors the mutation task: 5-fold cross-validation with

bioRxiv preprint doi: https://doi.org/10.64898/2026.02.14.705918; this version posted February 17, 2026. The copyright holder for this preprint (which was not certified by peer review) is the author/funder, who has granted bioRxiv a license to display the preprint in perpetuity. It is made available under aCC-BY 4.0 International license.

5 random dose selection repetitions, reporting the average performance. Fig. 6d shows performance as a function of $n_{\text{few-shots}}$, highlighting the 12 most accurately predicted pathways.

Multi-Omics Analysis (Fig. 6e). We test whether the model produces consistent signals across gene mutation, pathway activity, and targeted drug response. We define three analyses:

1. KRAS with the Hallmark KRAS signaling up pathway and the MEK inhibitor Trametinib.
2. NPM1 with the Hallmark Apoptosis pathway and the Bcl-2 inhibitor Venetoclax.
3. TP53 with the Epithelial–Mesenchymal Transition (EMT) pathway and the MDM2 inhibitor nutlin-3A.

For each sample, we fix $n_{\text{few-shots}} = 100$. We select three dose measurements per tested drug from the dataset. This small set is used both to compute the embedding and to perform few-shot dose–response inference. We run the foundation model and extract the 384-dimensional embedding. We then fit linear probes on these embeddings in a leave-one-sample-out scheme. One probe predicts the mutation status of the gene (KRAS, NPM1 or TP53). The other probe predicts the pathway expression score defined earlier from Hallmark gene sets (KRAS signaling up, Apoptosis or EMT). For the held-out sample, we output a mutation logit and a pathway score.

We also infer drug response under the same few-shot setting. For each sample, we use the model to predict viabilities for the targeted drug over the test dose grid. We integrate these predictions to obtain a predicted AUC for that drug. Lower predicted AUC indicates higher sensitivity.

Fig. 6e is organized by columns and rows. Each column corresponds to one analysis and each row to one Omic type. Each subplot provide a boxplot comparison of the wild-type and mutant groups. We assess differences with a one-sided Mann–Whitney U test and report the p-value above each panel.

Quantifying study-level batch effects

The per-study comparisons in fig. S15 were restricted to most-granular OncoTree annotations such that the disease has at least three samples and is present in at least two studies. All drugs in the vocab described in Methods and used throughout the above were compared.

Measuring per-study-pair marginal effects

fig. S6 gives the per-dataset marginal absolute RMSE differences given by adding each dataset to the remaining dataset; that is, for each pair of distinct datasets (d_h, d_s), we calculated the absolute RMSE difference on the scored dataset d_s with and without the held-out dataset d_h. These are the effect sizes corresponding to the q-values in Fig. 2b. All RMSE values were calculated in the whole-drug-holdout five-fold-cross validation setup used in Fig. 2b.

Quantifying bioactivity across diseases and drugs

fig. S16 gives mean z-scores grouped by (overlapping) drug targets and (non-overlapping) disease annotations. Note CHOL and HCC are grouped together, as are LUSC and LUAD—a number of similar diseases have similar drug responses by model imputation, when viewed in the aggregate.

bioRxiv preprint doi: https://doi.org/10.64898/2026.02.14.705918; this version posted February 17, 2026. The copyright holder for this preprint (which was not certified by peer review) is the author/funder, who has granted bioRxiv a license to display the preprint in perpetuity. It is made available under aCC-BY 4.0 International license.

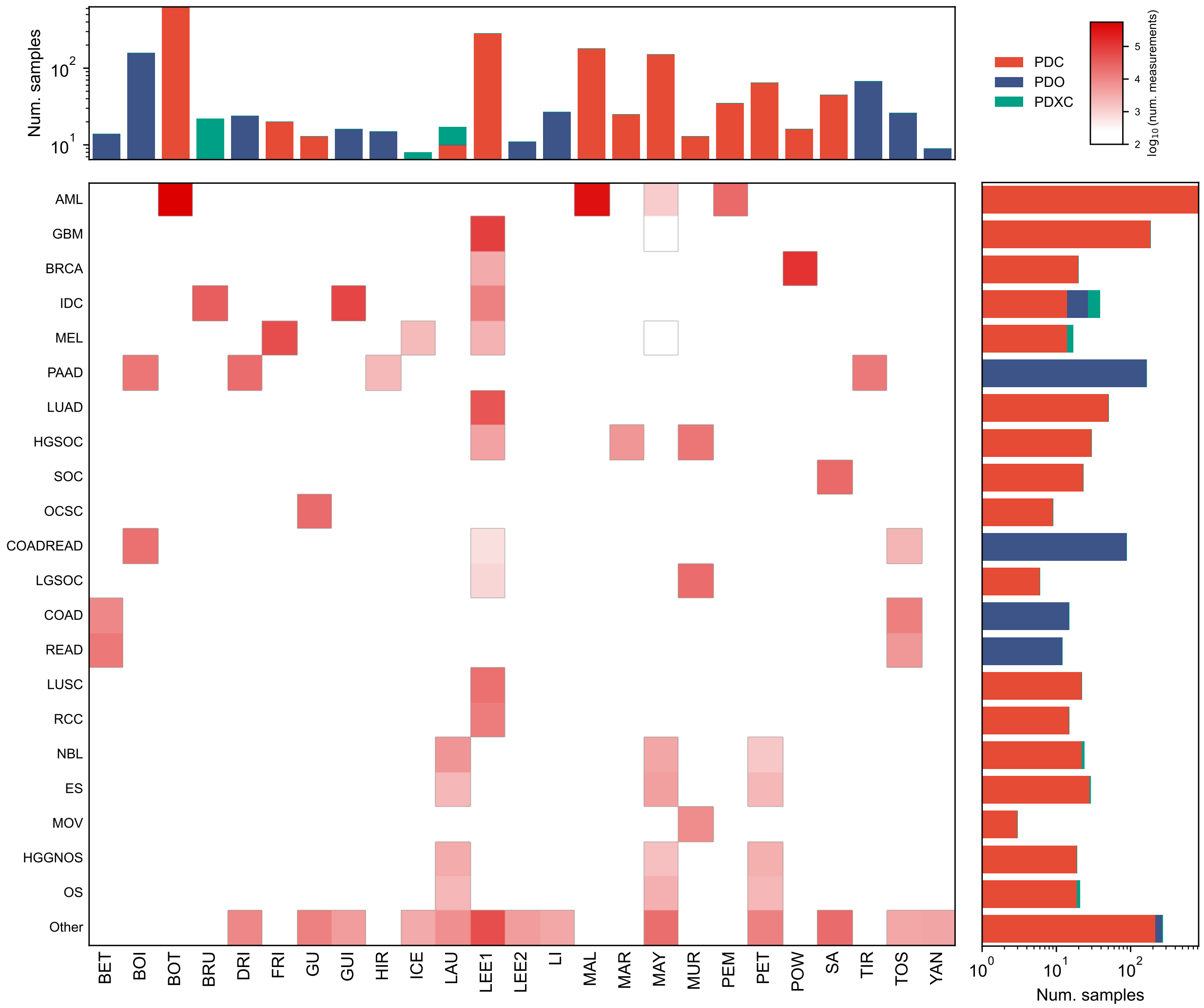

Figure S1: Disease coverage varies across studies. The number of individual viability measurements for the most common disease types, broken down by constituent study. Histograms represent the total number of distinct samples for each study (columns) and disease (rows), colored by model construct (study abbreviations: table S1).

bioRxiv preprint doi: https://doi.org/10.64898/2026.02.14.705918; this version posted February 17, 2026. The copyright holder for this preprint (which was not certified by peer review) is the author/funder, who has granted bioRxiv a license to display the preprint in perpetuity. It is made available under aCC-BY 4.0 International license.

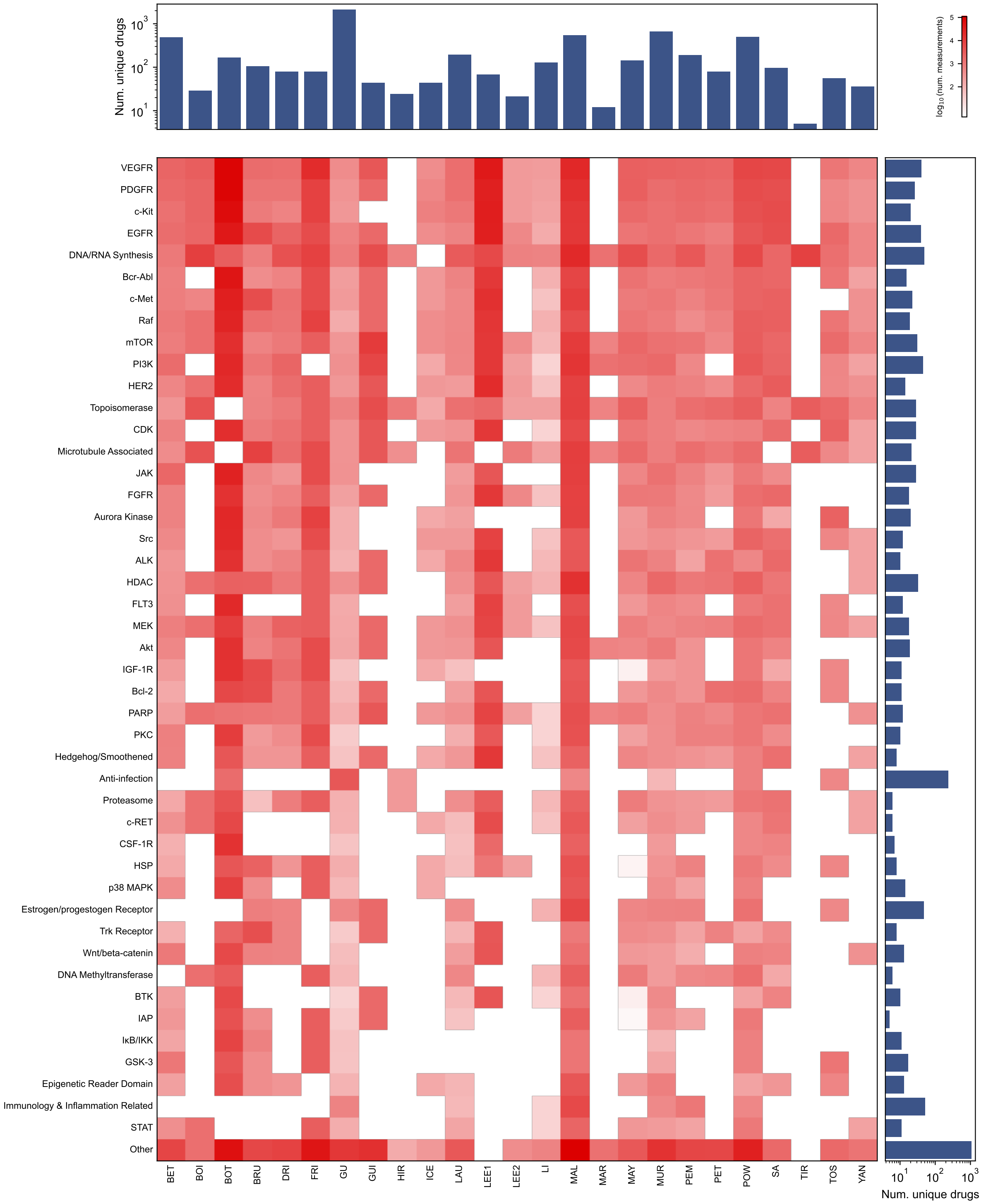

Figure S2: Drug target coverage varies across studies. The number of raw viability measurements stratified by annotated drug target and study. The number of distinct drugs are given as histograms for each study (column) and target (row). As drugs are labeled in general with multiple targets, a given viability measurement may appear in multiple rows (study abbreviations: table S1).

bioRxiv preprint doi: https://doi.org/10.64898/2026.02.14.705918; this version posted February 17, 2026. The copyright holder for this preprint (which was not certified by peer review) is the author/funder, who has granted bioRxiv a license to display the preprint in perpetuity. It is made available under aCC-BY 4.0 International license.

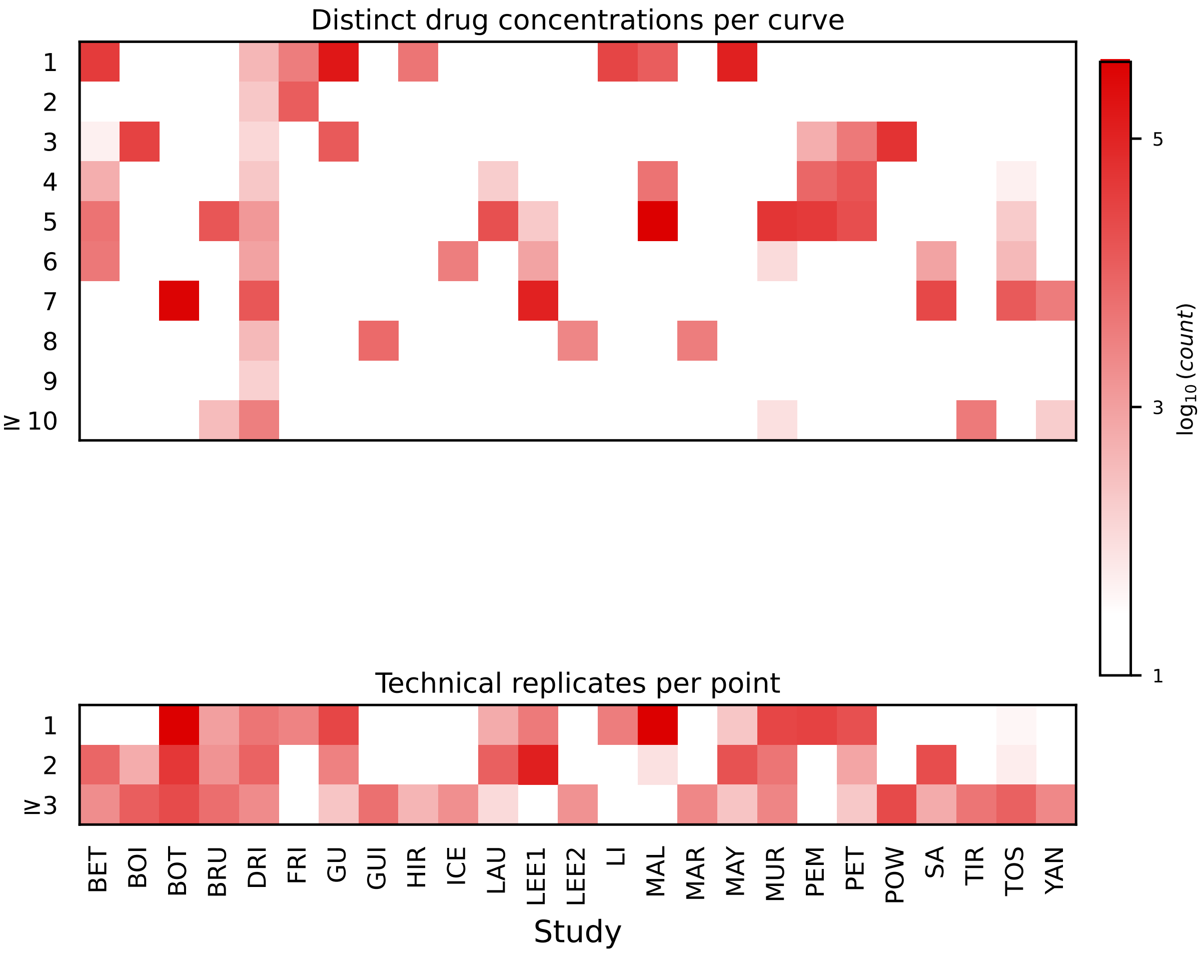

Figure S3: Assay design varies across studies. The numbers of distinct dose concentrations per curve (top) and technical replicates per point (bottom) across different datasets, restricted to single-drug dose-response assays (study abbreviations: table S1).

bioRxiv preprint doi: https://doi.org/10.64898/2026.02.14.705918; this version posted February 17, 2026. The copyright holder for this preprint (which was not certified by peer review) is the author/funder, who has granted bioRxiv a license to display the preprint in perpetuity. It is made available under aCC-BY 4.0 International license.

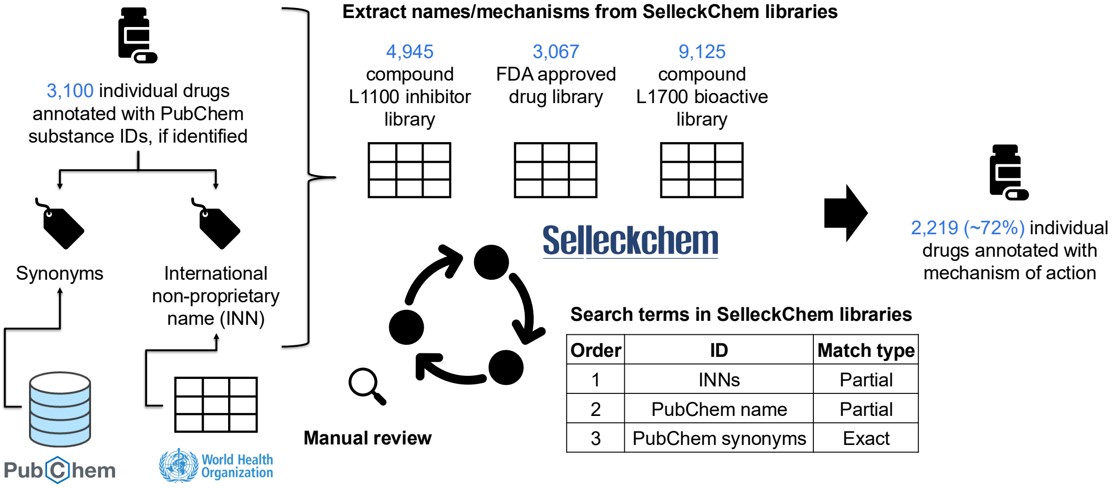

Order	ID	Match type
1	INNs	Partial
2	PubChem name	Partial
3	PubChem synonyms	Exact

Figure S4: Drug target annotations are harmonized from heterogeneous sources in a semi-automated process.

bioRxiv preprint doi: https://doi.org/10.64898/2026.02.14.705918; this version posted February 17, 2026. The copyright holder for this preprint (which was not certified by peer review) is the author/funder, who has granted bioRxiv a license to display the preprint in perpetuity. It is made available under aCC-BY 4.0 International license.

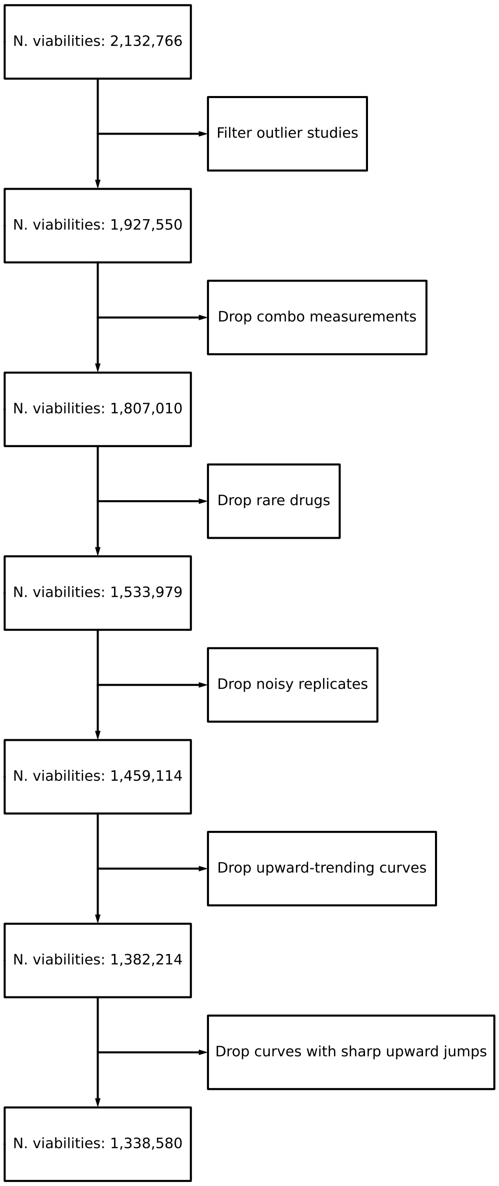

Figure S5: The ex vivo dataset is filtered for quality before model training. The ex vivo dataset size during individual data filtering steps is given.

bioRxiv preprint doi: https://doi.org/10.64898/2026.02.14.705918; this version posted February 17, 2026. The copyright holder for this preprint (which was not certified by peer review) is the author/funder, who has granted bioRxiv a license to display the preprint in perpetuity. It is made available under aCC-BY 4.0 International license.

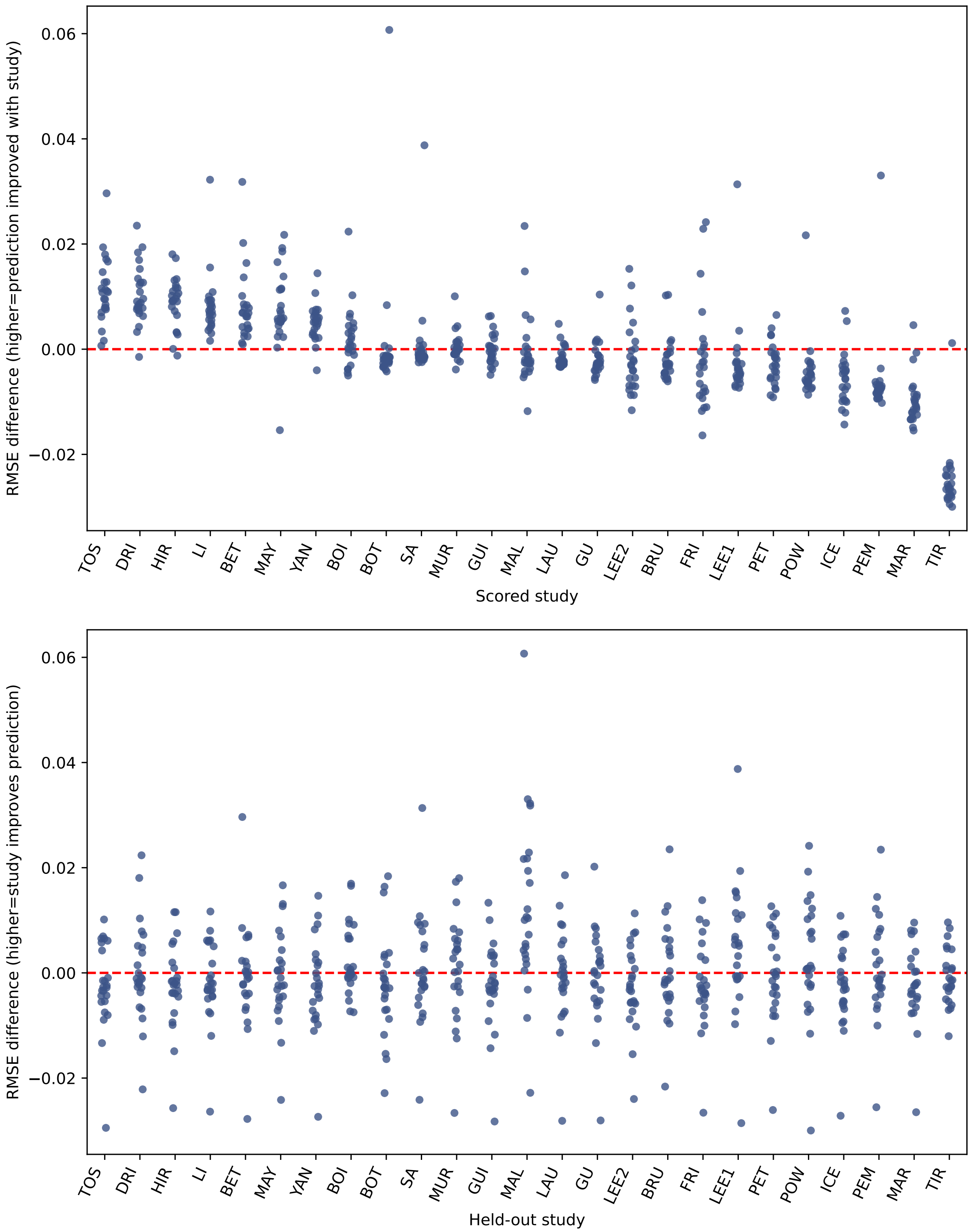

Figure S6: Studies exhibit heterogeneity in pairwise empirical modeling utility. Per-dataset pair absolute RMSE differences ($e_h - e_f$, with e_h the RMSE ablating the relevant held-out study and e_f the RMSE with the full dataset), grouped by scored study (top) and held-out study (bottom).

bioRxiv preprint doi: https://doi.org/10.64898/2026.02.14.705918; this version posted February 17, 2026. The copyright holder for this preprint (which was not certified by peer review) is the author/funder, who has granted bioRxiv a license to display the preprint in perpetuity. It is made available under aCC-BY 4.0 International license.

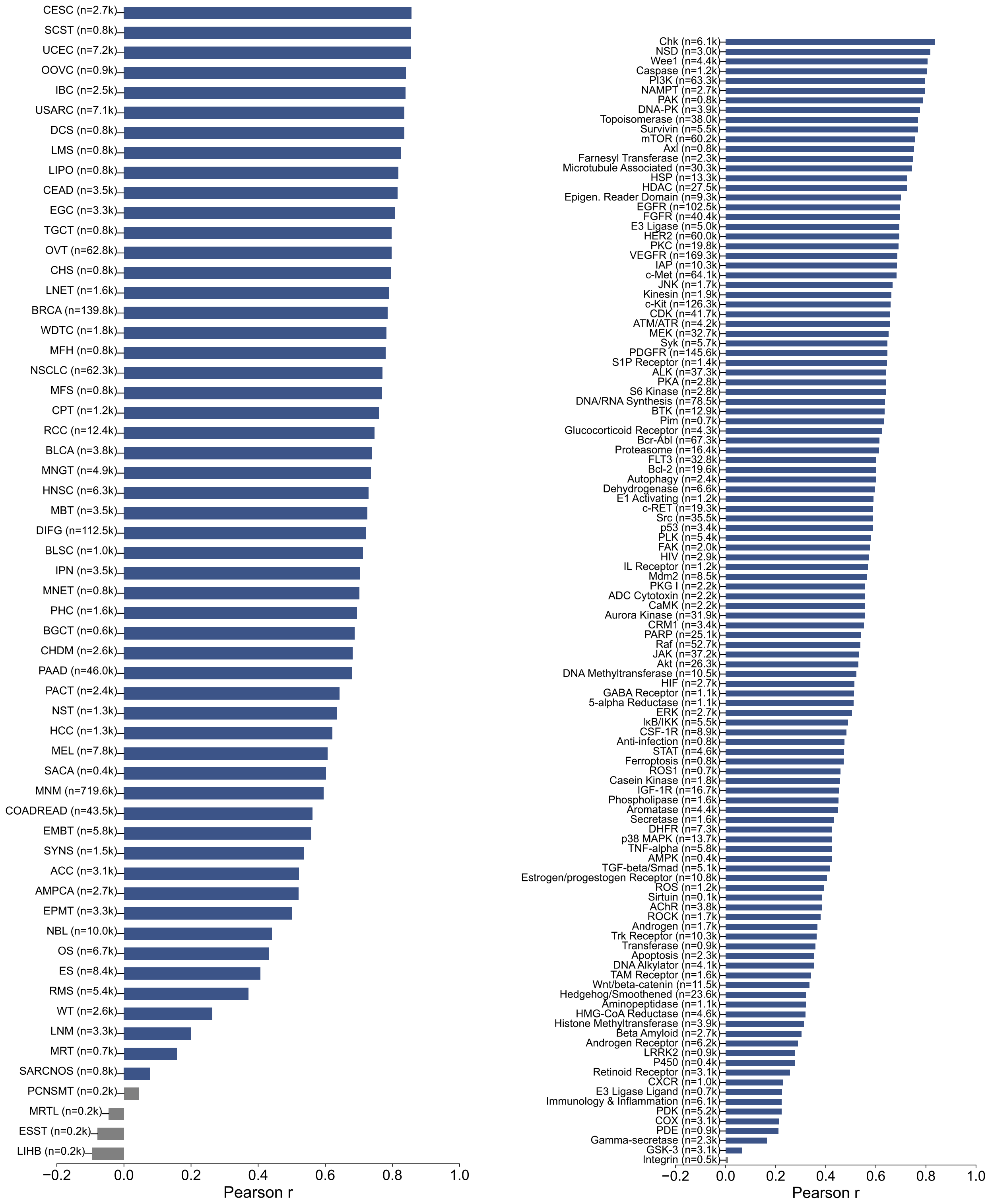

Figure S7: Empirical model performance varies across disease indications and drug targets. Pearson correlation stratified by disease subtype (left) and drug target (right), as depicted in Fig. 2cd, with individual entries labeled.

bioRxiv preprint doi: https://doi.org/10.64898/2026.02.14.705918; this version posted February 17, 2026. The copyright holder for this preprint (which was not certified by peer review) is the author/funder, who has granted bioRxiv a license to display the preprint in perpetuity. It is made available under aCC-BY 4.0 International license.

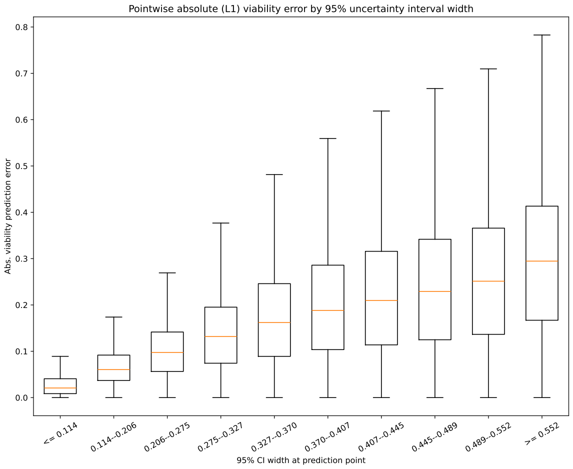

Figure S8: Model prediction error is monotone in model uncertainty. Pointwise mean absolute error for all measurements in the dataset, stratified by the model's 95% uncertainty interval width at that point. Orange line: median; boxes: first to third quartile; whiskers: 1.5 times inter-quartile range.

bioRxiv preprint doi: https://doi.org/10.64898/2026.02.14.705918; this version posted February 17, 2026. The copyright holder for this preprint (which was not certified by peer review) is the author/funder, who has granted bioRxiv a license to display the preprint in perpetuity. It is made available under aCC-BY 4.0 International license.

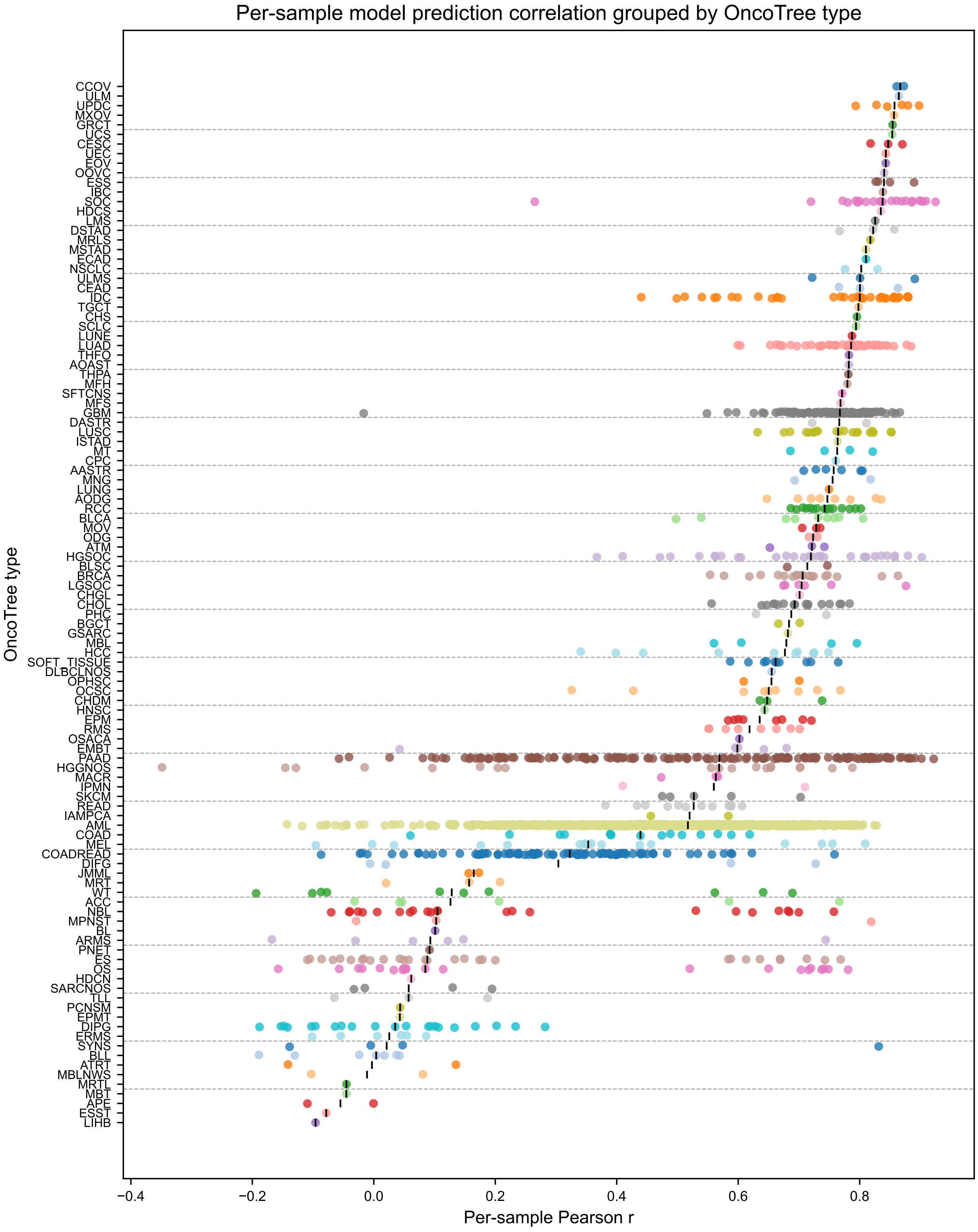

Figure S9: Disease indications exhibit intra- and inter-group heterogeneity in model performance. Per-sample Pearson correlation by disease type, with group medians (black bars).

bioRxiv preprint doi: https://doi.org/10.64898/2026.02.14.705918; this version posted February 17, 2026. The copyright holder for this preprint (which was not certified by peer review) is the author/funder, who has granted bioRxiv a license to display the preprint in perpetuity. It is made available under aCC-BY 4.0 International license.

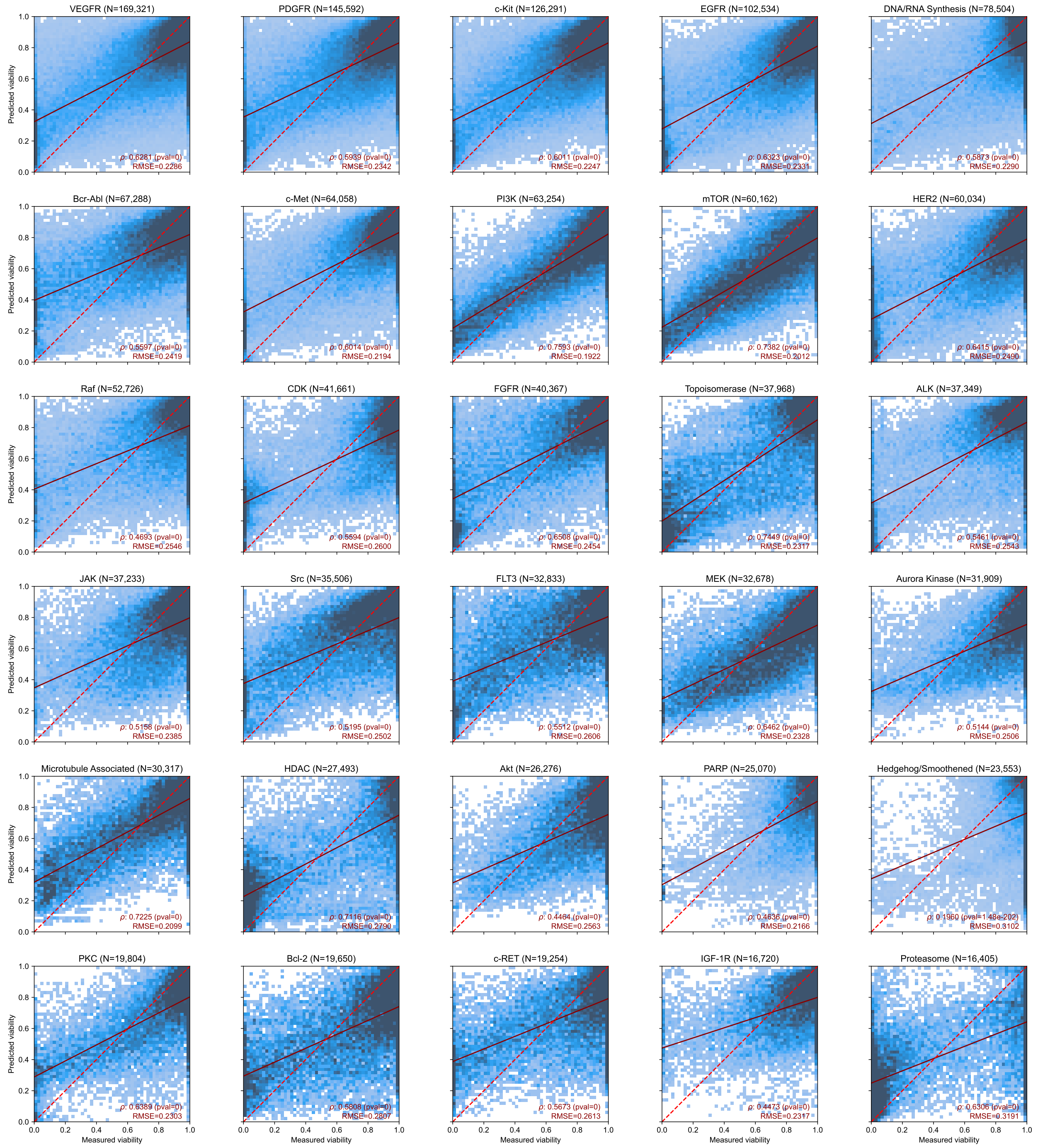

Figure S10: Drug targets exhibit heterogeneity across assay design, measured response, and prediction accuracy. (1 of 4) Predicted vs. measured per-point viability densities, stratified by drug class, with Spearman rank-correlation coefficient, root-mean-squared prediction error, and OLS-regression fit (alongside the $y = x$ identity line) for each class. Plots are ordered by measurement count in the dataset. All p-values in fig. S10–fig. S13 are given by two-sided t tests on the Spearman ρ coefficients.

bioRxiv preprint doi: https://doi.org/10.64898/2026.02.14.705918; this version posted February 17, 2026. The copyright holder for this preprint (which was not certified by peer review) is the author/funder, who has granted bioRxiv a license to display the preprint in perpetuity. It is made available under aCC-BY 4.0 International license.

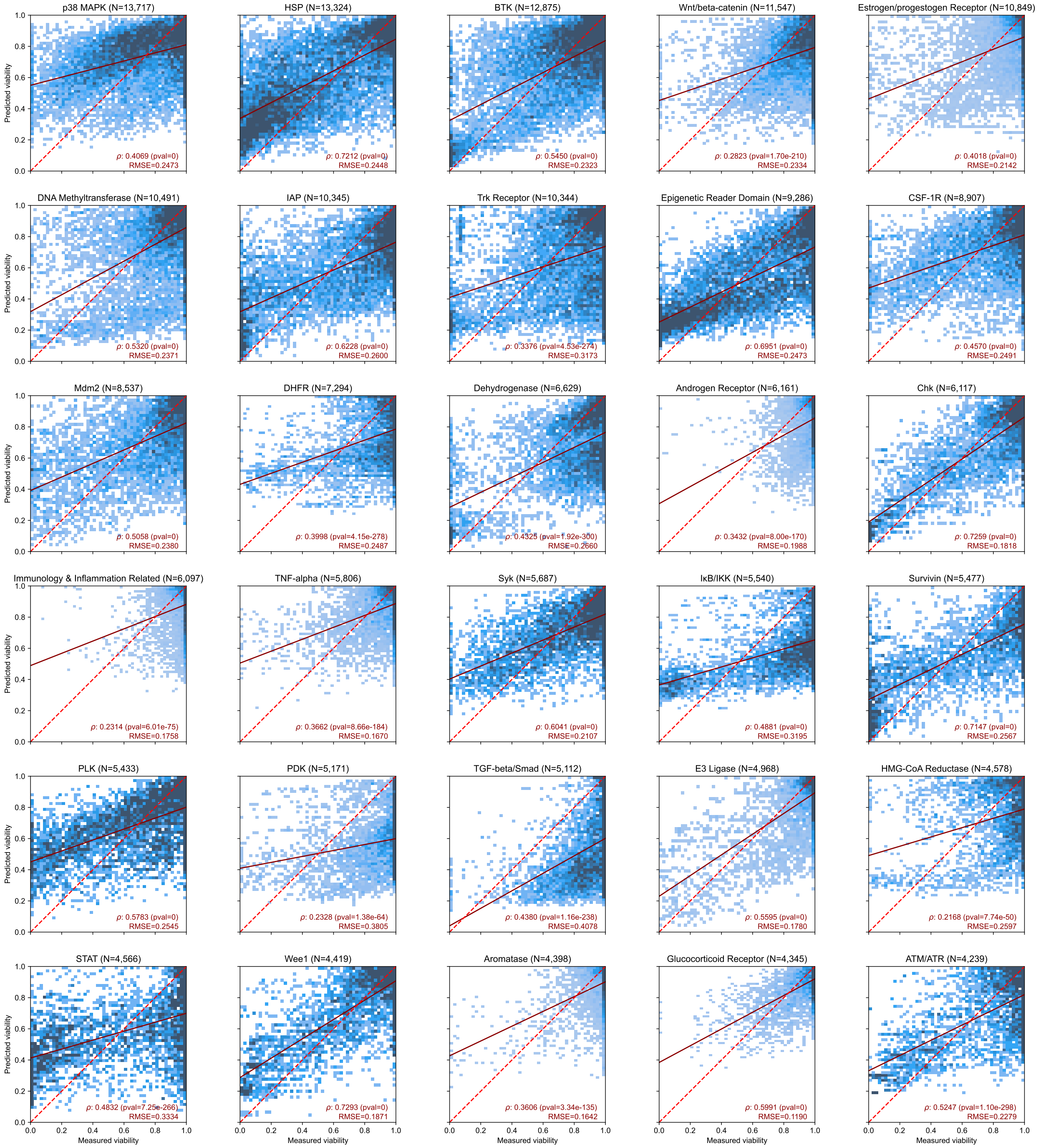

Figure S11: (2 of 4) Continuation of fig. S10.

bioRxiv preprint doi: https://doi.org/10.64898/2026.02.14.705918; this version posted February 17, 2026. The copyright holder for this preprint (which was not certified by peer review) is the author/funder, who has granted bioRxiv a license to display the preprint in perpetuity. It is made available under aCC-BY 4.0 International license.

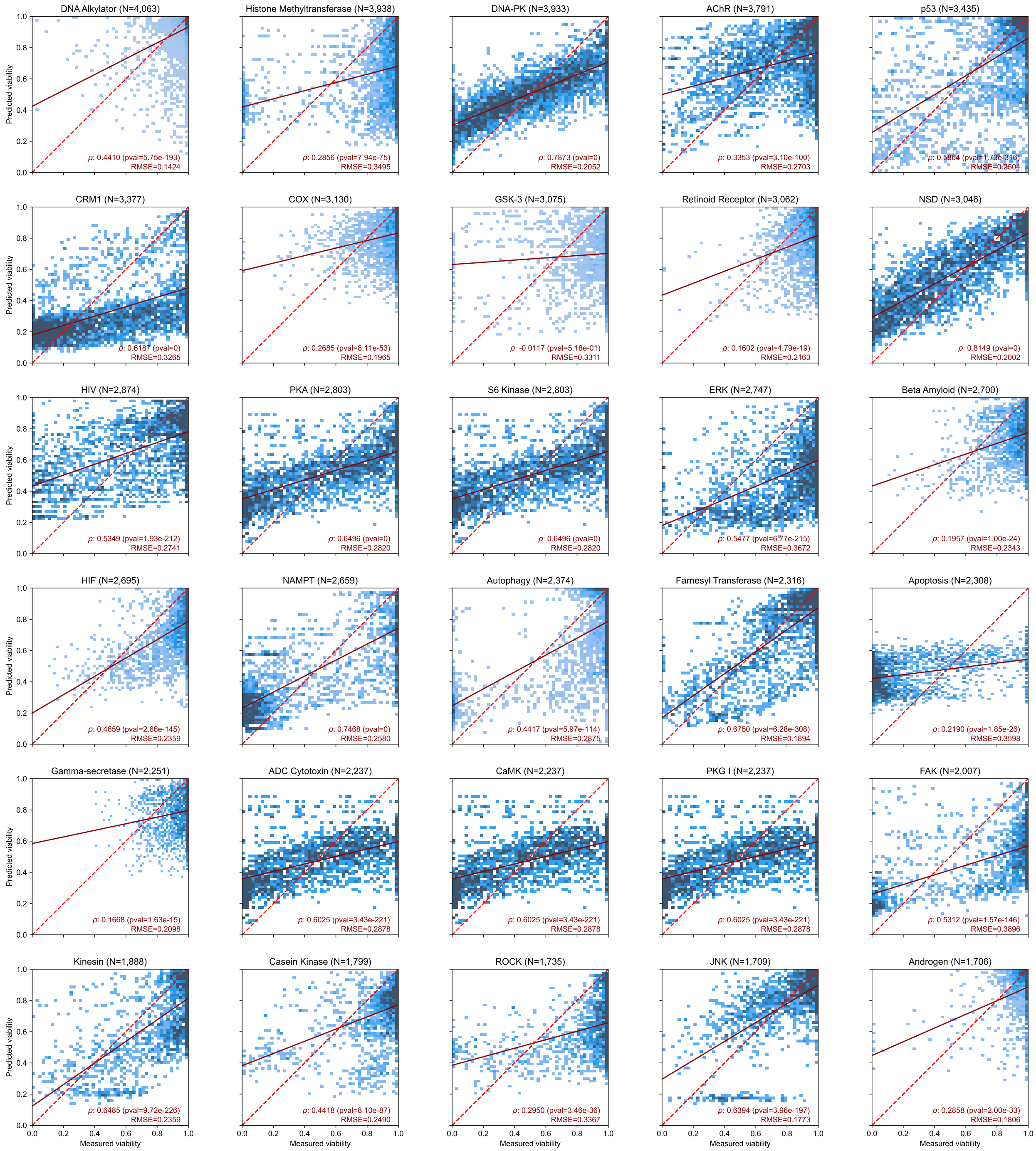

Figure S12: (3 of 4) Continuation of fig. S10.

bioRxiv preprint doi: https://doi.org/10.64898/2026.02.14.705918; this version posted February 17, 2026. The copyright holder for this preprint (which was not certified by peer review) is the author/funder, who has granted bioRxiv a license to display the preprint in perpetuity. It is made available under aCC-BY 4.0 International license.

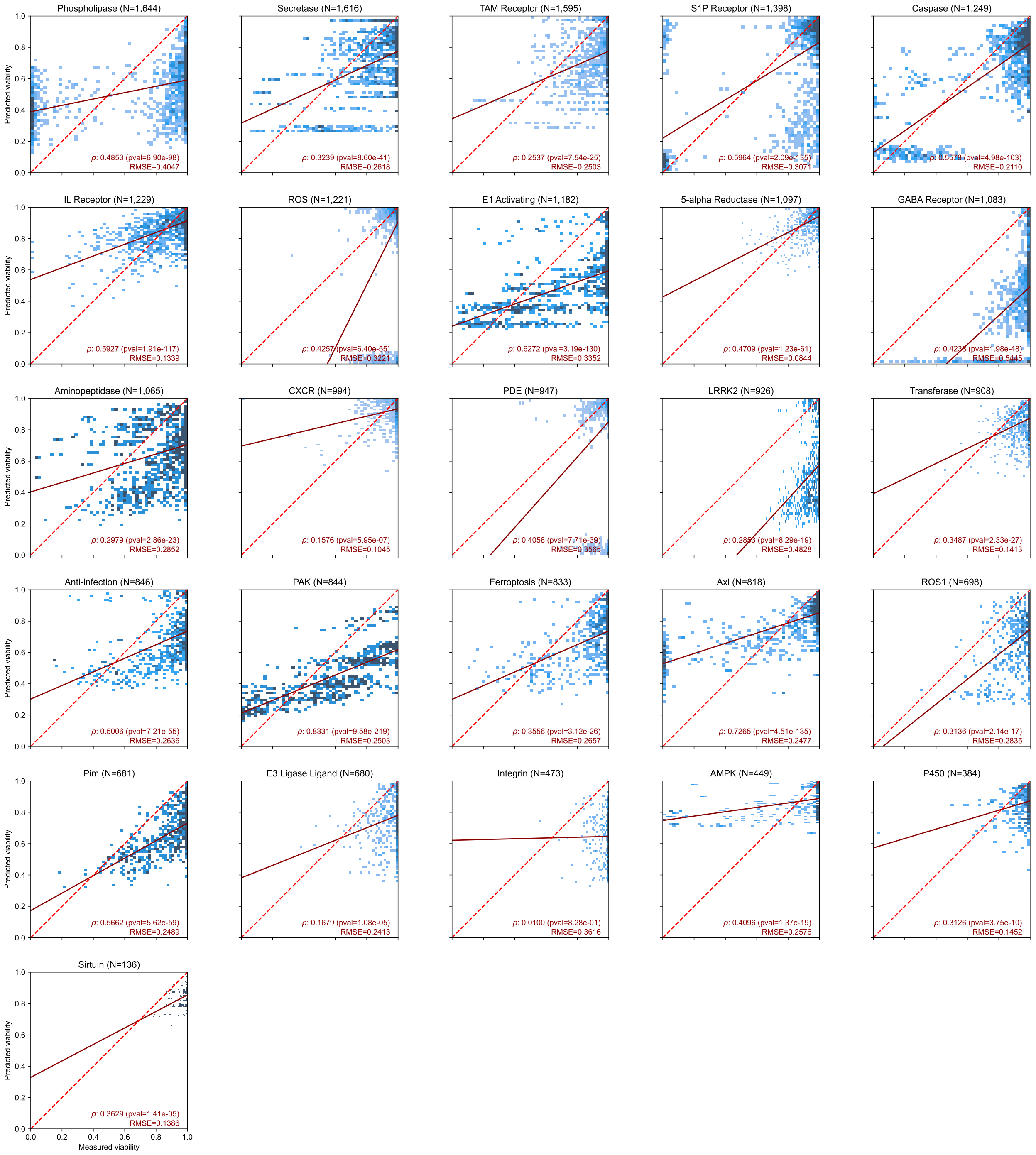

Figure S13: (4 of 4) Continuation of fig. S10.

bioRxiv preprint doi: https://doi.org/10.64898/2026.02.14.705918; this version posted February 17, 2026. The copyright holder for this preprint (which was not certified by peer review) is the author/funder, who has granted bioRxiv a license to display the preprint in perpetuity. It is made available under aCC-BY 4.0 International license.

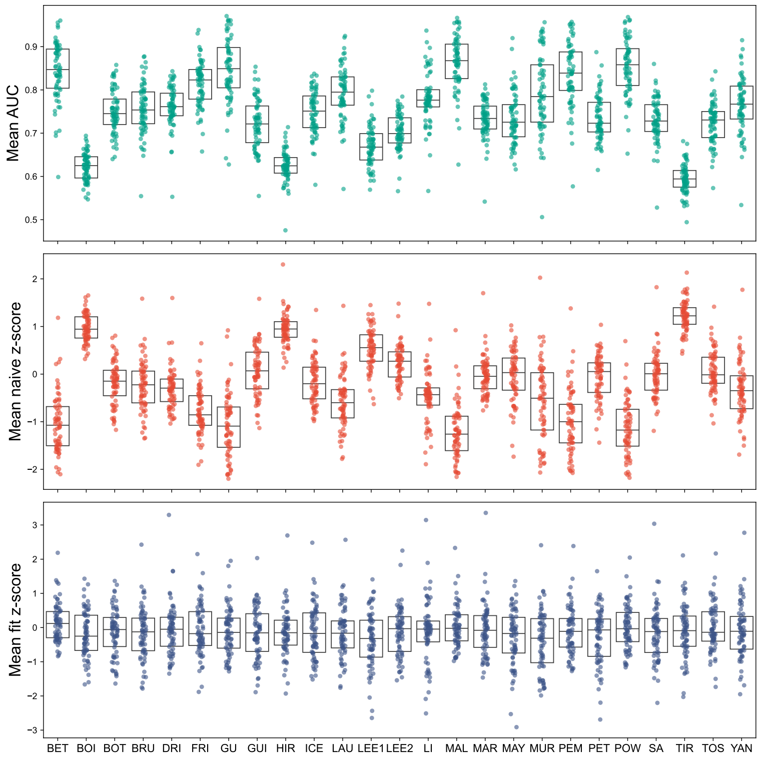

Figure S14: The Empirical Bayes method yields considerably diminished study-level batch effects on z-scores. Per-target AUCs (top), naive z-scores (middle) and empirically fit z-scores (bottom) across studies (study abbreviations: table S1).

bioRxiv preprint doi: https://doi.org/10.64898/2026.02.14.705918; this version posted February 17, 2026. The copyright holder for this preprint (which was not certified by peer review) is the author/funder, who has granted bioRxiv a license to display the preprint in perpetuity. It is made available under aCC-BY 4.0 International license.

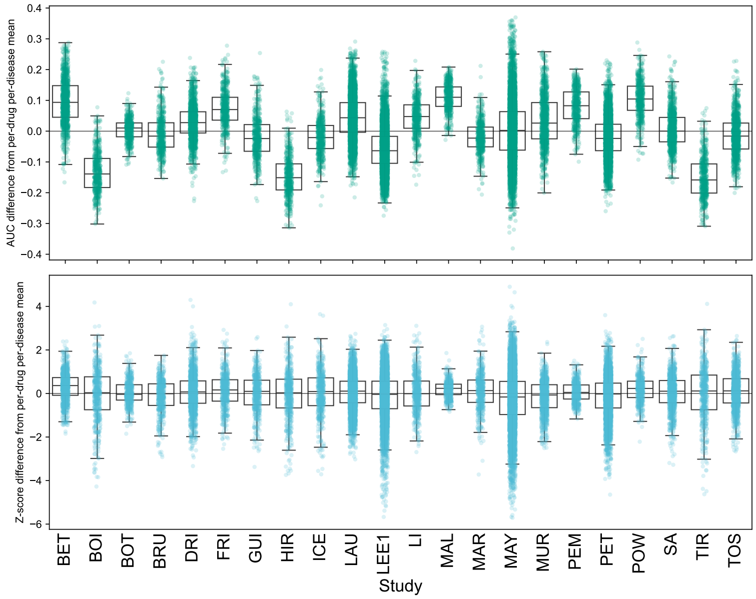

Figure S15: Raw AUC scores exhibit batch effects, which are attenuated by Empirical Bayesian z-scores. Per-drug per-disease differences from study to population means for raw AUC (top) and empirically fit z-scores (bottom). Each point represents a (drug, OncoTree) pair, and quantities are differences in the study's average score (AUC or z-score, respectively) on that (drug, disease) pair compared to the global average for that pair. The bottom plot shows considerably attenuated study-level bias compared to the top plot. (boxes: quartiles and medians; whiskers: 1.5 IQR; study abbreviations: table S1).

bioRxiv preprint doi: https://doi.org/10.64898/2026.02.14.705918; this version posted February 17, 2026. The copyright holder for this preprint (which was not certified by peer review) is the author/funder, who has granted bioRxiv a license to display the preprint in perpetuity. It is made available under aCC-BY 4.0 International license.

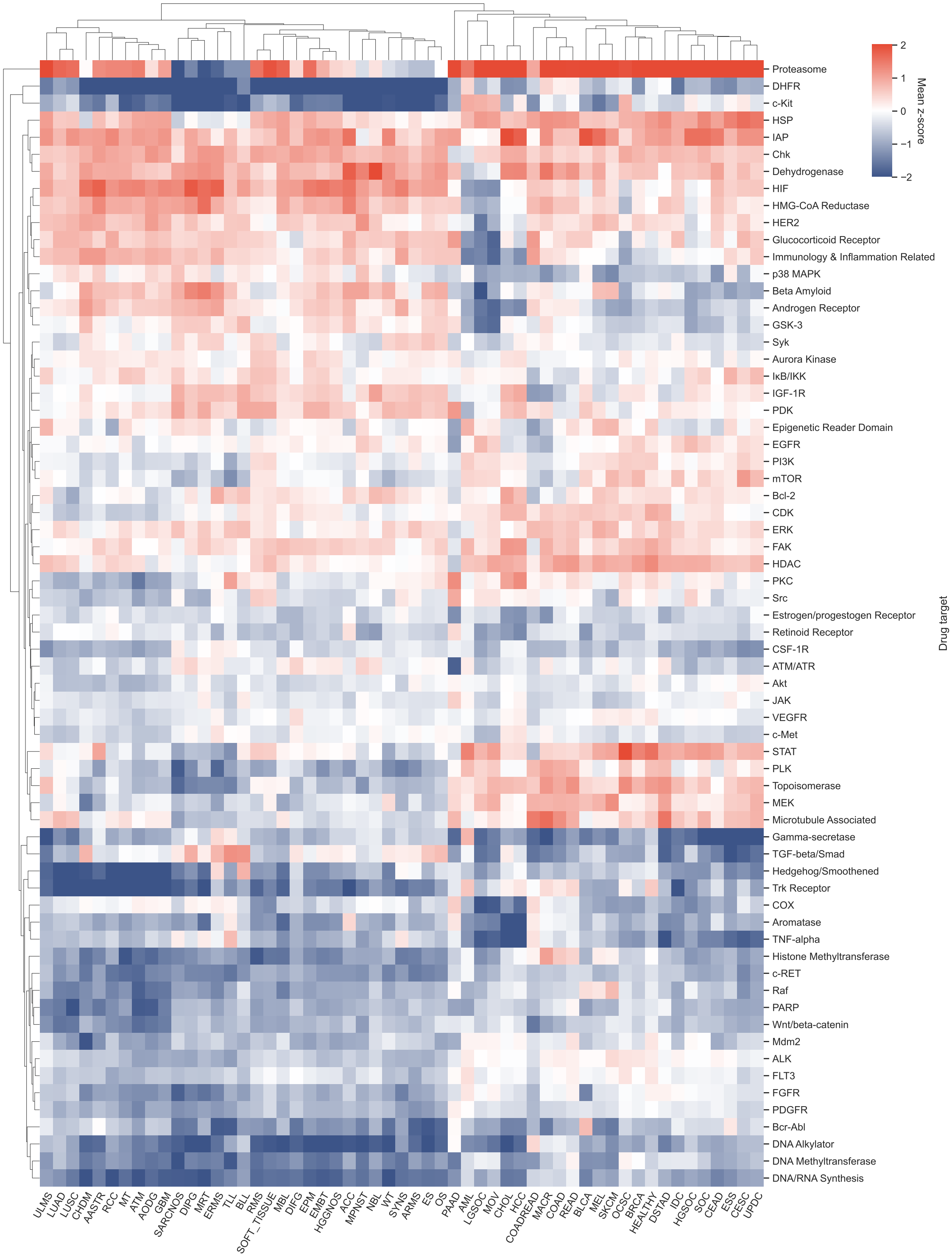

Figure S16: Imputed disease-target z-scores exhibit systematic response heterogeneity. Mean AUC-derived z-scores of model outputs for each target with at least 3 drugs and each disease with at least 3 samples. Rows and columns are clustered by average distance in Euclidean space.

bioRxiv preprint doi: https://doi.org/10.64898/2026.02.14.705918; this version posted February 17, 2026. The copyright holder for this preprint (which was not certified by peer review) is the author/funder, who has granted bioRxiv a license to display the preprint in perpetuity. It is made available under aCC-BY 4.0 International license.

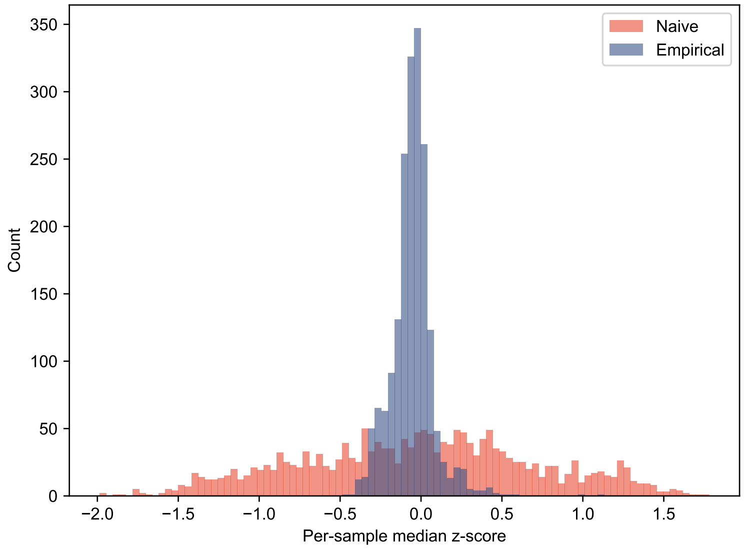

Figure S17: Empirical Bayes z-scores have considerably fewer skewed median per-sample values. Distribution of median z-scores per sample for naive z-score settings (red) and empirically fit z-scores (blue).

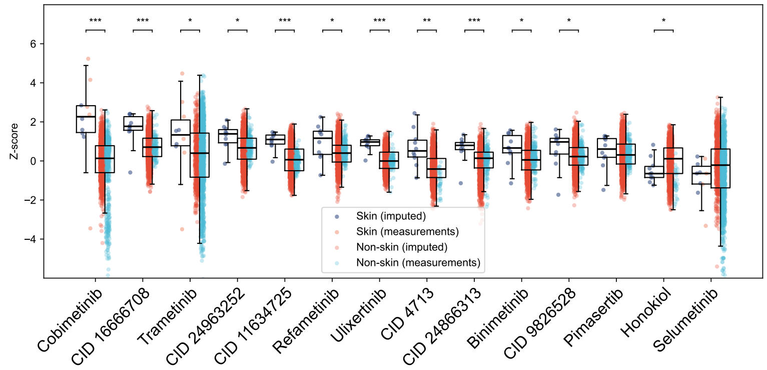

Figure S18: Fully-imputed z-scores mix with measurement-derived z-scores for MEK/ERK-targeting drugs across samples. Distribution of z-scores for all drugs labeled as targeting MEK/ERK, with z-scores from pure imputations (i.e. dose-response curves fully imputed) separated from z-scores derived from curves with measurements in the dataset. (BH-corrected p-values from two-sided Mann-Whitney U tests, *: $p < 0.1$; **: $p < 0.01$; ***: $p < 0.001$. Boxes: first and third quartiles; line: median; whiskers: $1.5 \times$ IQR).

bioRxiv preprint doi: https://doi.org/10.64898/2026.02.14.705918; this version posted February 17, 2026. The copyright holder for this preprint (which was not certified by peer review) is the author/funder, who has granted bioRxiv a license to display the preprint in perpetuity. It is made available under aCC-BY 4.0 International license.

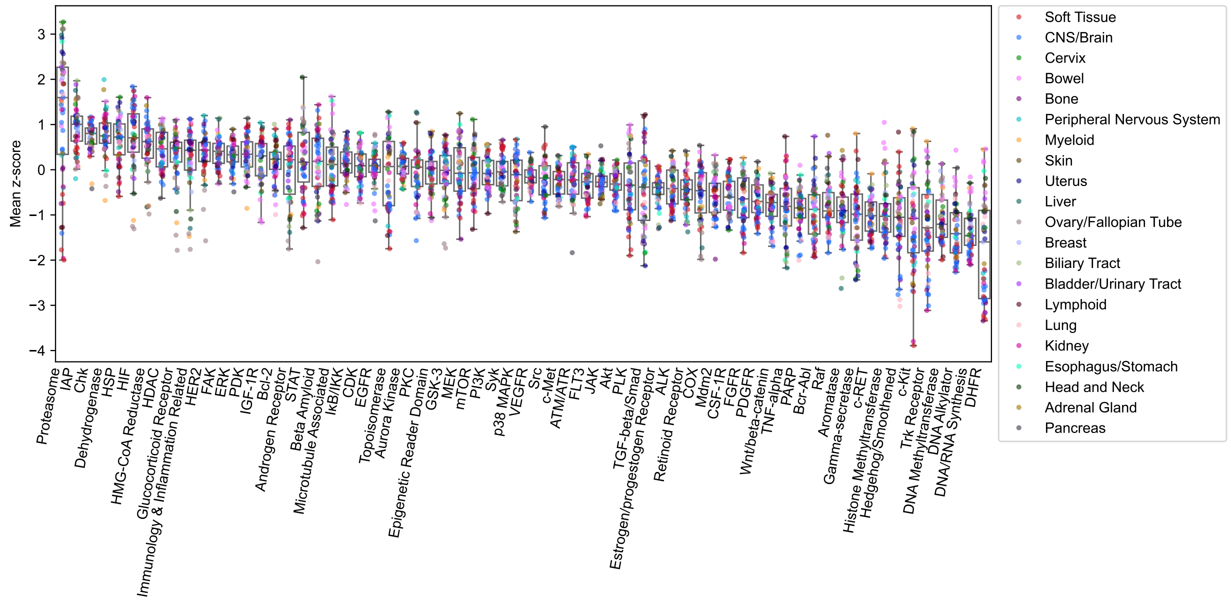

Figure S19: Per-tissue responses exhibit intra- and inter-group heterogeneity across drug targets. Mean z-scores per-target and per-primary-disease-tissue-type, grouped by target (boxes: first and third quartiles; line: median; whiskers: 1.5 × IQR).

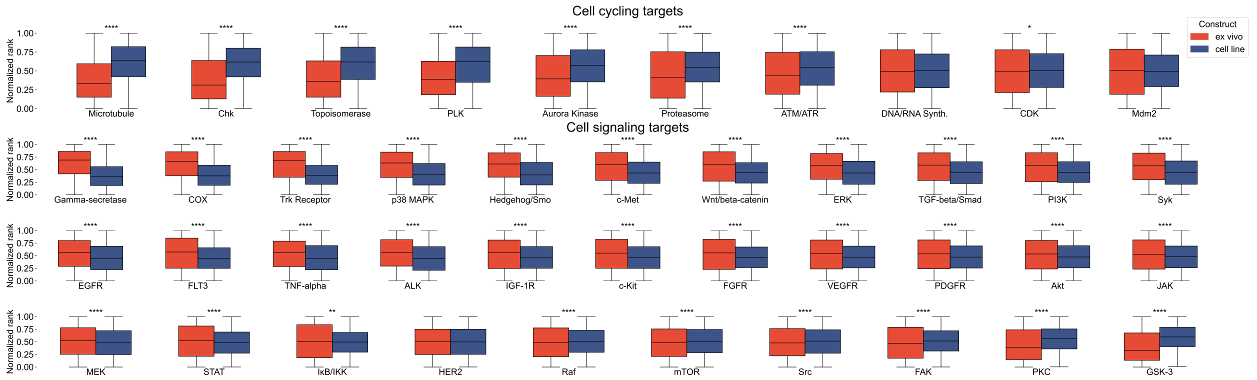

Figure S20: Cell-cycling targeting drugs are more comparatively efficacious on cell line constructs; signaling-targeting drugs are more comparatively efficacious on ex vivo constructs. Distributions of normalized ranks for cell cycling (top) and signaling (bottom) targets, via two curated subsets of the targets in the dataset (boxes: IQR; horizontal lines: medians; whiskers: 1.5 times IQR). Ranks were computed per drug, normalized to between 0 and 1 (higher is more efficacious). Asterisks indicate BH-adjusted significance from two-sided Welch's t-tests on ex vivo versus cell-line ranks. (*: $q < 0.1$; **: $q < 0.01$; ***: $q < 0.001$; ****: $q < 0.0001$).

bioRxiv preprint doi: https://doi.org/10.64898/2026.02.14.705918; this version posted February 17, 2026. The copyright holder for this preprint (which was not certified by peer review) is the author/funder, who has granted bioRxiv a license to display the preprint in perpetuity. It is made available under aCC-BY 4.0 International license.

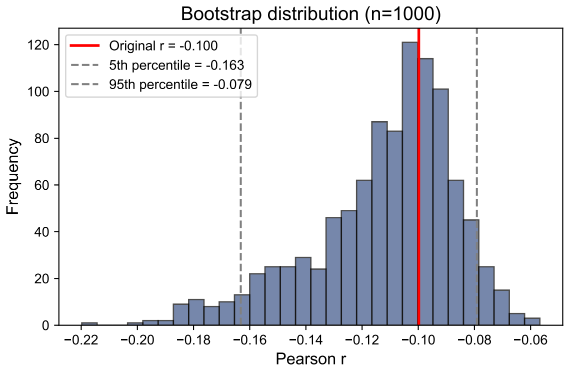

Figure S21: The correlation between RMSE difference and absolute z-score difference when adding cell line data is robust to data resampling. The Pearson correlation r value for the linear fit from per-disease/target absolute z-score difference to RMSE improvement, in Fig. 5, is stable under bootstrapped data resampling.

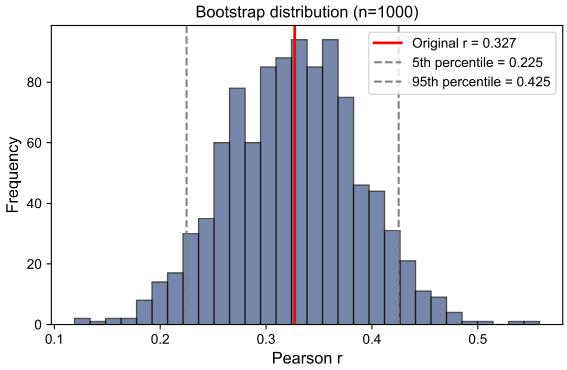

Figure S22: The correlation between TMB and nearest-neighbor TMB on cell line samples is robust to data resampling. The Pearson correlation r value for the linear fit from TMB to nearest-neighbor TMB (in log/log space), as given in Fig. 5, improvement, in Fig. 5, is stable under bootstrapped data resampling.

bioRxiv preprint doi: https://doi.org/10.64898/2026.02.14.705918; this version posted February 17, 2026. The copyright holder for this preprint (which was not certified by peer review) is the author/funder, who has granted bioRxiv a license to display the preprint in perpetuity. It is made available under aCC-BY 4.0 International license.

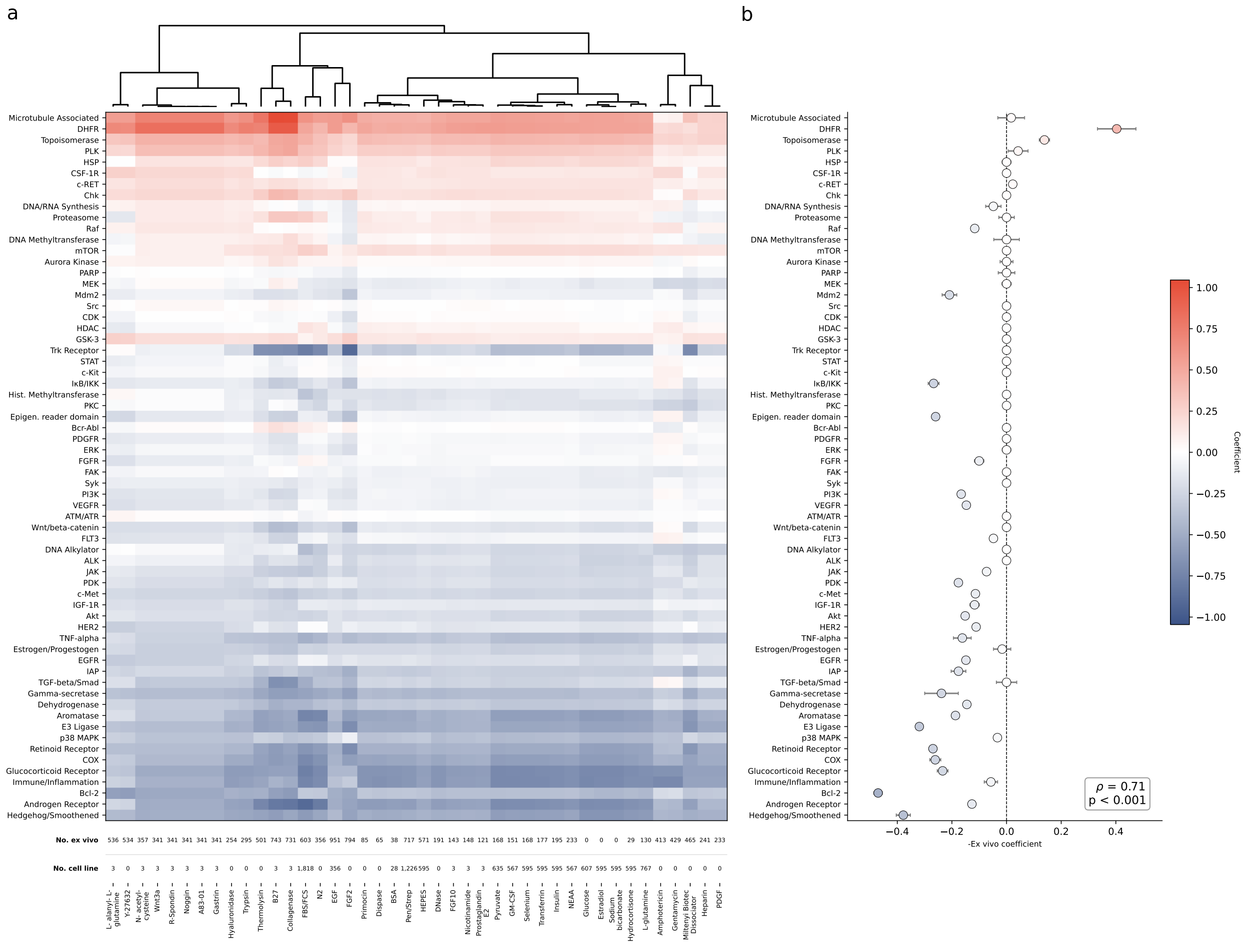

Figure S23: Patterns in ex vivo sensitivity remain consistent when controlling for media additives. (a) Average per sample z-scores by annotated drug target were regressed on the presence or absence of a media additive in a study and whether the sample was a cell line or an ex vivo construct. The negative coefficients on the one-hot encoded ex vivo covariate are shown here per drug target (vertical axis) and per additive (horizontal axis). Rows are ordered according to Fig. 5(e) and columns are grouped using hierarchical agglomerative clustering over Euclidean distance as shown in the dendrogram above. (b) The negative coefficients on the one-hot encoded ex vivo covariate are shown for ℓ_1-regularized multiple regressions to control for the effects of additives, construct type, and study ID simultaneously (point: coefficient; whiskers: bootstrapped standard errors of the mean). Spearman's ρ of the order of negative ex vivo coefficients to the row order of drug targets in Fig. 5(e) is shown. Full names for additive abbreviations and acronyms are available in table S6.

bioRxiv preprint doi: https://doi.org/10.64898/2026.02.14.705918; this version posted February 17, 2026. The copyright holder for this preprint (which was not certified by peer review) is the author/funder, who has granted bioRxiv a license to display the preprint in perpetuity. It is made available under aCC-BY 4.0 International license.

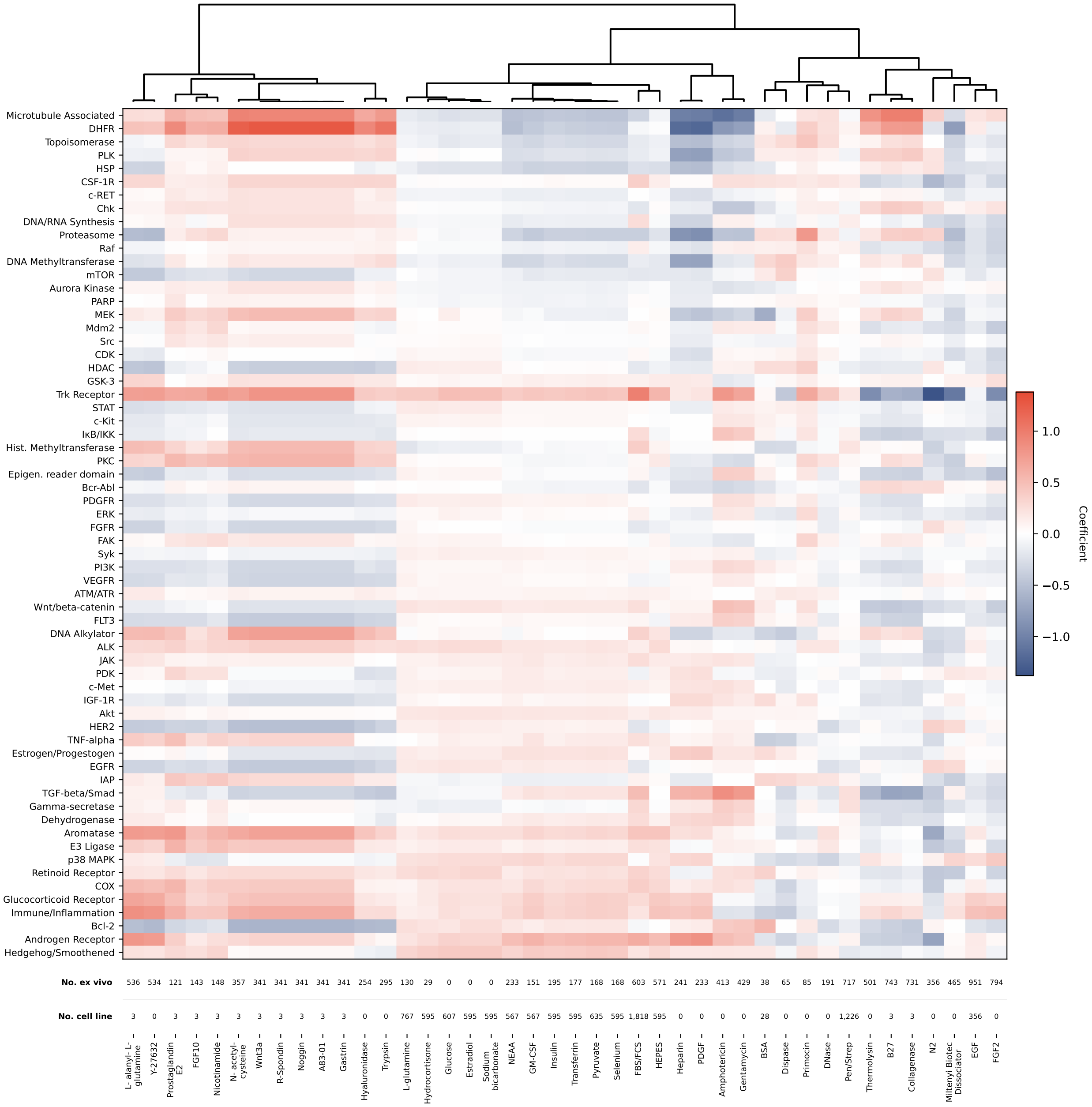

Figure S24: Additives partially explain drug target variability. Average per sample z-scores by annotated drug target were regressed on the presence or absence of a media additive in a study and whether the sample was a cell line or an ex vivo construct. The negative coefficients on the one-hot encoded media additive covariates are shown here per drug target (vertical axis) and per additive (horizontal axis). Rows are ordered according to Fig. 5(e) and columns are grouped using hierarchical agglomerative clustering over Euclidean distance as shown in the dendrogram above. Full names for additive abbreviations and acronyms are available in table S6.

bioRxiv preprint doi: https://doi.org/10.64898/2026.02.14.705918; this version posted February 17, 2026. The copyright holder for this preprint (which was not certified by peer review) is the author/funder, who has granted bioRxiv a license to display the preprint in perpetuity. It is made available under aCC-BY 4.0 International license.

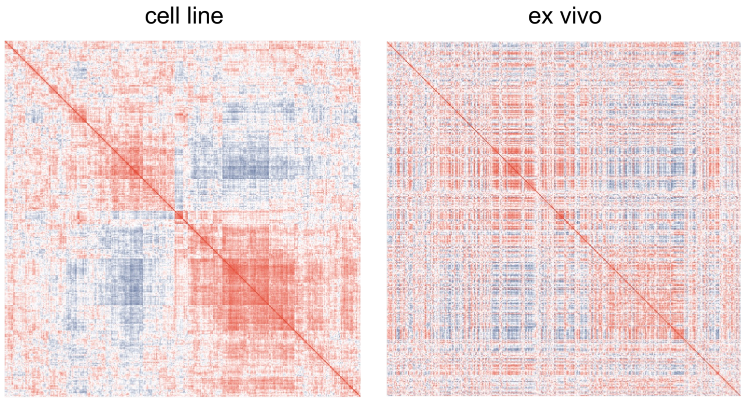

Figure S25: Pairwise drug correlation exhibits similar structure in cell line and ex vivo constructs. Pearson r values of mean z-scores calculated across OncoTree groupings exhibit similar blockwise structure, as quantified in Fig. 5.

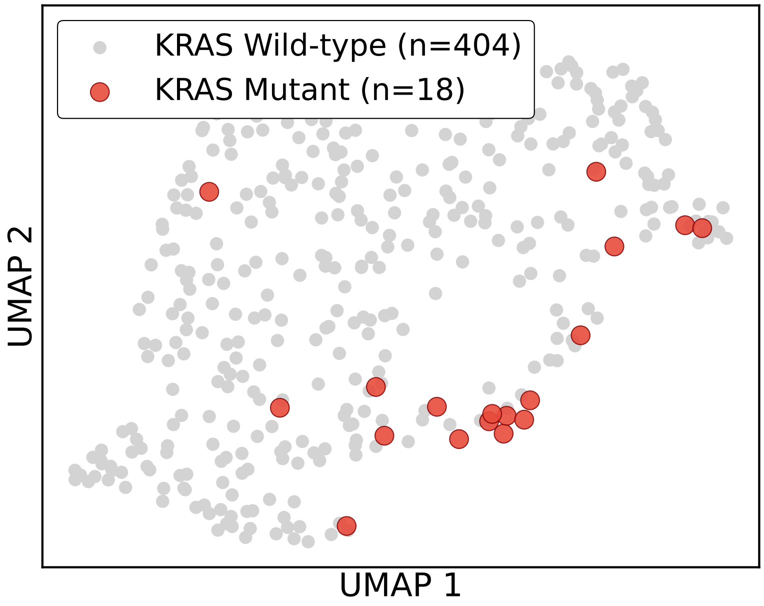

Figure S26: Foundation Model Embedding UMAP Visualization. Two-dimensional UMAP projection of sample embeddings generated by the PPC Foundation Model for the Beat AML cohort. KRAS-mutated samples are highlighted in red.

bioRxiv preprint doi: https://doi.org/10.64898/2026.02.14.705918; this version posted February 17, 2026. The copyright holder for this preprint (which was not certified by peer review) is the author/funder, who has granted bioRxiv a license to display the preprint in perpetuity. It is made available under aCC-BY 4.0 International license.

Abbreviation	Study
BET	Betge et al.[33]
BOI	Boilève et al.[35], Polit et al.[36]
BOT	Bottomly et al.[28]
BRU	Bruna et al.[38]
DRI	Driehuis et al.[4]
FRI	Friedman et al.[24]
GU	Gu et al.[25]
GUI	Guillen et al.[7]
HIR	Hirt et al.[11]
ICE	Ice et al.[40]
JOH	Johansson et al.[10]
LAU	Lau et al.[41]
LEE1	Lee et al.[9]
LEE2	Lee et al.[30]
LI	Li et al.[34]
MAL	Malani et al.[26]
MAR	Martins et al.[15]
MAY	Mayoh et al.[29]
MUR	Murumägi et al.[16]
PEM	Pemovska et al.[27]
PET	Peterziel et al.[20]
POW	Powell et al.[37]
SA	Sa et al.[14]
TIR	Tiriac et al.[1]
TOS	Toshimitsu et al.[32]
YAN	Yan et al.[31]

Table S1: Abbreviations used for studies.

bioRxiv preprint doi: https://doi.org/10.64898/2026.02.14.705918; this version posted February 17, 2026. The copyright holder for this preprint (which was not certified by peer review) is the author/funder, who has granted bioRxiv a license to display the preprint in perpetuity. It is made available under aCC-BY 4.0 International license.

AASTR	Anaplastic Astrocytoma	MFS	Myxofibrosarcoma
ACC	Adrenocortical Carcinoma	MNET	Miscellaneous Neuroepithelial Tumor
AML	Acute Myeloid Leukemia	MNG	Meningioma
AMPCA	Ampullary Carcinoma	MNGT	Meningothelial Tumor
AOAST	Anaplastic Oligoastrocytoma	MNM	Myeloid Neoplasm
AODG	Anaplastic Oligodendroglioma	MOV	Mucinous Ovarian Cancer
APE	Anaplastic Ependymoma	MPNST	Malignant Peripheral Nerve Sheath Tumor
ARMS	Alveolar Rhabdomyosarcoma	MRLS	Myxoid/Round-Cell Liposarcoma
ATM	Atypical Meningioma	MRT	Rhabdoid Cancer
ATRT	Atypical Teratoid/Rhabdoid Tumor	MRTL	Malignant Rhabdoid Tumor of the Liver
BGCT	Germ Cell Tumor, Brain	MSTAD	Mucinous Stomach Adenocarcinoma
BL	Burkitt Lymphoma	MT	Malignant Tumor
BLCA	Bladder Urothelial Carcinoma	MXOV	Mixed Ovarian Carcinoma
BLL	B-Lymphoblastic Leukemia/Lymphoma	NBL	Neuroblastoma
BLSC	Bladder Squamous Cell Carcinoma	NSCLC	Non-Small Cell Lung Cancer
BRCA	Invasive Breast Carcinoma	NST	Nerve Sheath Tumor
CCOV	Clear Cell Ovarian Cancer	OCSC	Oral Cavity Squamous Cell Carcinoma
CEAD	Cervical Adenocarcinoma	ODG	Oligodendroglioma
CESC	Cervical Squamous Cell Carcinoma	OOVC	Ovarian Cancer, Other
CHDM	Chordoma	OPHSC	Oropharynx Squamous Cell Carcinoma
CHGL	Chordoid Glioma of the Third Ventricle	OS	Osteosarcoma
CHOL	Cholangiocarcinoma	OSACA	Salivary Carcinoma, Other
CHS	Chondrosarcoma	OVT	Ovarian Epithelial Tumor
COAD	Colon Adenocarcinoma	PAAD	Pancreatic Adenocarcinoma
COADREAD	Colorectal Adenocarcinoma	PACT	Cystic Tumor of the Pancreas
CPC	Choroid Plexus Carcinoma	PCNSM	Primary CNS Melanoma
CPT	Choroid Plexus Tumor	PCNSMT	Primary CNS Melanocytic Tumors
DASTR	Diffuse Astrocytoma	PHC	Pheochromocytoma
DCS	Dendritic Cell Sarcoma	PNET	Primitive Neuroectodermal Tumor
DIFG	Diffuse Glioma	RCC	Renal Cell Carcinoma
DIPG	Diffuse Intrinsic Pontine Glioma	READ	Rectal Adenocarcinoma
DLBCLNOS	Diffuse Large B-Cell Lymphoma, NOS	RMS	Rhabdomyosarcoma
DSTAD	Diffuse Type Stomach Adenocarcinoma	SACA	Salivary Carcinoma
ECAD	Endocervical Adenocarcinoma	SARCNOS	Sarcoma, NOS
EGC	Esophagogastric Adenocarcinoma	SCLC	Small Cell Lung Cancer
EMBT	Embryonal Tumor	SCST	Sex Cord Stromal Tumor
EOV	Endometrioid Ovarian Cancer	SFTCNS	Solitary Fibrous Tumor of the Central Nervous System
EPM	Ependymoma	SKCM	Cutaneous Melanoma
EPMT	Ependymomal Tumor	SOC	Serous Ovarian Cancer
ERMS	Embryonal Rhabdomyosarcoma	SOFT_TISSUE	Soft Tissue
ES	Ewing Sarcoma	SYNS	Synovial Sarcoma
ESS	Endometrial Stromal Sarcoma	TGCT	Tenosynovial Giant Cell Tumor Diffuse Type
ESST	Ewing Sarcoma of Soft Tissue	THFO	Follicular Thyroid Cancer
GBM	Glioblastoma Multiforme	THPA	Papillary Thyroid Cancer
GRCT	Granulosa Cell Tumor	TLL	T-Lymphoblastic Leukemia/Lymphoma
GSARC	Gliosarcoma	UCEC	Endometrial Carcinoma
HCC	Hepatocellular Carcinoma	UCS	Uterine Carcinosarcoma
HDCN	Histiocytic and Dendritic Cell Neoplasms	UEC	Uterine Endometrioid Carcinoma
HDCS	Histiocytic Dendritic Cell Sarcoma	ULM	Uterine Leiomyoma
HGGNOS	High-Grade Glioma, NOS	ULMS	Uterine Leiomyosarcoma
HGSOC	High-Grade Serous Ovarian Cancer	UPDC	Poorly Differentiated Carcinoma of the Uterus
HNSC	Head and Neck Squamous Cell Carcinoma	USARC	Uterine Sarcoma/Mesenchymal
IAMPCA	Intestinal Ampullary Carcinoma	WDTC	Well-Differentiated Thyroid Cancer
IBC	Inflammatory Breast Cancer	WT	Wilms' Tumor
IDC	Breast Invasive Ductal Carcinoma		
IPMN	Intraductal Papillary Mucinous Neoplasm		
IPN	Intraductal Papillary Neoplasm of the Bile Duct		
ISTAD	Intestinal Type Stomach Adenocarcinoma		
JMML	Juvenile Myelomonocytic Leukemia		
LGSOC	Low-Grade Serous Ovarian Cancer		
LIHB	Hepatoblastoma		
LIPO	Liposarcoma		
LMS	Leiomyosarcoma		
LNET	Lung Neuroendocrine Tumor		
LNM	Lymphoid Neoplasm		
LUAD	Lung Adenocarcinoma		
LUNE	Large Cell Neuroendocrine Carcinoma		
LUNG	Lung		
LUSC	Lung Squamous Cell Carcinoma		
MACR	Mucinous Adenocarcinoma of the Colon and Rectum		
MBL	Medulloblastoma		
MBLNWS	Medulloblastoma, Non-WNT, Non-SHH		
MBT	Miscellaneous Brain Tumor		
MEL	Melanoma		
MFH	Undifferentiated Pleomorphic Sarcoma		

Table S2: Disease abbreviations. All abbreviations are standard OncoTree[42] codes.

bioRxiv preprint doi: https://doi.org/10.64898/2026.02.14.705918; this version posted February 17, 2026. The copyright holder for this preprint (which was not certified by peer review) is the author/funder, who has granted bioRxiv a license to display the preprint in perpetuity. It is made available under aCC-BY 4.0 International license.

Table S3: Kinase target annotations for inhibitor agerafenib. Annotations are from various resources compared to published target affinity. Checkmarks denote proteins adjudicated by a given resource to be targets of the drug, while crosses denote proteins that are not indicated to be targets. The "Assay affinity" column denotes published affinity estimates[162].

Target	DrugBank	OpenTargets	PubChem	Selleck Chem	Assay affinity
CSF-1R	✗	✗	✗	✓	✓
c-Kit	✗	✗	✗	✓	✓
EGFR	✗	✓	✓	✗	✗
EPHA2	✗	✗	✓	✗	✗
PDGFR	✗	✗	✗	✓	✓
RAF	✓	✓	✓	✓	✓
RET	✓	✓	✓	✓	✓

Table S4: Broad target annotation groupings for cell signaling and cell cycling comparisons.

Category	Targets
Cell Signaling	Akt, PI3K, mTOR, Raf, FGFR, IGF-1R, Trk Receptor, c-Met, MEK, EGFR, HER2, Src, c-Kit, PKC, Wnt/beta-catenin, ALK, PDGFR, VEGFR, JAK, ERK, Gamma-secretase, TGF-beta/Smad, Syk, Hedgehog/Smoothened, STAT, TNF-alpha, c-RET, FLT3, CSF-1R, p38 MAPK, FAK, IκB/IKK, GSK-3, COX
Cell Cycling	ATM/ATR, Aurora Kinase, CDK, Chk, DNA/RNA Synthesis, Mdm2, Microtubule Associated, PLK, Proteasome, Topoisomerase

Table S5: Coarse groupings for drug target annotations.

Group	Targets
EGFR	EGFR, HER2
FGFR/PDGFR/VEGFR	FGFR, PDGFR, VEGFR, c-Kit, c-RET, CSF-1R
IGF1R/MET	c-Met, IGF-1R
ALK/TRK	Trk Receptor, ALK
BCR-ABL/FLT3/AURK	Aurora Kinase, Bcr-Abl, FLT3
Wnt/Hedgehog/Smo	Wnt/beta-catenin, Hedgehog/Smoothened
PI3K/AKT/mTOR	Akt, mTOR, PI3K
TNFa/TGFb	TNF-alpha, TGF-beta/Smad
MEK/ERK	ERK, MEK
DNA synth/damage/repair	DHFR, DNA/RNA Synthesis, PLK, PARP, DNA Methyltransferase, Topoisomerase, Microtubule Associated
Cell cycle arrest	PKC, Chk, Syk, CDK
Protein stability/degradation	E3 Ligase, Proteasome, HSP
Anti-androgenic	Estrogen/progestogen Receptor, Androgen Receptor, Aromatase
Metabolic	Glucocorticoid Receptor, HMG-CoA Reductase, Dehydrogenase

bioRxiv preprint doi: https://doi.org/10.64898/2026.02.14.705918; this version posted February 17, 2026. The copyright holder for this preprint (which was not certified by peer review) is the author/funder, who has granted bioRxiv a license to display the preprint in perpetuity. It is made available under aCC-BY 4.0 International license.

Table S6: Abbreviations and acronyms for media additives.

Abbreviation/acronym	Full name
BSA	Bovine serum albumin
EGF	Epidermal growth factor
FBS	Fetal bovine serum
FCS	Fetal calf serum
FGF2	Fibroblast growth factor 2
FGF10	Fibroblast growth factor 10
GM-CSF	Granulocyte-macrophage colony-stimulating factor
HEPES	4-(2-hydroxyethyl)-1-piperazineethanesulfonic acid
NEAA	Non-essential amino acids
PDGF	Platelet-derived growth factor
Pen/Strep	Penicillin-Streptomycin

bioRxiv preprint doi: https://doi.org/10.64898/2026.02.14.705918; this version posted February 17, 2026. The copyright holder for this preprint (which was not certified by peer review) is the author/funder, who has granted bioRxiv a license to display the preprint in perpetuity. It is made available under aCC-BY 4.0 International license.

Supplementary Algorithm S1: Cross-study drug harmonization.

Require: Graph G containing drugs from all studies, Associative array A

```
for all drug ∈ G do
  metadata ← PUGExactCompoundNameLookup(drug)
  if metadata = null then
    metadata ← PUGExactSubstanceNameLookup(drug)
  end if
  if metadata = null then
    metadata ← PUGSearchCompoundLookup(drug)
  end if
  if metadata = null then
    metadata ← PUGSearchSubstanceLookup(drug)
  end if
  A[drug] ← metadata
end for

for all drugA, drugB ∈ G, drugA ≠ drugB do
  if GetChemicalFormula(A[drugA]) = GetChemicalFormula(A[drugB]) then
    AddEdge(G, drugA, drugB)
  end if
  if GetPubchemID(A[drugA]) = GetPubchemID(A[drugB]) then
    AddEdge(G, drugA, drugB)
  end if
  if GetPrimaryCompoundPubchemID(A[drugA]) = GetPubchemID(A[drugB]) then
    AddEdge(G, drugA, drugB)
  end if
  if GetPrimaryCompoundPubchemID(A[drugB]) = GetPubchemID(A[drugA]) then
    AddEdge(G, drugA, drugB)
  end if
  if GetPrimaryCompoundPubchemID(A[drugA]) =
  GetPrimaryCompoundPubchemID(A[drugB]) then
    AddEdge(G, drugA, drugB)
  end if
end for

for all G′ ∈ ConnectedComponents(G) do
  id ← NextRandomId()
  for all drug ∈ G′ do
    drug.id ← id
  end for
end for
```
